# Supplementary material for: Brown Adipose Tissue Sheds Extracellular Vesicles That Carry Potential Biomarkers of Metabolic and Thermogenesis Activity Which Are Affected by High Fat Diet Intervention
Source: Int J Mol Sci. 2022 Sep 16;23(18):10826. doi: 10.3390/ijms231810826 (PMC9504916; doi:10.3390/ijms231810826)
Supplement: Supplementary file 1 [file ijms-23-10826-s001.zip › Supplementary Materials file 1.pdf]

Pathway Analysis Report

This report contains the pathway analysis results for the submitted sample ''. Analysis was performed against Reactome version 81 on 27/07/2022. The web link to these results is:

https://reactome.org/PathwayBrowser/#/ANALYSIS=MjAyMjA3MjYxMTQ5MjhfMjAyNTg%3D

Please keep in mind that analysis results are temporarily stored on our server. The storage period depends on usage of the service but is at least 7 days. As a result, please note that this URL is only valid for a limited time period and it might have expired.

Table of Contents

1. Introduction
2. Properties
3. Genome-wide overview
4. Most significant pathways
5. Pathways details
6. Identifiers found
7. Identifiers not found

1. Introduction

Reactome is a curated database of pathways and reactions in human biology. Reactions can be considered as pathway 'steps'. Reactome defines a 'reaction' as any event in biology that changes the state of a biological molecule. Binding, activation, translocation, degradation and classical biochemical events involving a catalyst are all reactions. Information in the database is authored by expert biologists, entered and maintained by Reactome's team of curators and editorial staff. Reactome content frequently cross-references other resources e.g. NCBI, Ensembl, UniProt, KEGG (Gene and Compound), ChEBI, PubMed and GO. Orthologous reactions inferred from annotation for Homo sapiens are available for 17 non-human species including mouse, rat, chicken, puffer fish, worm, fly, yeast, rice, and Arabidopsis. Pathways are represented by simple diagrams following an SBGN-like format.

Reactome's annotated data describe reactions possible if all annotated proteins and small molecules were present and active simultaneously in a cell. By overlaying an experimental dataset on these annotations, a user can perform a pathway over-representation analysis. By overlaying quantitative expression data or time series, a user can visualize the extent of change in affected pathways and its progression. A binomial test is used to calculate the probability shown for each result, and the p-values are corrected for the multiple testing (Benjamini–Hochberg procedure) that arises from evaluating the submitted list of identifiers against every pathway.

To learn more about our Pathway Analysis, please have a look at our relevant publications:

Fabregat A, Sidiropoulos K, Garapati P, Gillespie M, Hausmann K, Haw R, … D'Eustachio P (2016). The reactome pathway knowledgebase. Nucleic Acids Research, 44(D1), D481–D487. https://doi.org/10.1093/nar/gkv1351.

Fabregat A, Sidiropoulos K, Viteri G, Forner O, Marin-Garcia P, Arnau V, … Hermjakob H (2017). Reactome pathway analysis: a high-performance in-memory approach. BMC Bioinformatics, 18.

2. Properties

- This is an **overrepresentation** analysis: A statistical (hypergeometric distribution) test that determines whether certain Reactome pathways are over-represented (enriched) in the submitted data. It answers the question 'Does my list contain more proteins for pathway X than would be expected by chance?' This test produces a probability score, which is corrected for false discovery rate using the Benjamani-Hochberg method.
- 26 out of 28 identifiers in the sample were found in Reactome, where 151 pathways were hit by at least one of them.
- All non-human identifiers have been converted to their human equivalent.
- This report is filtered to show only results for species 'Homo sapiens' and resource 'all resources'.
- The unique ID for this analysis (token) is MjAyMjA3MjYxMTQ5MjhfMjAyNTg%3D. This ID is valid for at least 7 days in Reactome's server. Use it to access Reactome services with your data.

3. Genome-wide overview

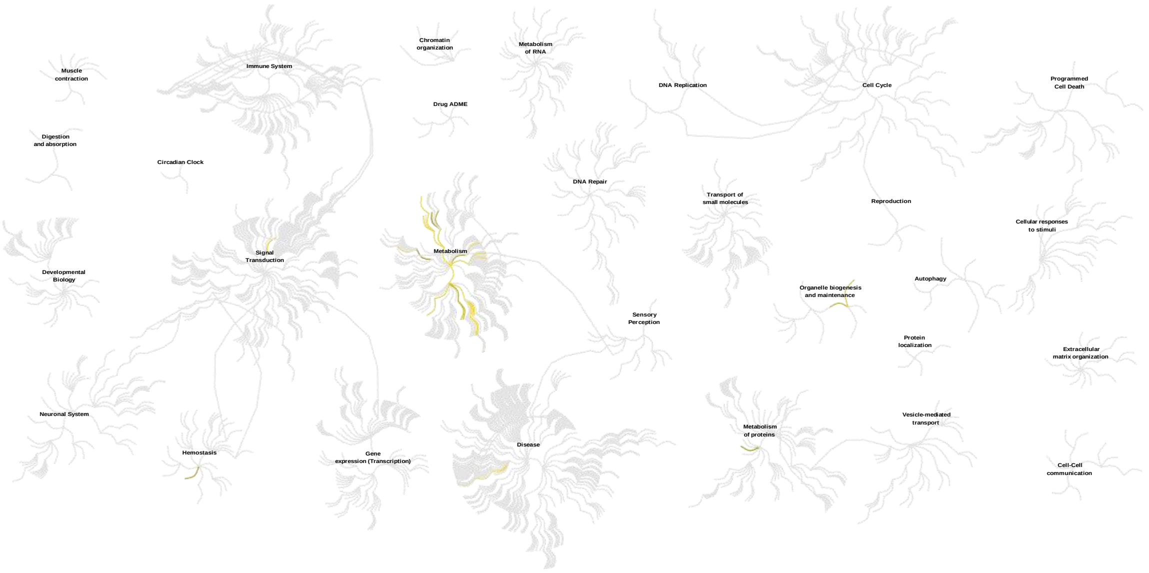

This figure shows a genome-wide overview of the results of your pathway analysis. Reactome pathways are arranged in a hierarchy. The center of each of the circular "bursts" is the root of one top-level pathway, for example "DNA Repair". Each step away from the center represents the next level lower in the pathway hierarchy. The color code denotes over-representation of that pathway in your input dataset. Light grey signifies pathways which are not significantly over-represented.

4. Most significant pathways

The following table shows the 25 most relevant pathways sorted by p-value.

Pathway name	Entities				Reactions	
	found	ratio	p-value	FDR*	found	ratio
The citric acid (TCA) cycle and respiratory electron transport	7 / 238	0.016	2.10e-07	3.17e-05	11 / 67	0.005
Pyruvate metabolism and Citric Acid (TCA) cycle	5 / 100	0.007	1.09e-06	8.16e-05	6 / 36	0.003
Metabolism	18 / 3,641	0.24	7.23e-06	3.61e-04	40 / 2,268	0.163
Fatty acid metabolism	6 / 431	0.028	1.17e-04	0.003	9 / 220	0.016
ChREBP activates metabolic gene expression	2 / 9	5.94e-04	1.32e-04	0.003	2 / 6	4.31e-04
Pyruvate metabolism	3 / 57	0.004	1.63e-04	0.003	4 / 15	0.001
Defective HLCS causes multiple carboxylase deficiency	2 / 10	6.60e-04	1.63e-04	0.003	2 / 4	2.87e-04
Defects in biotin (Btn) metabolism	2 / 12	7.92e-04	2.34e-04	0.004	2 / 6	4.31e-04
Biotin transport and metabolism	2 / 19	0.001	5.82e-04	0.009	5 / 13	9.33e-04
Beta oxidation of decanoyl-CoA to octanoyl-CoA-CoA	2 / 21	0.001	7.10e-04	0.009	2 / 5	3.59e-04
mitochondrial fatty acid beta-oxidation of unsaturated fatty acids	2 / 21	0.001	7.10e-04	0.009	2 / 6	4.31e-04
Metabolism of lipids	9 / 1,446	0.095	8.41e-04	0.01	16 / 965	0.069
Mitochondrial Fatty Acid Beta-Oxidation	3 / 105	0.007	9.59e-04	0.011	5 / 47	0.003
Carnitine metabolism	2 / 28	0.002	0.001	0.013	2 / 9	6.46e-04
Regulation of pyruvate dehydrogenase (PDH) complex	2 / 30	0.002	0.001	0.014	2 / 4	2.87e-04
Defects in vitamin and cofactor metabolism	2 / 42	0.003	0.003	0.022	2 / 20	0.001
Respiratory electron transport, ATP synthesis by chemiosmotic coupling, and heat production by uncoupling proteins.	3 / 153	0.01	0.003	0.022	5 / 31	0.002
Citric acid cycle (TCA cycle)	2 / 50	0.003	0.004	0.029	2 / 17	0.001
PPARA activates gene expression	3 / 175	0.012	0.004	0.029	3 / 41	0.003
Regulation of lipid metabolism by PPARalpha	3 / 177	0.012	0.004	0.029	3 / 45	0.003
mitochondrial fatty acid beta-oxidation of saturated fatty acids	2 / 53	0.004	0.004	0.031	3 / 29	0.002
Branched-chain amino acid catabolism	2 / 58	0.004	0.005	0.031	2 / 23	0.002

Pathway name	Entities				Reactions	
	found	ratio	p-value	FDR*	found	ratio
Metabolism of amino acids and derivatives	5 / 666	0.044	0.007	0.042	5 / 285	0.02
Signaling by Retinoic Acid	2 / 73	0.005	0.008	0.049	1 / 22	0.002
Glyoxylate metabolism and glycine degradation	2 / 73	0.005	0.008	0.049	1 / 23	0.002

* False Discovery Rate

5. Pathways details

For every pathway of the most significant pathways, we present its diagram, as well as a short summary, its bibliography and the list of inputs found in it.

1. The citric acid (TCA) cycle and respiratory electron transport (R-HSA-1428517)

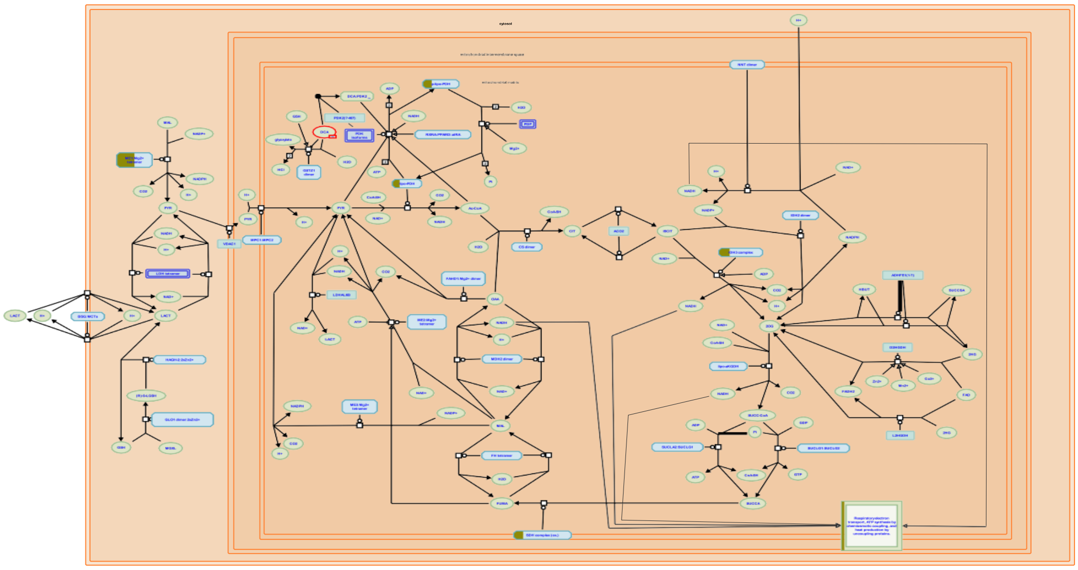

The metabolism of pyruvate provides one source of acetyl-CoA which enters the citric acid (TCA, tricarboxylic acid) cycle to generate energy and the reducing equivalent NADH. These reducing equivalents are re-oxidized back to NAD+ in the electron transport chain (ETC), coupling this process with the export of protons across the inner mitochondrial membrane. The chemiosmotic gradient created is used to drive ATP synthesis.

References

Edit history

Date	Action	Author
2003-11-03	Authored	Birney E, Schmidt EE, D'Eustachio P
2011-07-07	Edited	Jassal B
2011-07-07	Created	Jassal B
2022-05-21	Modified	Weiser JD

7 submitted entities found in this pathway, mapping to 7 Reactome entities

Input	UniProt Id	Input	UniProt Id	Input	UniProt Id
ATPB_HUMAN	P06576	IDH3A_HUMAN	P50213	MAOX_HUMAN	P48163

Input	UniProt Id	Input	UniProt Id	Input	UniProt Id
ODP2_HUMAN	P10515	ODPA_HUMAN	P08559	QCR6_HUMAN	P07919

2. Pyruvate metabolism and Citric Acid (TCA) cycle (R-HSA-71406)

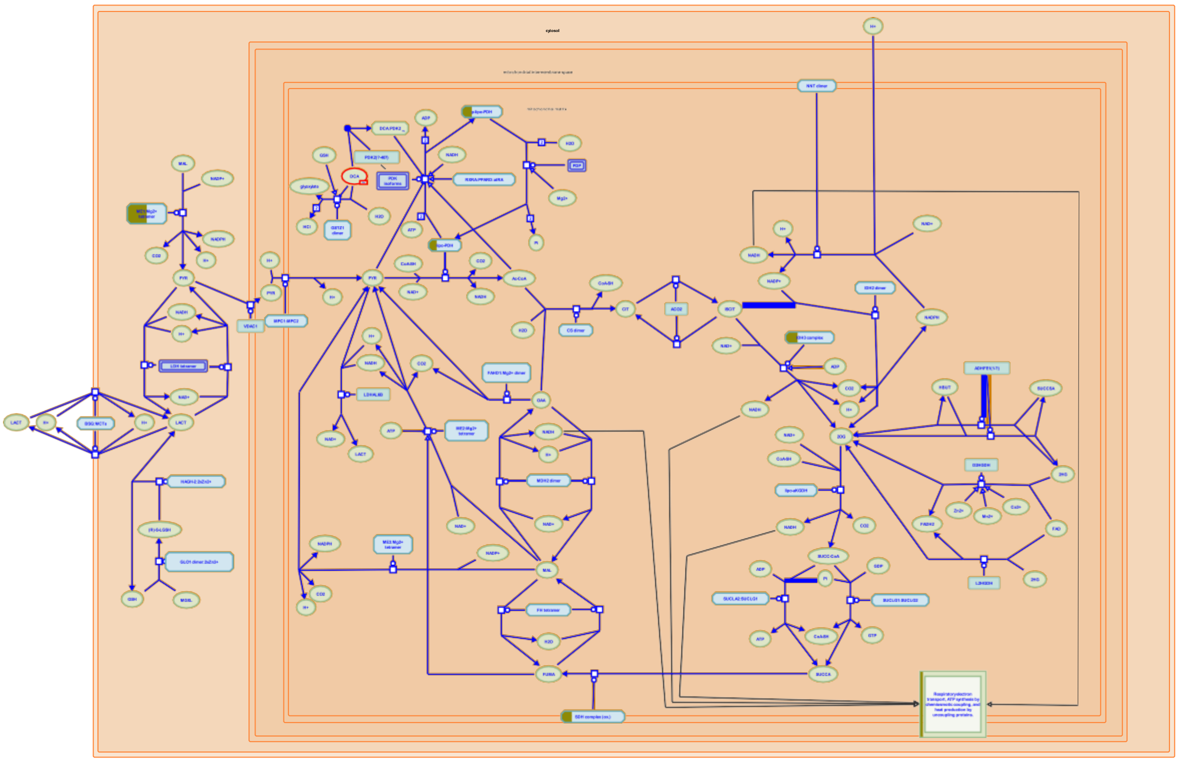

Pyruvate metabolism and the citric acid (TCA) cycle together link the processes of energy metabolism in a human cell with one another and with key biosynthetic reactions. Pyruvate, derived from the reversible oxidation of lactate or transamination of alanine, can be converted to acetyl CoA. Other sources of acetyl CoA include breakdown of free fatty acids and ketone bodies in the fasting state. Acetyl CoA can enter the citric acid cycle, a major source of reducing equivalents used to synthesize ATP, or enter biosynthetic pathways.

In addition to its role in energy generation, the citric acid cycle is a source of carbon skeletons for amino acid metabolism and other biosynthetic processes. One such process included here is the interconversion of 2-hydroxyglutarate, probably derived from porphyrin and amino acid metabolism, and 2-oxoglutarate (alpha-ketoglutarate), a citric acid cycle intermediate.

References

Edit history

Date	Action	Author
2003-11-03	Authored	Birney E, Schmidt EE, D'Eustachio P
2003-11-03	Created	Birney E, Schmidt EE, D'Eustachio P
2010-01-17	Revised	D'Eustachio P
2022-05-18	Edited	D'Eustachio P
2022-05-21	Modified	Weiser JD

5 submitted entities found in this pathway, mapping to 5 Reactome entities

Input	UniProt Id
IDH3A_HUMAN	P50213
ODPA_HUMAN	P08559

Input	UniProt Id
MAOX_HUMAN	P48163
SDHA_HUMAN	P31040

Input	UniProt Id
ODP2_HUMAN	P10515

3. Metabolism (R-HSA-1430728)

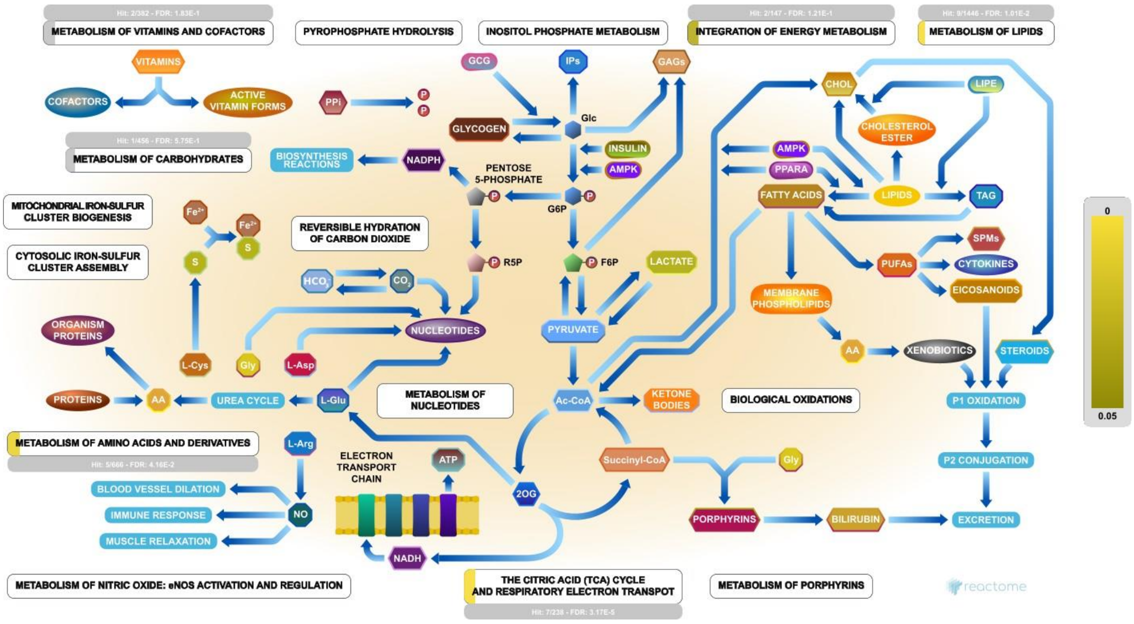

Metabolic processes in human cells generate energy through the oxidation of molecules consumed in the diet and mediate the synthesis of diverse essential molecules not taken in the diet as well as the inactivation and elimination of toxic ones generated endogenously or present in the extracellular environment. The processes of energy metabolism can be classified into two groups according to whether they involve carbohydrate-derived or lipid-derived molecules, and within each group it is useful to distinguish processes that mediate the breakdown and oxidation of these molecules to yield energy from ones that mediate their synthesis and storage as internal energy reserves. Synthetic reactions are conveniently grouped by the chemical nature of the end products, such as nucleotides, amino acids and related molecules, and porphyrins. Detoxification reactions (biological oxidations) are likewise conveniently classified by the chemical nature of the toxin.

At the same time, all of these processes are tightly integrated. Intermediates in reactions of energy generation are starting materials for biosyntheses of amino acids and other compounds, broad-specificity oxidoreductase enzymes can be involved in both detoxification reactions and biosyntheses, and hormone-mediated signaling processes function to coordinate the operation of energy-generating and energy-storing reactions and to couple these to other biosynthetic processes.

References

Edit history

Date	Action	Author
2011-07-07	Created	Jassal B
2022-05-21	Modified	Weiser JD

18 submitted entities found in this pathway, mapping to 18 Reactome entities

Input	UniProt Id	Input	UniProt Id	Input	UniProt Id
ACACA_HUMAN	Q13085	ACADM_HUMAN	P11310	ACLY_HUMAN	P53396
ATPB_HUMAN	P06576	CPT2_HUMAN	P23786	DECR_HUMAN	Q16698
IDH3A_HUMAN	P50213	MAOX_HUMAN	P48163	MECR_HUMAN	Q9BV79

Input	UniProt Id	Input	UniProt Id	Input	UniProt Id
MMSA_HUMAN	Q02252	ODP2_HUMAN	P10515	ODPA_HUMAN	P08559
PYC_HUMAN	P11498	QCR6_HUMAN	P07919	RAB14_HUMAN	P61106
RS12_HUMAN	P25398	SDHA_HUMAN	P31040	THIL_HUMAN	P24752

4. Fatty acid metabolism (R-HSA-8978868)

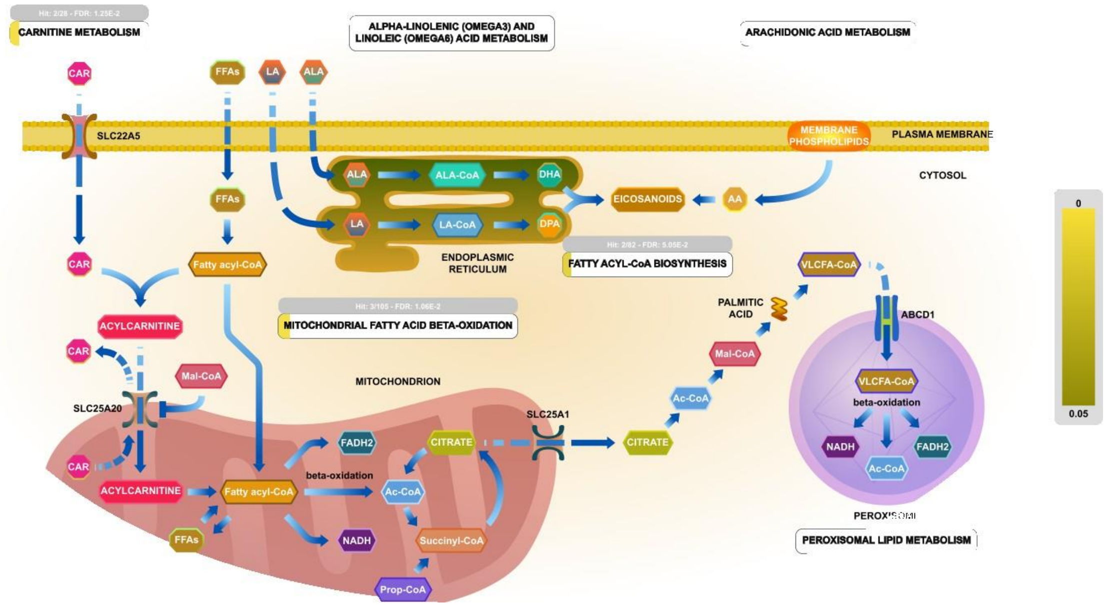

The synthesis and breakdown of fatty acids are a central part of human energy metabolism, and the eicosanoid class of fatty acid derivatives regulate diverse processes in the body (Vance & Vance 2008 - URL). Processes annotated in this module include the synthesis of fatty acids from acetyl-CoA, mitochondrial and peroxisomal breakdown of fatty acids, and the metabolism of eicosanoids and related molecules.

References

Biochemistry of Lipids, Lipoproteins and Membranes (Fifth Edition). Retrieved from http://www.sciencedirect.com/science/book/9780444532190

Edit history

Date	Action	Author
2007-02-03	Edited	Jassal B, Gopinathrao G, D'Eustachio P, Gillespie ME
2007-02-03	Reviewed	Jassal B, Gopinathrao G, D'Eustachio P, Gillespie ME
2007-02-03	Authored	Jassal B, Gopinathrao G, D'Eustachio P, Gillespie ME
2017-02-20	Revised	D'Eustachio P
2017-02-20	Created	D'Eustachio P
2022-05-21	Modified	Weiser JD

6 submitted entities found in this pathway, mapping to 6 Reactome entities

Input	UniProt Id	Input	UniProt Id	Input	UniProt Id
ACACA_HUMAN	Q13085	ACADM_HUMAN	P11310	ACLY_HUMAN	P53396
CPT2_HUMAN	P23786	DECR_HUMAN	Q16698	MECR_HUMAN	Q9BV79

5. ChREBP activates metabolic gene expression (R-HSA-163765)

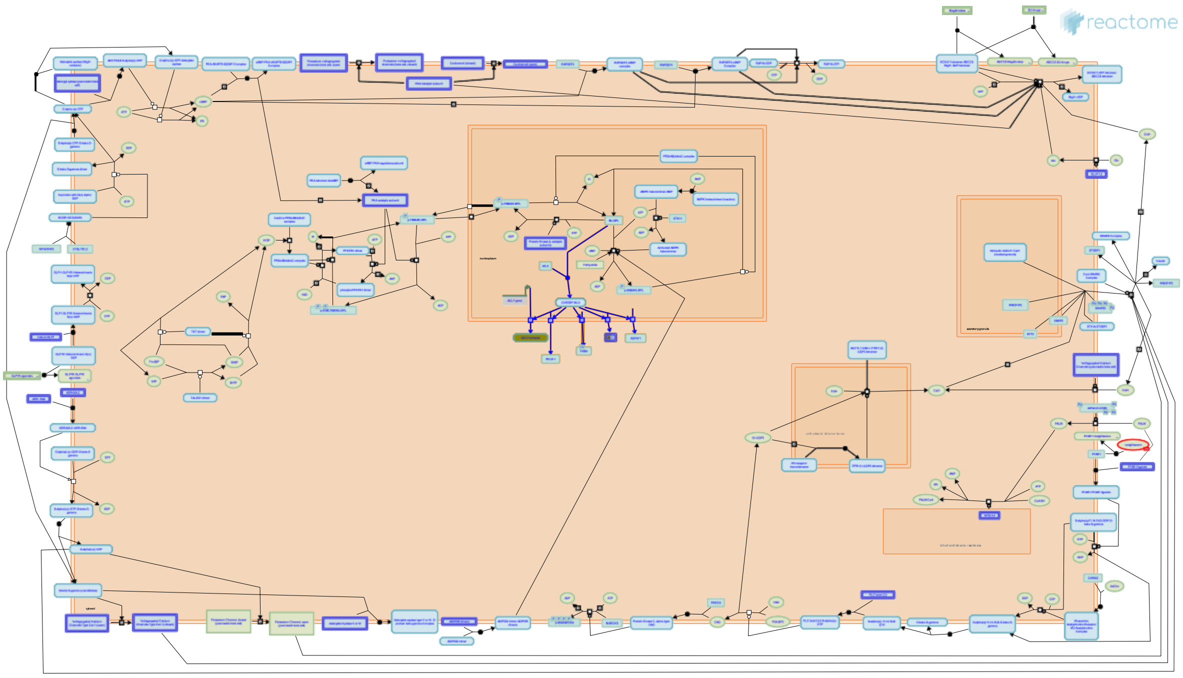

Cellular compartments: endoplasmic reticulum membrane, nucleoplasm, cytosol.

ChREBP (Carbohydrate Response Element Binding Protein) is a large multidomain protein containing a nuclear localization signal near its amino terminus, polyproline domains, a basic helix-loop-helix-leucine zipper domain, and a leucine-zipper-like domain (Uyeda et al., 2002). Its dephosphorylation in response to molecular signals associated with the well-fed state allows it to enter the nucleus, interact with MLX protein, and bind to ChRE DNA sequence motifs near Acetyl-CoA carboxylase, Fatty acid synthase, and Pyruvate kinase (L isoform) genes (Ishi et al.2004). This sequence of events is outlined schematically in the picture below (adapted from Kawaguchi et al. (2001) - copyright (2001) National Academy of Sciences, U.S.A.).

References

Uyeda K, Horton JD, Iizuka K, Liang G & Bruick RK (2004). Deficiency of carbohydrate response element-binding protein (ChREBP) reduces lipogenesis as well as glycolysis. Proc Natl Acad Sci U S A, 101, 7281-6.

Uyeda K, Kabashima T, Kawaguchi T & Takenoshita M (2001). Glucose and cAMP regulate the L-type pyruvate kinase gene by phosphorylation/dephosphorylation of the carbohydrate response element binding protein. Proc Natl Acad Sci U S A, 98, 13710-5.

Ma L, Tsatsos NG & Towle HC (2005). Direct role of ChREBP.Mlx in regulating hepatic glucose-responsive genes. J Biol Chem, 280, 12019-27.

Edit history

Date	Action	Author
2005-05-06	Created	Gopinathrao G
2005-05-13	Authored	Gopinathrao G
2022-05-21	Modified	Weiser JD

2 submitted entities found in this pathway, mapping to 2 Reactome entities

Input	UniProt Id	Input	UniProt Id
ACACA_HUMAN	Q13085	ACLY_HUMAN	P53396

6. Pyruvate metabolism (R-HSA-70268)

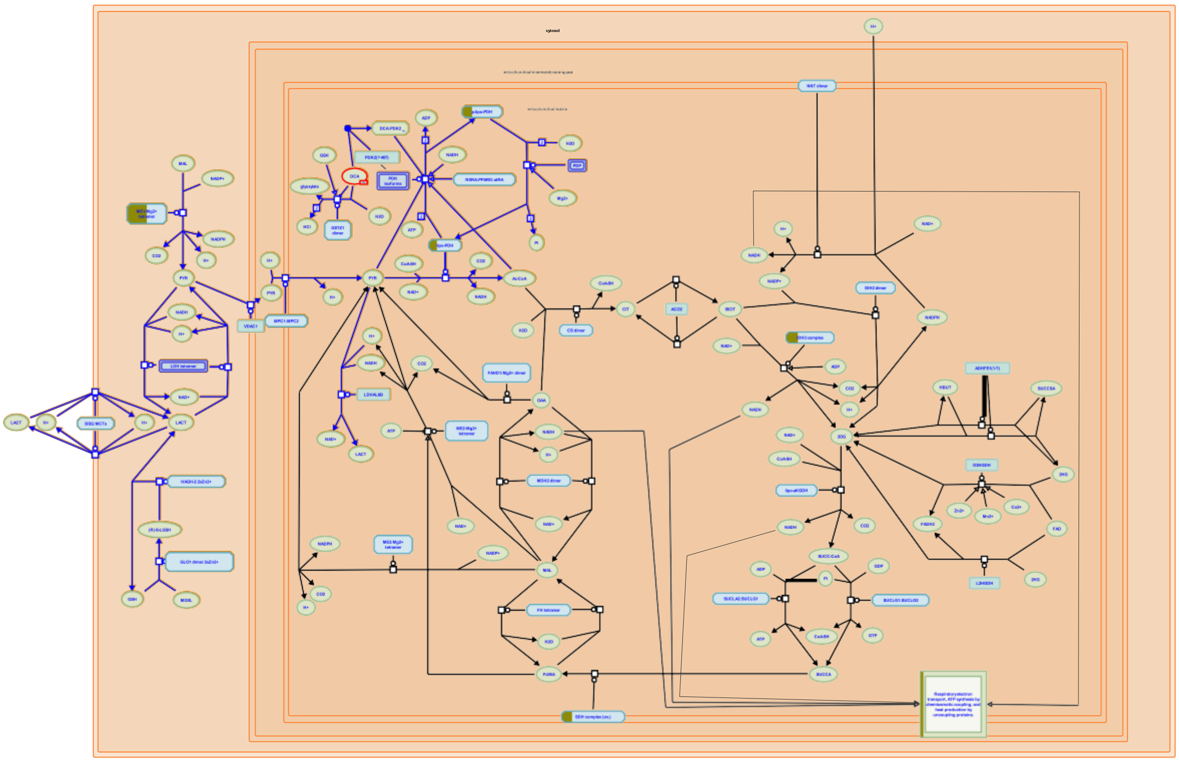

Pyruvate sits at an intersection of key pathways of energy metabolism. It is the end product of glycolysis and the starting point for gluconeogenesis, and can be generated by transamination of alanine. It can be converted by the pyruvate dehydrogenase complex to acetyl CoA (Reed and Hackert 1990) which can enter the TCA cycle or serve as the starting point for the syntheses of long chain fatty acids, steroids, and ketone bodies depending on the tissue and metabolic state in which it is formed. It also plays a central role in balancing the energy needs of various tissues in the body. Under conditions in which oxygen supply is limiting, e.g., in exercising muscle, or in the absence of mitochondria, e.g., in red blood cells, re-oxidation of NADH produced by glycolysis cannot be coupled to generation of ATP. Instead, re-oxidation is coupled to the reduction of pyruvate to lactate. This lactate is released into the blood, and is taken up primarily by the liver, where it is oxidized to pyruvate and can be used for gluconeogenesis (Cori 1981).

References

Reed LJ & Hackert ML (1990). Structure-function relationships in dihydrolipoamide acyltransferases. J Biol Chem, 265, 8971-4.

Cori CF (1981). The glucose-lactic acid cycle and gluconeogenesis. Curr Top Cell Regul, 18, 377-87.

Edit history

Date	Action	Author
2009-12-18	Revised	D'Eustachio P
2022-05-21	Modified	Weiser JD

3 submitted entities found in this pathway, mapping to 3 Reactome entities

Input	UniProt Id	Input	UniProt Id	Input	UniProt Id
MAOX_HUMAN	P48163	ODP2_HUMAN	P10515	ODPA_HUMAN	P08559

7. Defective HLCS causes multiple carboxylase deficiency (R-HSA-3371599)

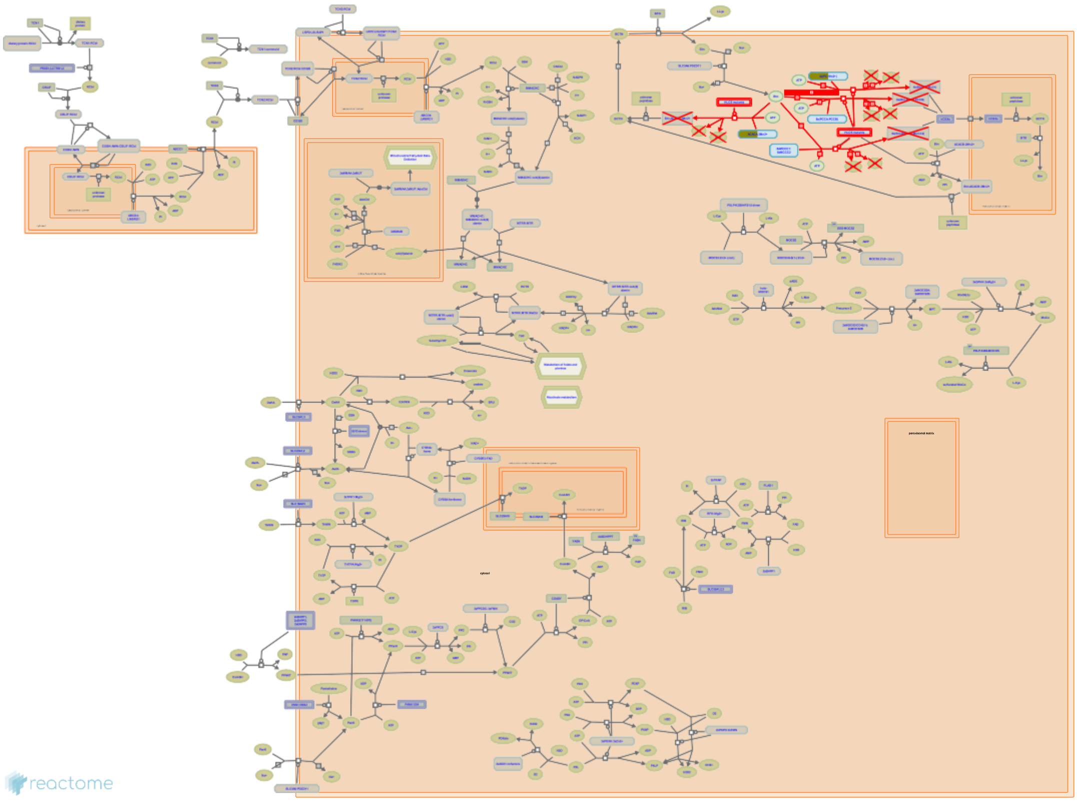

Diseases: vitamin metabolic disorder.

Defects in HLCS causes holocarboxylase synthetase deficiency (HLCS deficiency aka early onset multiple carboxylase deficiency; MIM:253270). HLCS deficiency is an autosomal recessive disorder whereby deficient HLCS activity results in reduced activity of all five biotin-dependent carboxylases. Symptoms include metabolic acidosis, organic aciduria, lethargy, hypotonia, convulsions and dermatitis (Suzuki et al. 2005). Patients can present symptoms shortly after birth to up to early childhood and will be prescribed oral biotin supplements, typically 10-20 mg daily. Two classes of HLCS deficiency have been reported depending on whether patients respond to biotin therapy. Most patients respond favourably to treatment and show complete reversal of biochemical and clinical symptoms (Morrone et al. 2002, Dupuis et al. 1999). Here mutations in the HLCS active site cause a reduced affinity for biotin that can be overcome by pharmacological doses of the vitamin (Pendini et al. 2008). Patients who display incomplete responsiveness to biotin therapy have a poor long-term prognosis (Bailey et al. 2008). Here mutations that reside outside of the enzyme's active site have no effect on biotin binding but do compromise the protein-protein interaction between the HLCS and its substrates, resulting in reduced biotinylation of all five carboxylases thus reducing their enzymatic activity (Mayende et al. 2012).

References

Jitrapakdee S, Wilson CJ, Bailey LM, Wallace JC, Polyak SW & Ivanov RA (2008). Reduced half-life of holocarboxylase synthetase from patients with severe multiple carboxylase deficiency. Hum. Mutat., 29, E47-57.

Mayende L, Booker GW, Bailey LM, Wallace JC, Swift RD, Polyak SW & Soares da Costa TP (2012). A novel molecular mechanism to explain biotin-unresponsive holocarboxylase synthetase deficiency. J. Mol. Med., 90, 81-8.

Suzuki Y, Aoki Y, Kure S, Yang X & Matsubara Y (2005). Mutations in the holocarboxylase synthetase gene HLCS. Hum. Mutat., 26, 285-90.

Pendini NR, Booker GW, Bailey LM, Wallace JC, Polyak SW & Wilce MC (2008). Microbial biotin protein ligases aid in understanding holocarboxylase synthetase deficiency. Biochim. Biophys. Acta, 1784, 973-82.

Pela I, Boneh A, Zammarchi E, Morrone A, Funghini S, Pasquini E, ... Malvagia S (2002). Clinical findings and biochemical and molecular analysis of four patients with holocarboxylase synthetase deficiency. Am. J. Med. Genet., 111, 10-8.

Edit history

Date	Action	Author
2013-05-13	Edited	Jassal B
2013-05-13	Authored	Jassal B
2013-05-13	Created	Jassal B
2013-08-15	Reviewed	Polyak SW
2018-01-26	Modified	Jassal B

2 submitted entities found in this pathway, mapping to 2 Reactome entities

Input	UniProt Id	Input	UniProt Id
ACACA_HUMAN	Q13085	PYC_HUMAN	P11498

8. Defects in biotin (Btn) metabolism (R-HSA-3323169)

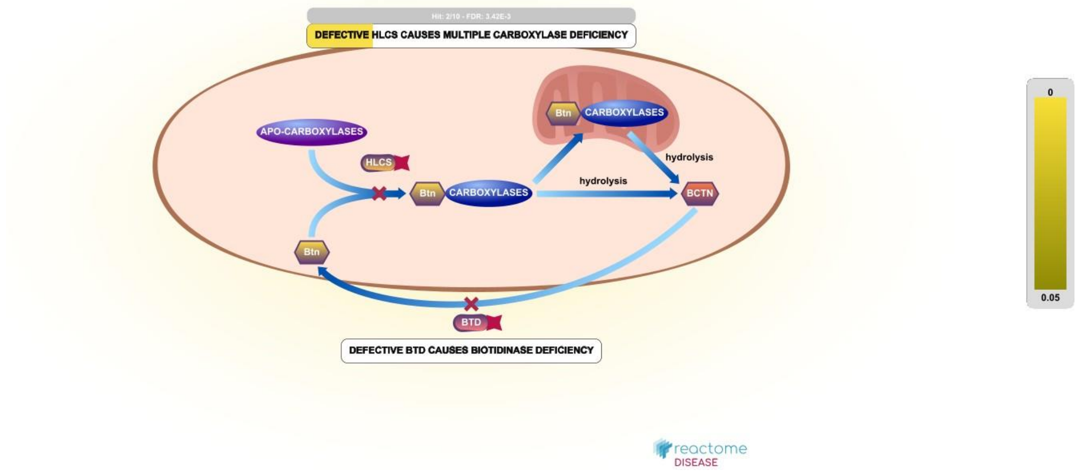

Diseases: vitamin metabolic disorder.

Biotin (Btn, vitamin B7, vitamin H, coenzyme R) is an essential cofactor for five biotin-dependent carboxylase enzymes, involved in the synthesis of fatty acids, isoleucine, valine and in gluconeogenesis. Thus, Btn is necessary for cell growth, fatty acid synthesis and the metabolism of fats and amino acids. Inherited metabolic disorders characterized by deficient activities of all five biotin dependent carboxylases are termed multiple carboxylase deficiencies. Two congenital defects in biotin metabolism leading to multiple carboxylase deficiency are known, holocarboxylase synthetase deficiency (MIM 609018) and biotinidase deficiency (MIM 253260). In both scenarios symptoms include ketolactic acidosis, organic aciduria, hyperammonemia, skin rashes, hypotonia, seizures, developmental delay, alopecia, and coma. As humans are auxotrophic for Btn, the micronutrient must be obtained from external soures such as intestinal microflora and dietary forms. Accordingly, severe malnutrition can also give rise to biotin deficiency and multiple carboxylase deficiency. Biotin deficiency can also be induced by the excessive consumption of raw egg white that contains the biotin-binding protein avidin. Holocarboxylase synthetase deficiency arises when all five biotin-dependent enzymes are not biotinylated leading to their reduced activities. The defective genes causing these conditions are described here (Pendini et al. 2008, Suzuki et al. 2005). Biotinidase deficiency is caused by defects in the recycling of Btn. General symptoms include decreased appetite and growth, dermatitis and perosis. The defective genes causing these conditions are described here (Procter et al. 2013).

References

Wolf B, Crockett DK, Procter M & Mao R (2013). The Biotinidase Gene Variants Registry: A Paradigm Public Database. G3 (Bethesda).

Suzuki Y, Aoki Y, Kure S, Yang X & Matsubara Y (2005). Mutations in the holocarboxylase synthetase gene HLCS. Hum. Mutat., 26, 285-90.

Pendini NR, Booker GW, Bailey LM, Wallace JC, Polyak SW & Wilce MC (2008). Microbial biotin protein ligases aid in understanding holocarboxylase synthetase deficiency. Biochim. Biophys. Acta, 1784, 973-82.

Edit history

Date	Action	Author
2013-05-09	Edited	Jassal B
2013-05-09	Authored	Jassal B
2013-05-09	Created	Jassal B
2013-08-15	Reviewed	Polyak SW
2021-08-16	Modified	Matthews L

2 submitted entities found in this pathway, mapping to 2 Reactome entities

Input	UniProt Id	Input	UniProt Id
ACACA_HUMAN	Q13085	PYC_HUMAN	P11498

9. Biotin transport and metabolism (R-HSA-196780)

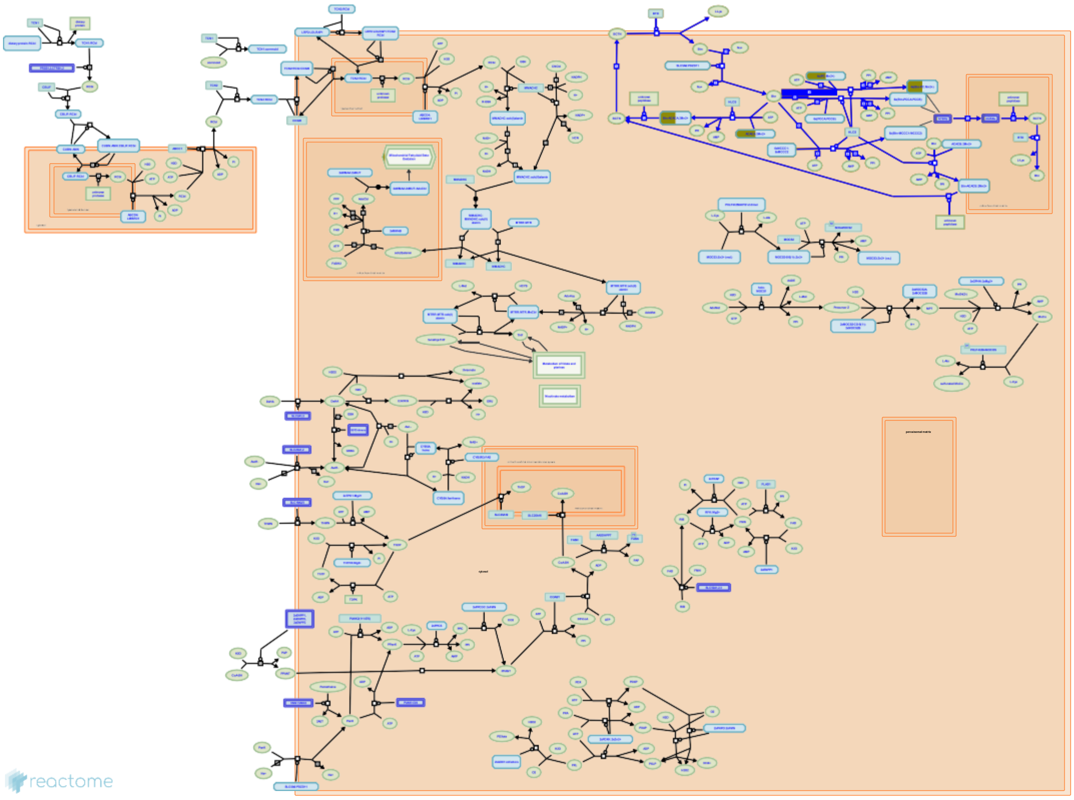

Biotin (Btn) is an essential cofactor in a variety of carboxylation reactions (Zempleni et al. 2009). Humans cannot synthesize Btn but it is abundant in the human diet and can be taken up from the intestinal lumen by the SLC5A6 transporter. Its uptake, intracellular translocation, covalent conjugation to apoenzymes, and salvage are described here.

References

Wijeratne SS, Hassan YI & Zempleni J (2009). Biotin. Biofactors, 35, 36-46.

Edit history

Date	Action	Author
2007-04-24	Edited	Jassal B
2007-04-24	Authored	Jassal B
2007-04-24	Created	Jassal B
2013-02-07	Revised	Jassal B
2014-04-07	Revised	Jassal B
2022-05-21	Modified	Weiser JD

2 submitted entities found in this pathway, mapping to 2 Reactome entities

Input	UniProt Id	Input	UniProt Id
ACACA_HUMAN	Q13085	PYC_HUMAN	P11498

10. Beta oxidation of decanoyl-CoA to octanoyl-CoA-CoA (R-HSA-77346)

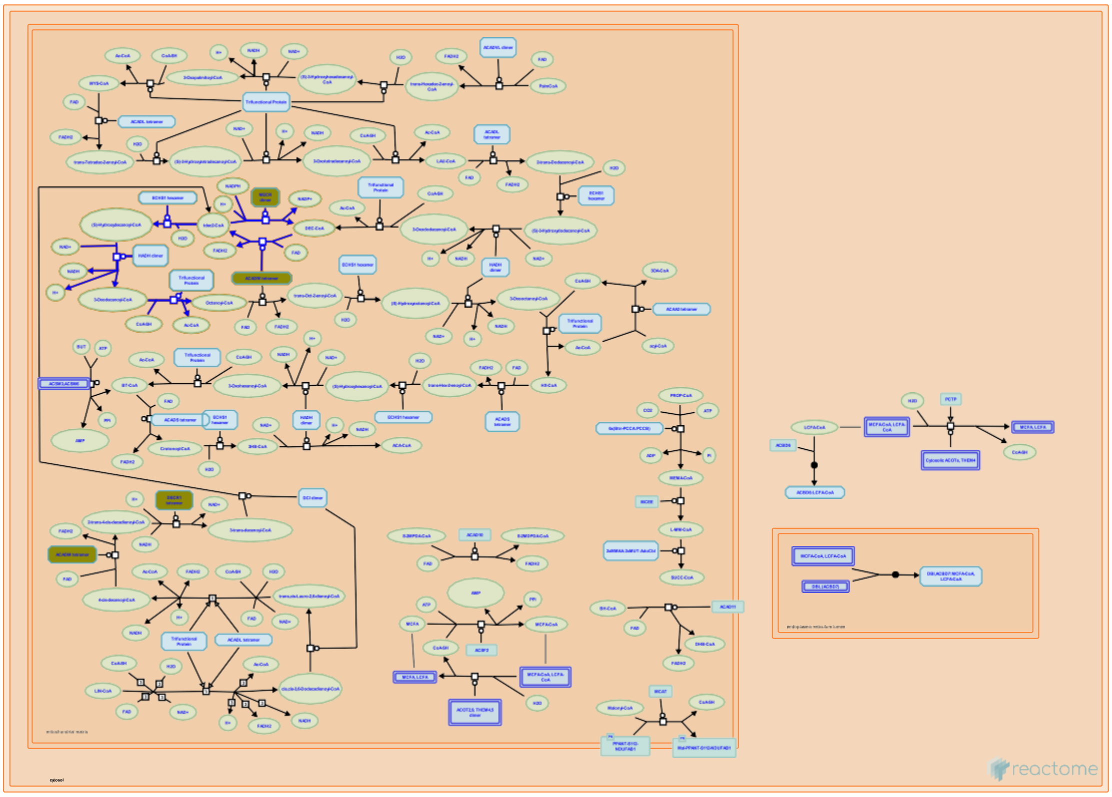

Cellular compartments: mitochondrial matrix.

The fourth pass through the beta-oxidation spiral picks up where the last left off with the saturated fatty acid decanoyl-CoA and produces octanoyl-CoA. Four enzymatic steps are required starting with MCAD CoA dehydrogenase (Medium Chain) activity, followed by the enoyl-CoA hydratase activity of crotonase, the 3-hydroxyacyl-CoA dehydrogenase activity of the short chain 3-hydroxy-acyl-CoA dehydrogenase (SCHAD), and completed by the ketoacyl-CoA thiolase activity, present in the mitochondrial membrane associated trifunctional protein. Note that the 3-hydroxyacyl-CoA dehydrogenase activity of SCHAD is not actually limited to short chain fatty acids, in fact SCHAD has a broad substrate specificity.

References

Beaudet AL, Scriver CR, Sly WS & Valle D (2001). *Mitochondrial fatty acid oxidation disorders, The Metabolic and Molecular Bases of Inherited Disease, 8th ed* , 2297-2326.

Edit history

Date	Action	Author
2003-09-19	Authored	Gillespie ME
2003-09-19	Created	Gillespie ME
2022-05-18	Edited	Gillespie ME
2022-05-20	Modified	Weiser JD

2 submitted entities found in this pathway, mapping to 2 Reactome entities

Input	UniProt Id	Input	UniProt Id
ACADM_HUMAN	P11310	MECR_HUMAN	Q9BV79

11. mitochondrial fatty acid beta-oxidation of unsaturated fatty acids (R-HSA-77288)

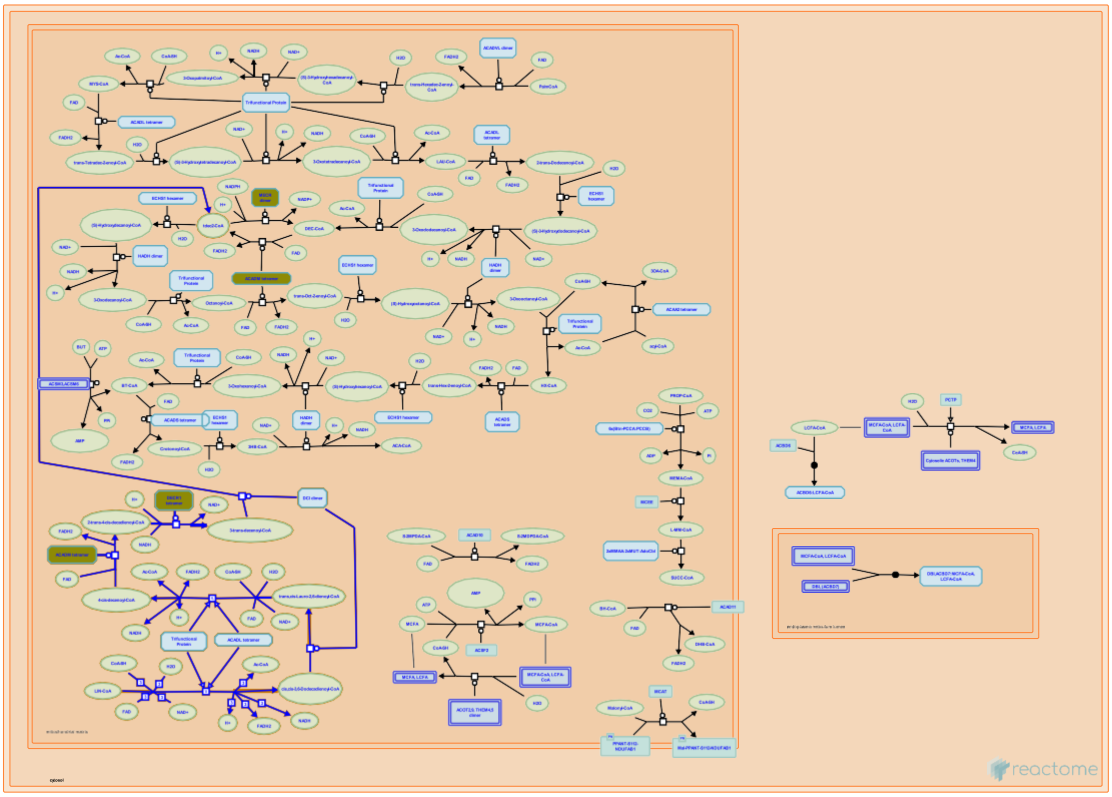

Cellular compartments: mitochondrial matrix.

The complete beta-oxidation spiral produces and consumes intermediates with a trans configuration. Mitochondrial beta-oxidation of unsaturated fatty acids leads to intermediates not compatible with the four enzymatic steps responsible for the beta-oxidation of saturated fatty acids. Unsaturated fatty acids that have bonds in the cis configuration require three separate enzymatic steps to prepare these molecules for the beta-oxidation pathway. The further processing of these intermediates requires additional enzymes, depending on the position of the double bonds in the original fatty acids. Described here is the beta-oxidation of linoleoyl-CoA.

References

Beaudet AL, Scriver CR, Sly WS & Valle D (2001). *Mitochondrial fatty acid oxidation disorders, The Metabolic and Molecular Bases of Inherited Disease, 8th ed* , 2297-2326.

Edit history

Date	Action	Author
2003-09-19	Created	Gillespie ME
2003-10-25	Authored	Gillespie ME
2022-05-18	Edited	Gillespie ME
2022-05-20	Modified	Weiser JD

2 submitted entities found in this pathway, mapping to 2 Reactome entities

Input	UniProt Id	Input	UniProt Id
ACADM_HUMAN	P11310	DECR_HUMAN	Q16698

12. Metabolism of lipids (R-HSA-556833)

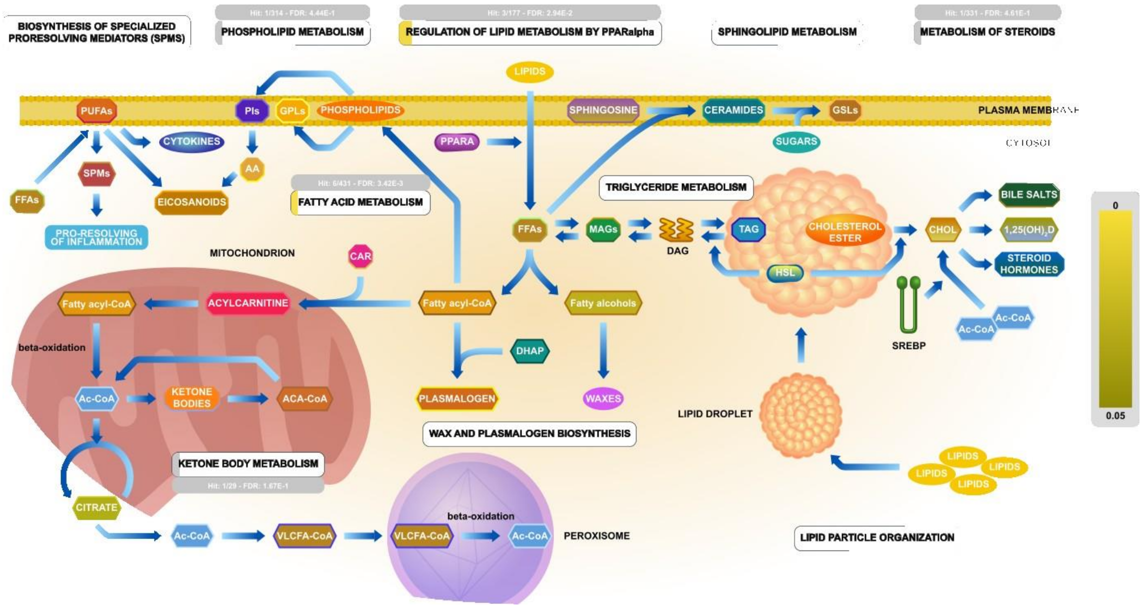

Lipids are hydrophobic but otherwise chemically diverse molecules that play a wide variety of roles in human biology. They include ketone bodies, fatty acids, triacylglycerols, phospholipids and sphingolipids, eicosanoids, cholesterol, bile salts, steroid hormones, and fat-soluble vitamins. They function as a major source of energy (fatty acids, triacylglycerols, and ketone bodies), are major constituents of cell membranes (cholesterol and phospholipids), play a major role in their own digestion and uptake (bile salts), and participate in numerous signaling and regulatory processes (steroid hormones, eicosanoids, phosphatidylinositols, and sphingolipids) (Vance & Vance 2008 - URL).

The central steroid in human biology is cholesterol, obtained from animal fats consumed in the diet or synthesized de novo from acetyl-coenzyme A. (Vegetable fats contain various sterols but no cholesterol.) Cholesterol is an essential constituent of lipid bilayer membranes and is the starting point for the biosyntheses of bile acids and salts, steroid hormones, and vitamin D. Bile acids and salts are mostly synthesized in the liver. They are released into the intestine and function as detergents to solubilize dietary fats. Steroid hormones are mostly synthesized in the adrenal gland and gonads. They regulate energy metabolism and stress responses (glucocorticoids), salt balance (mineralocorticoids), and sexual development and function (androgens and estrogens). At the same time, chronically elevated cholesterol levels in the body are associated with the formation of atherosclerotic lesions and hence increased risk of heart attacks and strokes. The human body lacks a mechanism for degrading excess cholesterol, although an appreciable amount is lost daily in the form of bile salts and acids that escape recycling.

Aspects of lipid metabolism currently annotated in Reactome include lipid digestion, mobilization, and transport; fatty acid, triacylglycerol, and ketone body metabolism; peroxisomal lipid metabolism; phospholipid and sphingolipid metabolism; cholesterol biosynthesis; bile acid and bile salt metabolism; and steroid hormone biosynthesis.

References

Biochemistry of Lipids, Lipoproteins and Membranes (Fifth Edition). Retrieved from http://www.sciencedirect.com/science/book/9780444532190

Edit history

Date	Action	Author
2007-02-03	Authored	Jassal B, Gopinathrao G, D'Eustachio P, Gillespie ME
2010-03-23	Created	D'Eustachio P
2017-02-21	Revised	D'Eustachio P
2022-05-18	Edited	Joshi-Tope G, D'Eustachio P
2022-05-21	Modified	Weiser JD

9 submitted entities found in this pathway, mapping to 9 Reactome entities

Input	UniProt Id	Input	UniProt Id	Input	UniProt Id
ACACA_HUMAN	Q13085	ACADM_HUMAN	P11310	ACLY_HUMAN	P53396
CPT2_HUMAN	P23786	DECR_HUMAN	Q16698	MAOX_HUMAN	P48163
MECR_HUMAN	Q9BV79	RAB14_HUMAN	P61106	THIL_HUMAN	P24752

13. Mitochondrial Fatty Acid Beta-Oxidation (R-HSA-77289)

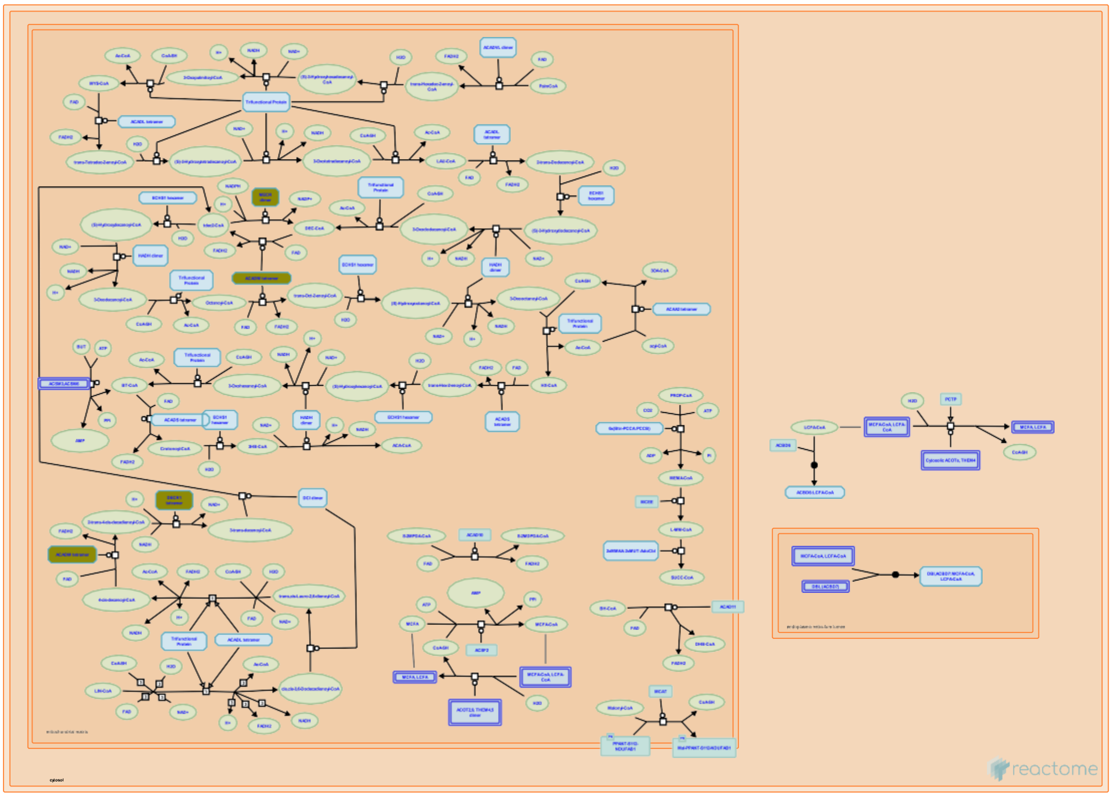

Cellular compartments: mitochondrial matrix.

Beta-oxidation begins once fatty acids have been imported into the mitochondrial matrix by carnitine acyltransferases. The beta-oxidation spiral of fatty acids metabolism involves the repetitive removal of two carbon units from the fatty acyl chain. There are four steps to this process: oxidation, hydration, a second oxidation, and finally thiolysis. The last step releases the two-carbon acetyl-CoA and a ready primed acyl-CoA that takes another turn down the spiral. In total each turn of the beta-oxidation spiral produces one NADH, one FADH2, and one acetyl-CoA.

Further oxidation of acetyl-CoA via the tricarboxylic acid cycle generates additional FADH2 and NADH. All reduced cofactors are used by the mitochondrial electron transport chain to form ATP. The complete oxidation of a fatty acid molecule produces numerous ATP molecules. Palmitate, used as the model here, produces 129 ATPs.

Beta-oxidation pathways differ for saturated and unsaturated fatty acids. The beta-oxidation of saturated fatty acids requires four different enzymatic steps. Beta-oxidation produces and consumes intermediates with a trans configuration; unsaturated fatty acids that have bonds in the cis configuration require three separate enzymatic steps to prepare these molecules for the beta-oxidation pathway.

References

Coates PM & Tanaka K (1992). Molecular basis of mitochondrial fatty acid oxidation defects. J Lipid Res, 33, 1099-110.

Rinaldo P, Bennett MJ & Matern D (2002). Fatty acid oxidation disorders. Annu Rev Physiol, 64, 477-502.

Beaudet AL, Scriver CR, Sly WS & Valle D (2001). *Mitochondrial fatty acid oxidation disorders, The Metabolic and Molecular Bases of Inherited Disease, 8th ed* , 2297-2326.

Roe CR & Roe DS (2000). Recent developments in the investigation of inherited metabolic disorders using cultured human cells. Mol Genet Metab, 68, 243-57.

Hale DE & Stanley CA (1994). Genetic disorders of mitochondrial fatty acid oxidation. Curr Opin Pediatr, 6, 476-81.

Edit history

Date	Action	Author
2003-09-19	Authored	Gillespie ME
2003-09-19	Created	Gillespie ME
2022-05-18	Edited	Gillespie ME
2022-05-21	Modified	Weiser JD

3 submitted entities found in this pathway, mapping to 3 Reactome entities

Input	UniProt Id	Input	UniProt Id	Input	UniProt Id
ACADM_HUMAN	P11310	DECR_HUMAN	Q16698	MECR_HUMAN	Q9BV79

14. Carnitine metabolism (R-HSA-200425)

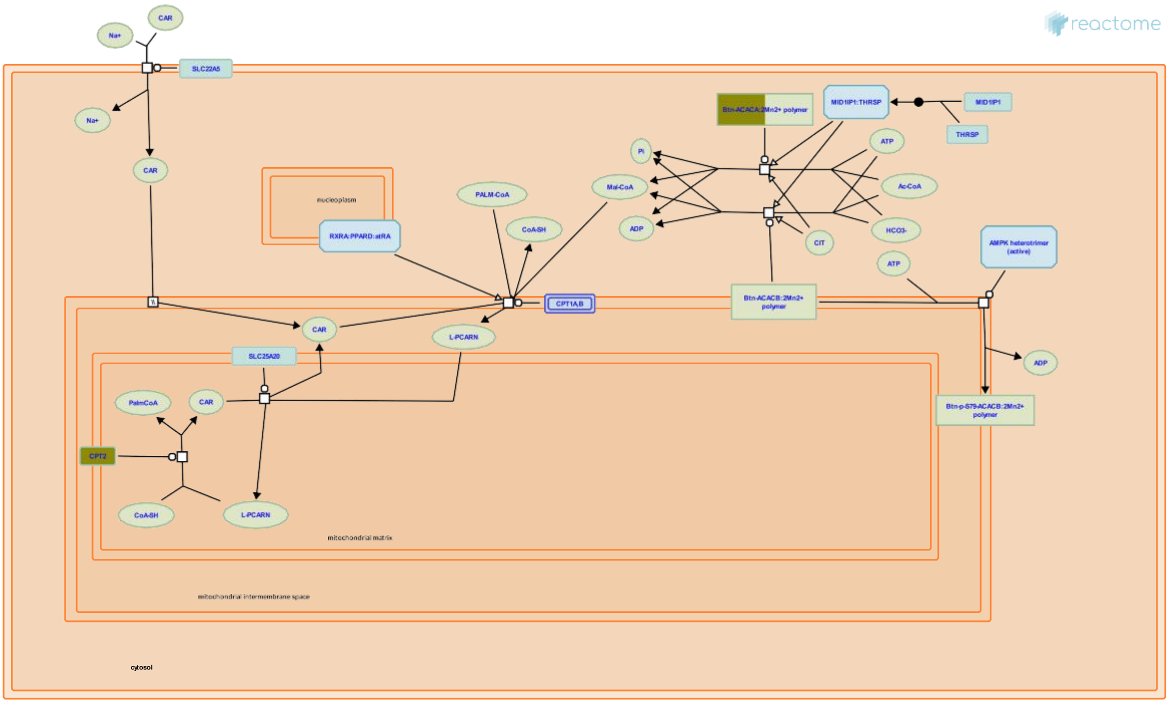

Cellular compartments: mitochondrion, cytosol.

The mitochondrial carnitine system catalyzes the transport of long-chain fatty acids into the mitochondrial matrix where they undergo beta oxidation. This transport system consists of the malonyl-CoA sensitive carnitine palmitoyltransferase I (CPT-I) localized in the mitochondrial outer membrane, the carnitine:acylcarnitine translocase, an integral inner membrane protein, and carnitine palmitoyltransferase II localized on the matrix side of the inner membrane. (Kerner and Hoppel, 2000).

References

Gandour RD, Ramsay RR & van der Leij FR (2001). Molecular enzymology of carnitine transfer and transport. Biochim Biophys Acta, 1546, 21-43.

Edit history

Date	Action	Author
2007-07-18	Created	Gopinathrao G
2007-07-30	Edited	Gopinathrao G
2007-07-30	Authored	Gopinathrao G
2007-07-31	Reviewed	D'Eustachio P
2022-05-21	Modified	Weiser JD

2 submitted entities found in this pathway, mapping to 2 Reactome entities

Input	UniProt Id	Input	UniProt Id
ACACA_HUMAN	Q13085	CPT2_HUMAN	P23786

15. Regulation of pyruvate dehydrogenase (PDH) complex (R-HSA-204174)

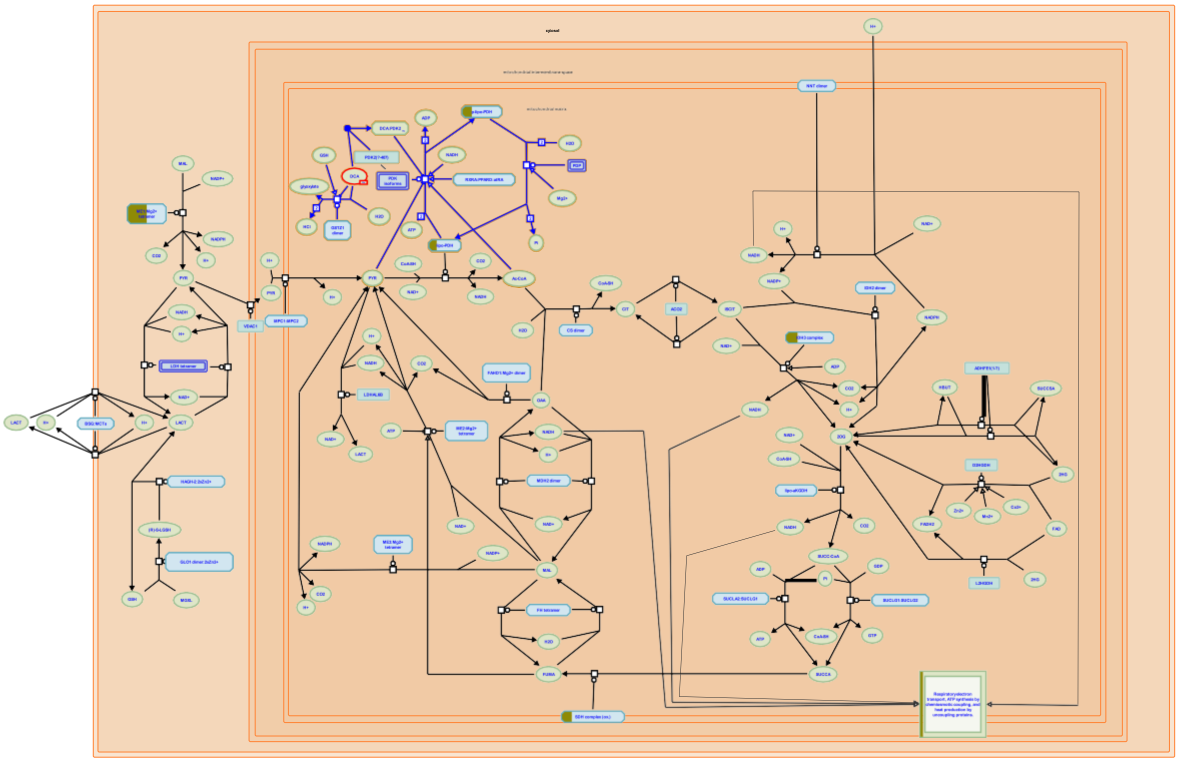

Cellular compartments: mitochondrial matrix.

The mitochondrial pyruvate dehydrogenase (PDH) complex catalyzes the oxidative decarboxylation of pyruvate, linking glycolysis to the tricarboxylic acid cycle and fatty acid synthesis. PDH inactivation is crucial for glucose conservation when glucose is scarce, while adequate PDH activity is required to allow both ATP and fatty acid production from glucose. The mechanisms that control human PDH activity include its phosphorylation (inactivation) by pyruvate dehydrogenase kinases (PDK 1-4) and its dephosphorylation (activation, reactivation) by pyruvate dehydrogenase phosphate phosphatases (PDP 1 and 2). Isoform-specific differences in kinetic parameters, regulation, and phosphorylation site specificity of the PDKs introduce variations in the regulation of PDC activity in differing endocrine and metabolic states (Sugden and Holness 2003).

References

Holness MJ & Sugden MC (2003). Recent advances in mechanisms regulating glucose oxidation at the level of the pyruvate dehydrogenase complex by PDKs. Am J Physiol Endocrinol Metab, 284, E855-62.

Edit history

Date	Action	Author
2007-11-27	Authored	Gopinathrao G
2007-11-27	Created	Gopinathrao G
2008-01-12	Reviewed	D'Eustachio P
2009-12-18	Revised	D'Eustachio P
2022-05-20	Modified	Weiser JD

2 submitted entities found in this pathway, mapping to 2 Reactome entities

Input	UniProt Id	Input	UniProt Id
ODP2_HUMAN	P10515	ODPA_HUMAN	P08559

16. Defects in vitamin and cofactor metabolism (R-HSA-3296482)

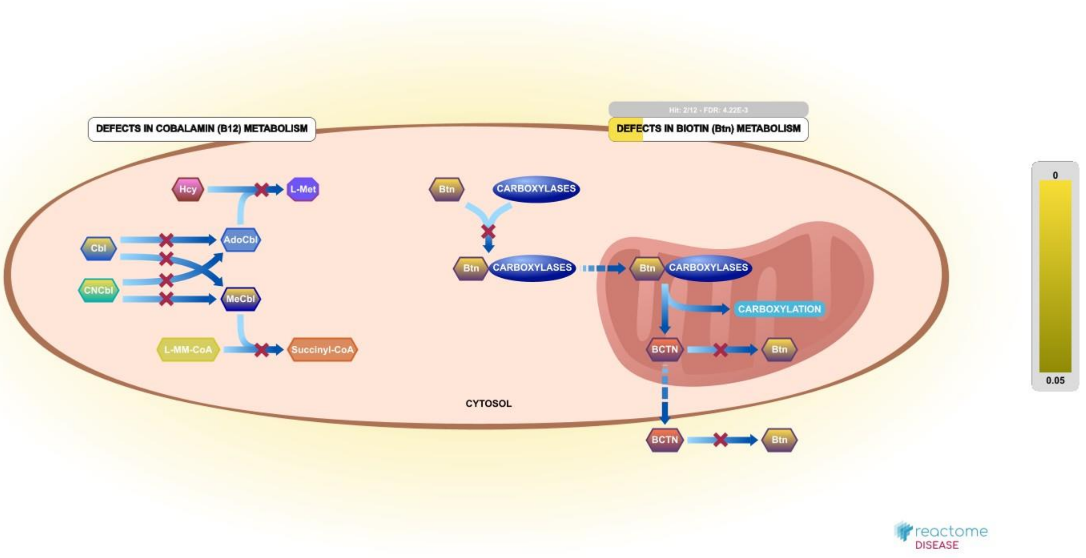

Diseases: vitamin metabolic disorder.

Vitamins are essential nutrients, required in small amounts from the diet for the normal growth and development of a multicellular organism. Where there is vitamin deficiency, either by poor diet or a defect in metabolic conversion, diseases called Avitaminoses occur. Currently, cobalamin (Cbl, vitamin B12) metabolic defects are described below (Chapter 155 in The Metabolic and Molecular Bases of Inherited Disease, 8th ed, Scriver et al. 2001)

References

Beaudet AL, Antonarakis SE, Ballabio A, Kinzler KW, Valle D & Vogelstein B (2001). *Chapter 155: Inherited Disorders of Folate and Cobalamin Transport and Metabolism, The Online Metabolic and Molecular Bases of Inherited Disease* .

Wijeratne SS, Hassan YI & Zempleni J (2008). Biotin and biotinidase deficiency. Expert Rev Endocrinol Metab, 3, 715-724.

Edit history

Date	Action	Author
2013-04-18	Edited	Jassal B
2013-04-18	Authored	Jassal B
2013-04-18	Created	Jassal B
2013-08-14	Reviewed	Watkins D
2020-05-28	Modified	Jassal B

2 submitted entities found in this pathway, mapping to 2 Reactome entities

Input	UniProt Id	Input	UniProt Id
ACACA_HUMAN	Q13085	PYC_HUMAN	P11498

17. Respiratory electron transport, ATP synthesis by chemiosmotic coupling, and heat production by uncoupling proteins. (R-HSA-163200)

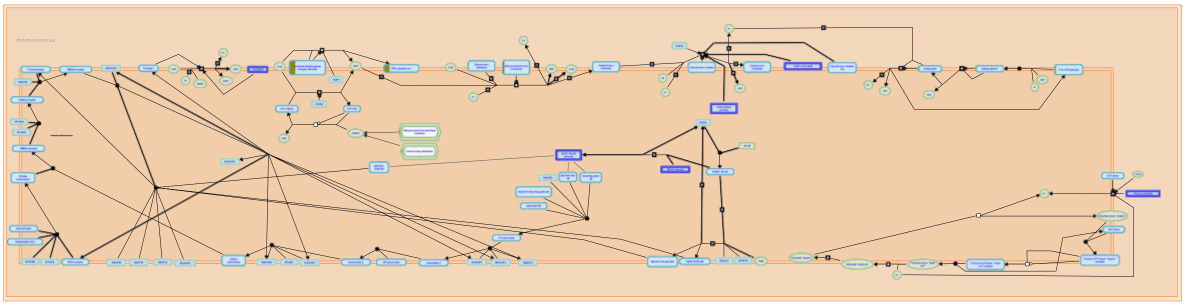

Oxidation of fatty acids and pyruvate in the mitochondrial matrix yield large amounts of NADH. The respiratory electron transport chain couples the re-oxidation of this NADH to NAD+ to the export of protons from the mitochonrial matrix, generating a chemiosmotic gradient across the inner mitochondrial membrane. This gradient is used to drive the synthesis of ATP; it can also be by-passed by uncoupling proteins to generate heat, a reaction in brown fat that may be important in regulation of body temperature in newborn children.

References

Edit history

Date	Action	Author
2005-04-21	Authored	Jassal B
2005-04-21	Created	Jassal B
2005-05-12	Reviewed	Ferguson SJ
2022-05-20	Modified	Weiser JD

3 submitted entities found in this pathway, mapping to 3 Reactome entities

Input	UniProt Id	Input	UniProt Id	Input	UniProt Id
ATPB_HUMAN	P06576	QCR6_HUMAN	P07919	SDHA_HUMAN	P31040

18. Citric acid cycle (TCA cycle) (R-HSA-71403)

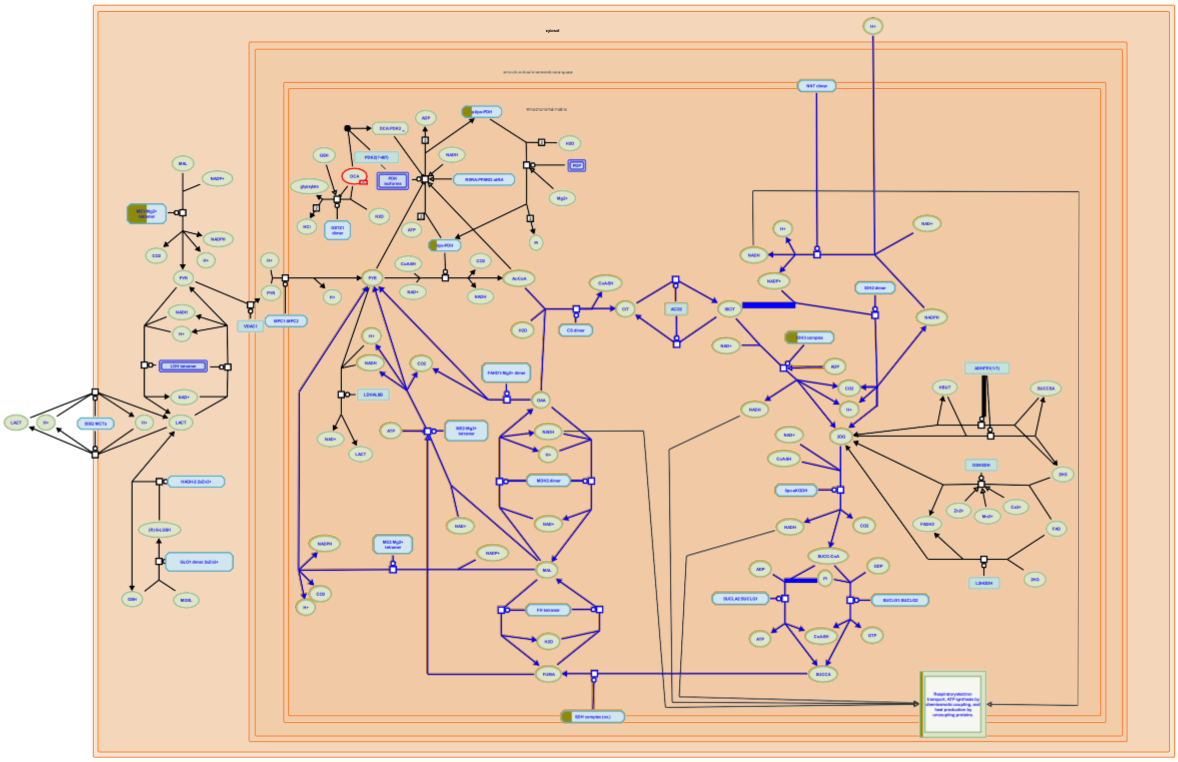

Cellular compartments: mitochondrion.

In the citric acid or tricarboxylic acid (TCA) cycle, the acetyl group of acetyl CoA (derived primarily from oxidative decarboxylation of pyruvate, beta-oxidation of long-chain fatty acids, and catabolism of ketone bodies and several amino acids) can be completely oxidized to CO2 in reactions that also yield one high-energy phosphate bond (as GTP or ATP) and four reducing equivalents (three NADH + H+, and one FADH2). The NADH and FADH2 are then oxidized by the electron transport chain to yield nine more high-energy phosphate bonds (as ATP). All reactions of the citric acid cycle take place in the mitochondrion.

Eight canonical reactions mediate the synthesis of citrate from acetyl-CoA and oxaloacetate and the metabolism of citrate to re-form oxaloacetate. Six additional reactions are included here. Three reversible reactions, the interconversions of citrate and isocitrate, of fumarate and malate, and of malate and oxaloacetate are annotated in both their canonical (forward) and reverse directions. The synthesis of succinate from succinyl-CoA can be coupled to the phosphorylation of either GDP (the canonical reaction) or ADP; both reactions are annotated. Two mitochondrial isocitrate dehydrogenase isozymes catalyze the oxidative decarboxylation of isocitrate to form alpha-ketoglutarate (2-oxoglutarate): IDH3 catalyzes the canonical reaction coupled to the reduction of NAD+, while IDH2 catalyzes the same reaction coupled to reduction of NADP+, a reaction whose normal physiological function is unclear. Both reactions are annotated. Finally, a reaction is annotated in which reducing equivalents are transferred from NADPH to NAD+ coupled to proton import across the inner mitochondrial membrane.

The cyclical nature of the reactions responsible for the oxidation of acetate was first suggested by Hans Krebs, from biochemical studies of pigeon breast muscle (Krebs et al. 1938; Krebs and Eggleston 1940). Many of the molecular details of individual reactions were worked out by Ochoa and colleagues, largely through studies of enzymes purified from pig heart (Ochoa 1980). While the human homologues of these enzymes have all been identified, their biochemical characterization has in general been limited and many molecular details of the human reactions are inferred from those worked out in studies of the model systems.

References

Ochoa S (1980). The pursuit of a hobby. Annu Rev Biochem, 49, 1-30.

Eggleston LV & Krebs HA (1940). The oxidation of pyruvate in pigeon breast muscle. Biochem J, 34, 442-459.

Krebs HA, Johnson WA & Salvin E (1938). The formation of citric and alpha-ketoglutaric acids in the mammalian body. Biochem J, 32, 113-117.

Edit history

Date	Action	Author
2003-01-28	Authored	Birney E
2009-12-26	Revised	D'Eustachio P
2022-05-18	Edited	D'Eustachio P
2022-06-07	Modified	Weiser JD

2 submitted entities found in this pathway, mapping to 2 Reactome entities

Input	UniProt Id	Input	UniProt Id
IDH3A_HUMAN	P50213	SDHA_HUMAN	P31040

19. PPARA activates gene expression (R-HSA-1989781)

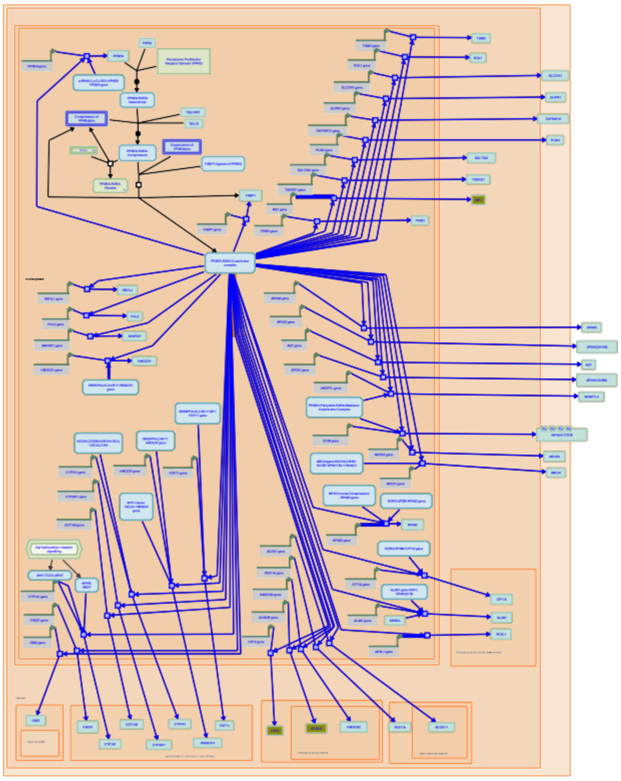

Cellular compartments: peroxisomal matrix, endoplasmic reticulum membrane, plasma membrane, nucleoplasm, mitochondrial outer membrane, cytosol, mitochondrial matrix, extracellular region, lipid droplet, mitochondrial inner membrane, peroxisomal membrane.

The set of genes regulated by PPAR-alpha is not fully known in humans, however many examples have been found in mice. Genes directly activated by PPAR-alpha contain peroxisome proliferator receptor elements (PPREs) in their promoters and include:

1) genes involved in fatty acid oxidation and ketogenesis (Acox1, Cyp4a, Acadm, Hmgcs2);

2) genes involved in fatty acid transport (Cd36, , Slc27a1, Fabp1, Cpt1a, Cpt2);

3) genes involved in producing fatty acids and very low density lipoproteins (Me1, Scd1);

4) genes encoding apolipoproteins (Apoa1, Apoa2, Apoa5);

5) genes involved in triglyceride clearance (Angptl4);

6) genes involved in glycerol metabolism (Gpd1 in mouse);

7) genes involved in glucose metabolism (Pdk4);

8) genes involved in peroxisome proliferation (Pex11a);

9) genes involved in lipid storage (Plin, Adfp).

Many other genes are known to be regulated by PPAR-alpha but whether their regulation is direct or indirect remains to be found. These genes include: ACACA, FAS, SREBP1, FADS1, DGAT1, ABCA1, PLTP, ABCB4, UGT2B4, SULT2A1, Pnpla2, Acsl1, Slc27a4, many Acot genes, and others (reviewed in Rakhshandehroo et al. 2010).

References

Mandard S, Kersten S & Muller M (2004). Peroxisome proliferator-activated receptor alpha target genes. Cell Mol Life Sci, 61, 393-416.

Wahli W & Desvergne B (1999). Peroxisome proliferator-activated receptors: nuclear control of metabolism. Endocr Rev, 20, 649-88.

Kersten S, Knoch B, Rakhshandehroo M & Müller M (2010). Peroxisome proliferator-activated receptor alpha target genes. PPAR Res, 2010.

Qi C, Reddy JK & Zhu Y (2000). Peroxisome proliferator-activated receptors, coactivators, and downstream targets. Cell Biochem Biophys, 32, 187-204.

Kersten S (2008). Peroxisome proliferator activated receptors and lipoprotein metabolism. PPAR Res, 2008, 132960.

Edit history

Date	Action	Author
2009-06-08	Reviewed	Kersten S
2011-11-08	Edited	May B
2011-11-08	Authored	May B
2011-11-13	Created	May B
2020-11-10	Modified	D'Eustachio P

3 submitted entities found in this pathway, mapping to 3 Reactome entities

Input	UniProt Id	Input	UniProt Id	Input	UniProt Id
ACADM_HUMAN	P11310	CPT2_HUMAN	P23786	MAOX_HUMAN	P48163

20. Regulation of lipid metabolism by PPARalpha (R-HSA-400206)

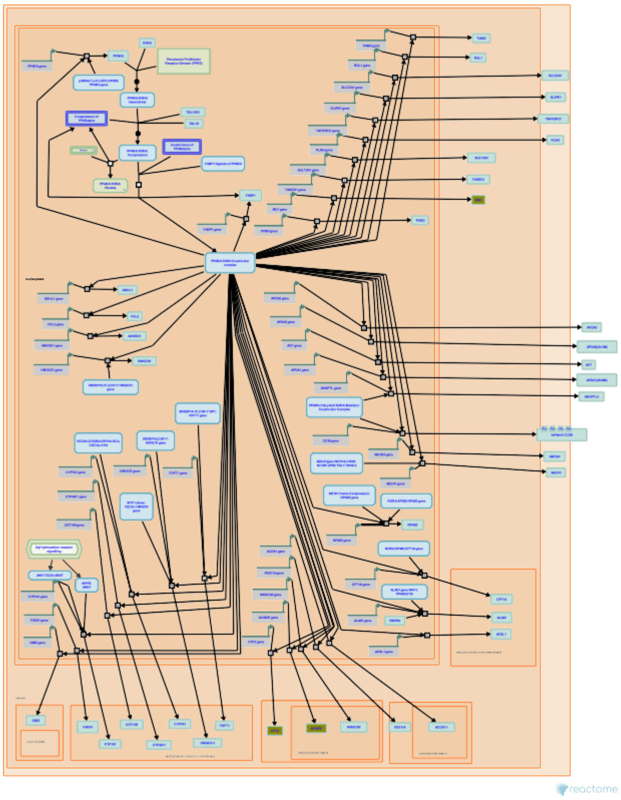

Cellular compartments: nucleoplasm, cytosol.

Peroxisome proliferator-activated receptor alpha (PPAR-alpha) is the major regulator of fatty acid oxidation in the liver. PPARalpha is also the target of fibrate drugs used to treat abnormal plasma lipid levels.

PPAR-alpha is a type II nuclear receptor (its subcellular location does not depend on ligand binding). PPAR-alpha forms heterodimers with Retinoid X receptor alpha (RXR-alpha), another type II nuclear receptor. PPAR-alpha is activated by binding fatty acid ligands, especially polyunsaturated fatty acids having 18-22 carbon groups and 2-6 double bonds.

The PPAR-alpha:RXR-alpha heterodimer binds peroxisome proliferator receptor elements (PPREs) in and around target genes. Binding of fatty acids and synthetic ligands causes a conformational change in PPAR-alpha such that it releases the corepressors and binds coactivators (CBP-SRC-HAT complex, ASC complex, and TRAP-Mediator complex) which initiate transcription of the target genes.

Target genes of PPAR-alpha participate in fatty acid transport, fatty acid oxidation, triglyceride clearance, lipoprotein production, and cholesterol homeostasis.

References

Gouni-Berthold I & Krone W (2005). Peroxisome proliferator-activated receptor alpha (PPARalpha) and athero-sclerosis. Curr Drug Targets Cardiovasc Haematol Disord, 5, 513-23.

Gelman L, Wahli W, Michalik L, Desvergne B & Feige JN (2006). From molecular action to physiological outputs: peroxisome proliferator-activated receptors are nuclear receptors at the crossroads of key cellular functions. Prog Lipid Res, 45, 120-59.

Wahli W & Desvergne B (1999). Peroxisome proliferator-activated receptors: nuclear control of metabolism. Endocr Rev, 20, 649-88.

Kersten S (2008). Peroxisome proliferator activated receptors and lipoprotein metabolism. PPAR Res, 2008, 132960.

Edit history

Date	Action	Author
2009-03-24	Created	May B
2009-05-30	Edited	May B
2009-05-30	Authored	May B
2009-06-08	Edited	May B
2009-06-08	Reviewed	Kersten S
2011-11-08	Edited	May B
2011-11-13	Revised	May B
2022-05-20	Modified	Weiser JD

3 submitted entities found in this pathway, mapping to 3 Reactome entities

Input	UniProt Id	Input	UniProt Id	Input	UniProt Id
ACADM_HUMAN	P11310	CPT2_HUMAN	P23786	MAOX_HUMAN	P48163

21. mitochondrial fatty acid beta-oxidation of saturated fatty acids (R-HSA-77286)

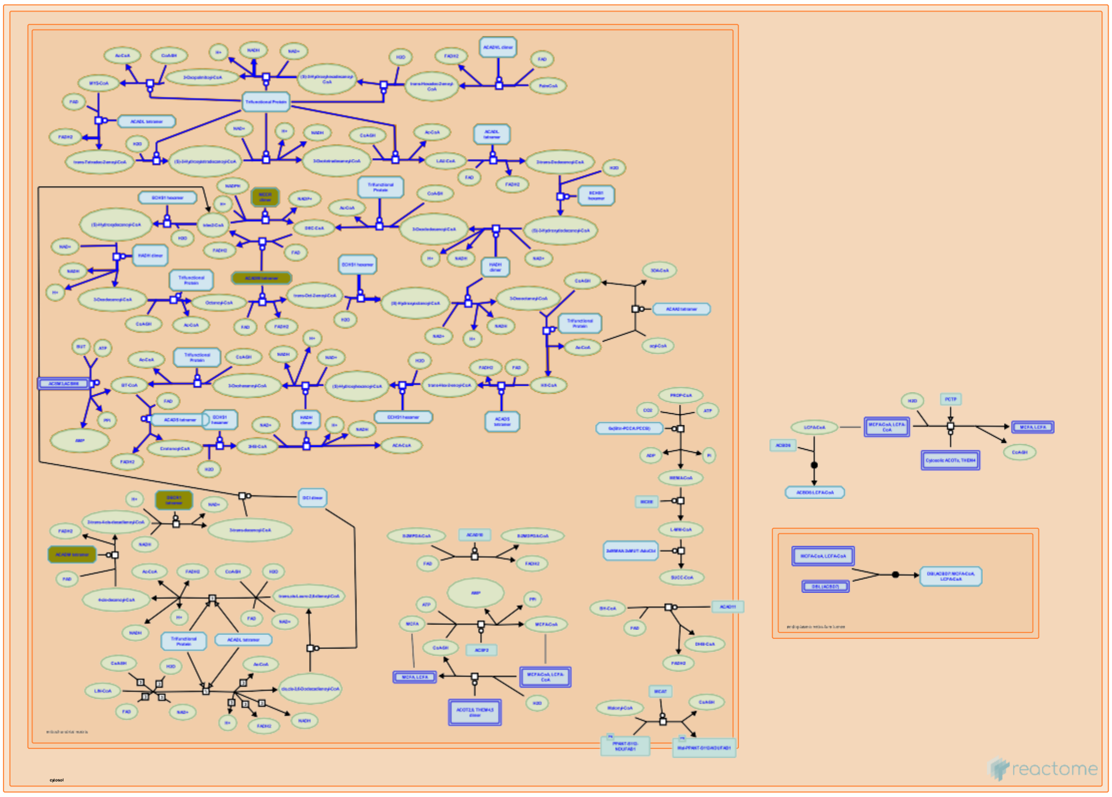

Cellular compartments: mitochondrial matrix.

Once fatty acids have been imported into the mitochondrial matrix by the carnitine acyltransferases, the beta-oxidation spiral begins. Each turn of this spiral concludes with the repetitive removal of two carbon units from the fatty acyl chain. beta-oxidation of saturated fatty acids (fatty acids with even numbered carbon chains and no double bonds) involves four different enzymatic steps: oxidation, hydration, a second oxidation, and a concluding thiolysis step, resulting in the two-carbon acetyl-CoA and a newly CoA primed acyl-CoA for the next turn of the spiral.

References

Beaudet AL, Scriver CR, Sly WS & Valle D (2001). *Mitochondrial fatty acid oxidation disorders, The Metabolic and Molecular Bases of Inherited Disease, 8th ed* , 2297-2326.

Edit history

Date	Action	Author
2003-09-19	Authored	Gillespie ME
2003-09-19	Created	Gillespie ME
2022-05-18	Edited	Gillespie ME
2022-05-20	Modified	Weiser JD

2 submitted entities found in this pathway, mapping to 2 Reactome entities

Input	UniProt Id	Input	UniProt Id
ACADM_HUMAN	P11310	MECR_HUMAN	Q9BV79

22. Branched-chain amino acid catabolism (R-HSA-70895)

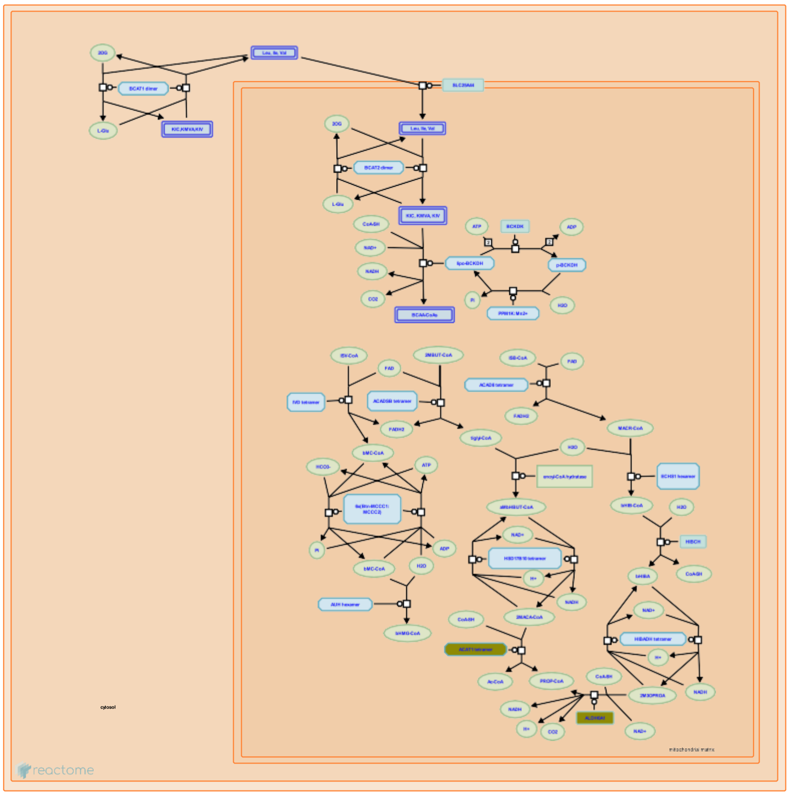

The branched-chain amino acids, leucine, isoleucine, and valine, are all essential amino acids (i.e., ones required in the diet). They are major constituents of muscle protein. The breakdown of these amino acids starts with two common steps catalyzed by enzymes that act on all three amino acids: reversible transamination by branched-chain amino acid aminotransferase, and irreversible oxidative decarboxylation by the branched-chain ketoacid dehydrogenase complex. Isovaleryl-CoA is produced from leucine by these two reactions, alpha-methylbutyryl-CoA from isoleucine, and isobutyryl-CoA from valine. These acyl-CoA's undergo dehydrogenation, catalyzed by three different but related enzymes, and the breakdown pathways then diverge. Leucine is ultimately converted to acetyl-CoA and acetoacetate; isoleucine to acetyl-CoA and succinyl-CoA; and valine to succinyl-CoA. Under fasting conditions, substantial amounts of all three amino acids are generated by protein breakdown. In muscle, the final products of leucine, isoleucine, and valine catabolism can be fully oxidized via the citric acid cycle; in liver they can be directed toward the synthesis of ketone bodies (acetoacetate and acetyl-CoA) and glucose (succinyl-CoA) (Neinast et al. 2019).

References

Arany Z, Murashige D & Neinast M (2019). Branched Chain Amino Acids. Annu. Rev. Physiol., 81, 139-164.

Edit history

Date	Action	Author
2003-06-24	Authored	D'Eustachio P
2010-02-18	Revised	D'Eustachio P
2020-01-03	Revised	D'Eustachio P
2022-05-18	Edited	D'Eustachio P

Date	Action	Author
2022-05-21	Modified	Weiser JD

2 submitted entities found in this pathway, mapping to 2 Reactome entities

Input	UniProt Id	Input	UniProt Id
MMSA_HUMAN	Q02252	THIL_HUMAN	P24752

23. Metabolism of amino acids and derivatives (R-HSA-71291)

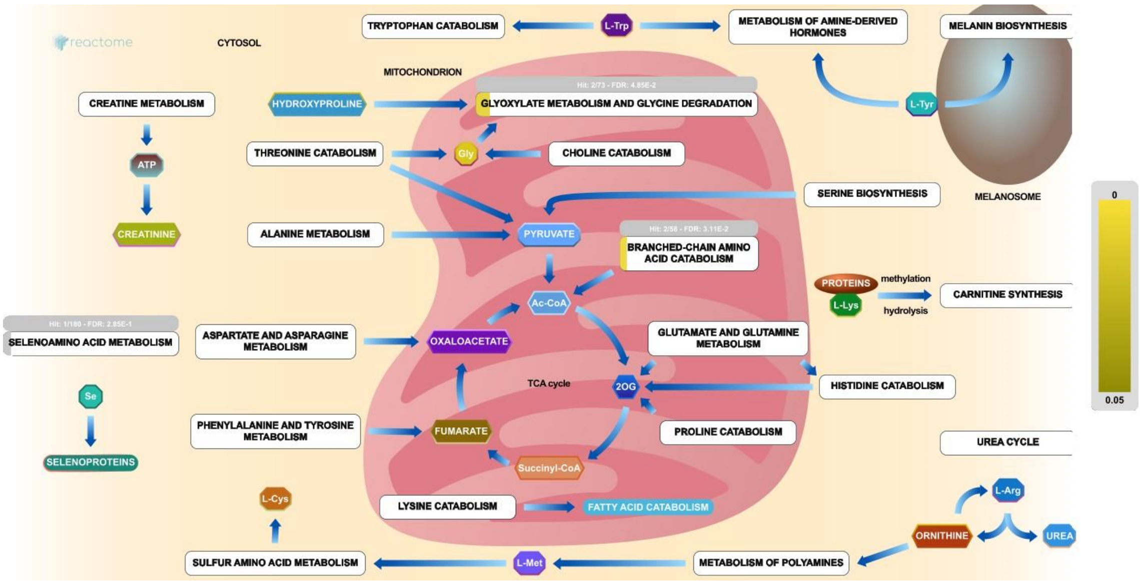

Cellular metabolism of amino acids and related molecules includes the pathways for the catabolism of amino acids, the biosynthesis of the nonessential amino acids (alanine, arginine, aspartate, asparagine, cysteine, glutamate, glutamine, glycine, proline, and serine) and selenocysteine, the synthesis of urea, and the metabolism of carnitine, creatine, choline, polyamides, melanin, and amine-derived hormones. The metabolism of amino acids provides a balanced supply of amino acids for protein synthesis. In the fasting state, the catabolism of amino acids derived from breakdown of skeletal muscle protein and other sources is coupled to the processes of gluconeogenesis and ketogenesis to meet the body’s energy needs in the absence of dietary energy sources. These metabolic processes also provide the nitrogen atoms for the biosynthesis of nucleotides and heme, annotated as separate metabolic processes (Felig 1975; Häussinger 1990; Owen et al. 1979).

Transport of these molecuels across lipid bilayer membranes is annotated separately as part of the module on "transmembrane transport of small molecules".

References

Reichard GA, Patel MS, Boden G & Owen OE (1979). Energy metabolism in feasting and fasting. Adv. Exp. Med. Biol., 111, 169-88.

Felig P (1975). Amino acid metabolism in man. Annu. Rev. Biochem., 44, 933-55.

Häussinger D (1990). Liver glutamine metabolism. JPEN J Parenter Enteral Nutr, 14, 56S-62S.

Edit history

Date	Action	Author
2003-10-11	Created	D'Eustachio P
2003-11-03	Authored	D'Eustachio P
2010-02-18	Revised	D'Eustachio P
2022-05-18	Edited	D'Eustachio P
2022-05-21	Modified	Weiser JD

5 submitted entities found in this pathway, mapping to 5 Reactome entities

Input	UniProt Id	Input	UniProt Id	Input	UniProt Id
MMSA_HUMAN	Q02252	ODP2_HUMAN	P10515	ODPA_HUMAN	P08559
RS12_HUMAN	P25398	THIL_HUMAN	P24752		

24. Signaling by Retinoic Acid (R-HSA-5362517)

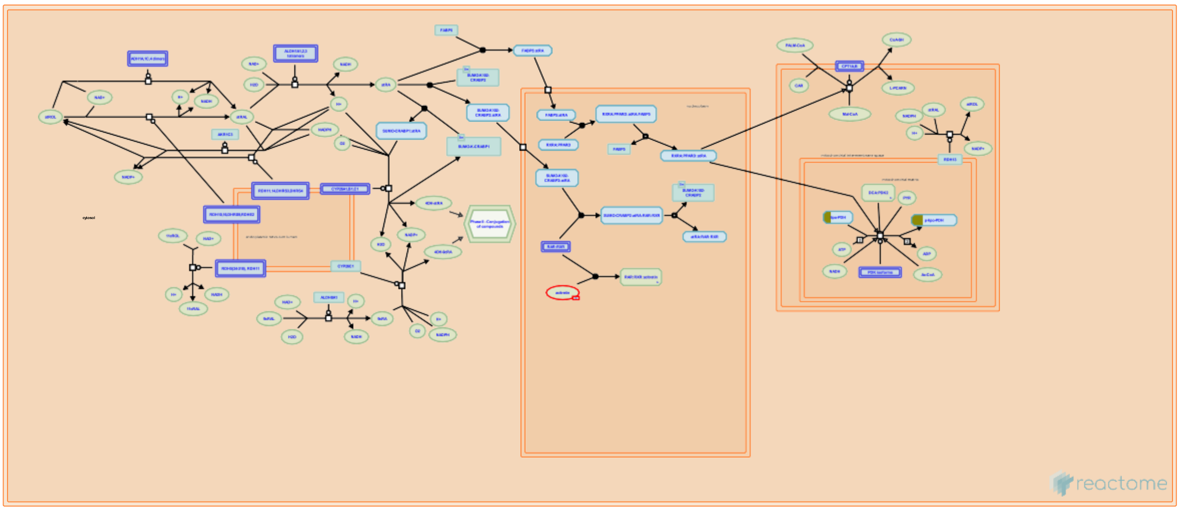

Vitamin A (retinol) can be metabolised into active retinoid metabolites that function either as a chromophore in vision or in regulating gene expression transcriptionally and post-transcriptionally. Genes regulated by retinoids are essential for reproduction, embryonic development, growth, and multiple processes in the adult, including energy balance, neurogenesis, and the immune response. The retinoid used as a cofactor in the visual cycle is 11-cis-retinal (11cRAL). The non-visual cycle effects of retinol are mediated by retinoic acid (RA), generated by two-step conversion from retinol (Napoli 2012). All-trans-retinoic acid (atRA) is the major activated metabolite of retinol. An isomer, 9-cis-retinoic acid (9cRA) has biological activity, but has not been detected in vivo, except in the pancreas. An alternative route involves BCO1 cleavage of carotenoids into retinal, which is then reduced into retinol in the intestine (Harrison 2012). The two isomers of RA serve as ligands for retinoic acid receptors (RAR) that regulate gene expression. (Das et al. 2014). RA is catabolised to oxidised metabolites such as 4-hydroxy-, 18-hydroxy- or 4-oxo-RA by CYP family enzymes, these metabolites then becoming substrates for Phase II conjugation enzymes (Ross & Zolfaghari 2011).

References

Harrison EH (2012). Mechanisms involved in the intestinal absorption of dietary vitamin A and provitamin A carotenoids. Biochim. Biophys. Acta, 1821, 70-7.

Napoli JL (2012). Physiological insights into all-trans-retinoic acid biosynthesis. Biochim. Biophys. Acta, 1821, 152-67.

Ross AC & Zolfaghari R (2011). Cytochrome P450s in the regulation of cellular retinoic acid metabolism. Annu. Rev. Nutr., 31, 65-87.

Karki R, Torregroza I, Mahapatra S, Kambhampati S, Das S, Evans T, ... Liu TC (2014). Retinoic acid signaling pathways in development and diseases. Bioorg. Med. Chem., 22, 673-83.

Edit history

Date	Action	Author
2014-04-16	Edited	Jassal B
2014-04-16	Authored	Jassal B
2014-04-16	Created	Jassal B
2014-07-28	Reviewed	Duester G

Date	Action	Author
2014-09-01	Reviewed	Napoli JL
2022-05-21	Modified	Weiser JD

2 submitted entities found in this pathway, mapping to 2 Reactome entities

Input	UniProt Id	Input	UniProt Id
ODP2_HUMAN	P10515	ODPA_HUMAN	P08559

25. Glyoxylate metabolism and glycine degradation (R-HSA-389661)

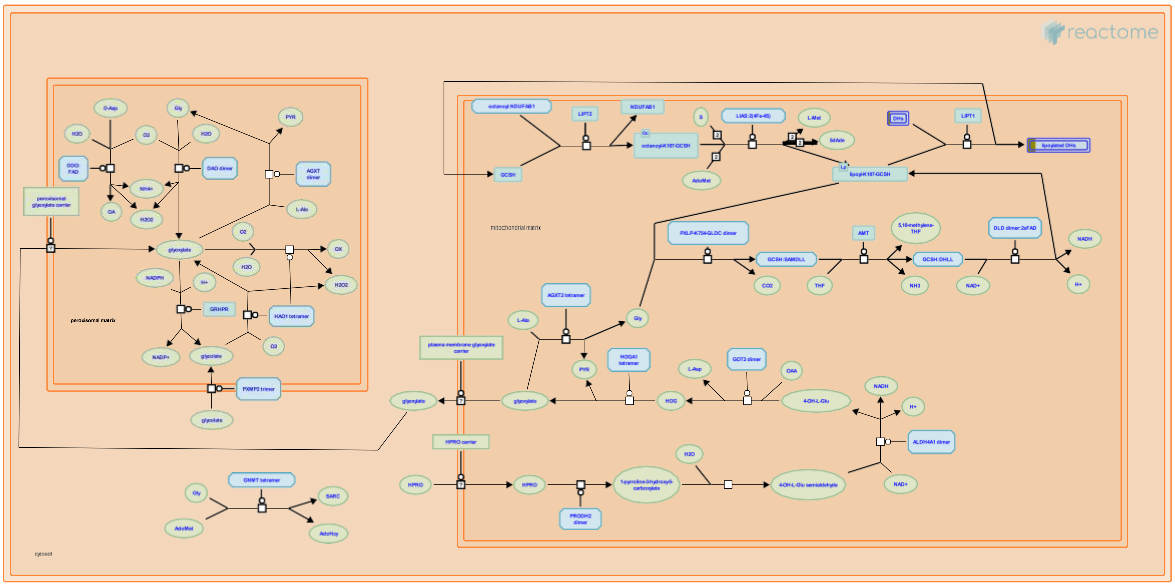

Glyoxylate is generated in the course of glycine and hydroxyproline catabolism and can be converted to oxalate. In humans, this process takes place in the liver. Defects in two enzymes of glyoxylate metabolism, alanine:glyoxylate aminotransferase (AGXT) and glycerate dehydrogenase/glyoxylate reductase (GRHPR), are associated with pathogenic overproduction of oxalate (Danpure 2005). The reactions that interconvert glycine, glycolate, and glyoxylate and convert glyoxylate to oxalate have been characterized in molecular detail in humans. A reaction sequence for the conversion of hydroxyproline to glyoxylate has been inferred from studies of partially purified extracts of rat and bovine liver but the enzymes involved in the corresponding human reactions have not been identified.

References

Danpure CJ (2005). Primary hyperoxaluria: from gene defects to designer drugs?. Nephrol Dial Transplant, 20, 1525-9.

Edit history

Date	Action	Author
2009-01-12	Authored	D'Eustachio P
2009-01-12	Created	D'Eustachio P
2009-03-03	Edited	D'Eustachio P
2009-03-03	Reviewed	Jassal B
2022-05-21	Modified	Weiser JD

2 submitted entities found in this pathway, mapping to 2 Reactome entities

Input	UniProt Id	Input	UniProt Id
ODP2_HUMAN	P10515	ODPA_HUMAN	P08559

6. Identifiers found

Below is a list of the input identifiers that have been found or mapped to an equivalent element in Reactome, classified by resource.

26 of the submitted entities were found, mapping to 26 Reactome entities

Input	UniProt Id	Input	UniProt Id	Input	UniProt Id
ACACA_HUMAN	Q13085	ACADM_HUMAN	P11310	ACLY_HUMAN	P53396
ARP3_HUMAN	P61158	ATPB_HUMAN	P06576	CPT2_HUMAN	P23786
DECR_HUMAN	Q16698	DX39A_HUMAN	O00148	FETA_HUMAN	P02771
HNRPK_HUMAN	P61978	HPT_HUMAN	P00738	HSP74_HUMAN	P34932
IDH3A_HUMAN	P50213	MAOX_HUMAN	P48163	MECR_HUMAN	Q9BV79
MMSA_HUMAN	Q02252	ODP2_HUMAN	P10515	ODPA_HUMAN	P08559
PLMN_HUMAN	P00747	PYC_HUMAN	P11498	QCR6_HUMAN	P07919
RAB14_HUMAN	P61106	RS12_HUMAN	P25398	SDHA_HUMAN	P31040
SSBP_HUMAN	Q04837	THIL_HUMAN	P24752		

7. Identifiers not found

These 2 identifiers were not found neither mapped to any entity in Reactome.

EST1C_HUMAN PPIF_HUMAN
