# Supplementary material for: Brown Adipose Tissue Sheds Extracellular Vesicles That Carry Potential Biomarkers of Metabolic and Thermogenesis Activity Which Are Affected by High Fat Diet Intervention
Source: Int J Mol Sci. 2022 Sep 16;23(18):10826. doi: 10.3390/ijms231810826 (PMC9504916; doi:10.3390/ijms231810826)
Supplement: Supplementary file 1 [file ijms-23-10826-s001.zip › Supplementary Materials file 2.pdf]

Pathway Analysis Report

GBM Uniprot

This report contains the pathway analysis results for the submitted sample 'GBM Uniprot'. Analysis was performed against Reactome version 81 on 27/07/2022. The web link to these results is:

https://reactome.org/PathwayBrowser/#/ANALYSIS=MjAyMjA2MTYxOTQ5MTRfMg%3D%3D

Please keep in mind that analysis results are temporarily stored on our server. The storage period depends on usage of the service but is at least 7 days. As a result, please note that this URL is only valid for a limited time period and it might have expired.

Table of Contents

1. Introduction
2. Properties
3. Genome-wide overview
4. Most significant pathways
5. Pathways details
6. Identifiers found
7. Identifiers not found

1. Introduction

Reactome is a curated database of pathways and reactions in human biology. Reactions can be considered as pathway 'steps'. Reactome defines a 'reaction' as any event in biology that changes the state of a biological molecule. Binding, activation, translocation, degradation and classical biochemical events involving a catalyst are all reactions. Information in the database is authored by expert biologists, entered and maintained by Reactome’s team of curators and editorial staff. Reactome content frequently cross-references other resources e.g. NCBI, Ensembl, UniProt, KEGG (Gene and Compound), ChEBI, PubMed and GO. Orthologous reactions inferred from annotation for Homo sapiens are available for 17 non-human species including mouse, rat, chicken, puffer fish, worm, fly, yeast, rice, and Arabidopsis. Pathways are represented by simple diagrams following an SBGN-like format.

Reactome's annotated data describe reactions possible if all annotated proteins and small molecules were present and active simultaneously in a cell. By overlaying an experimental dataset on these annotations, a user can perform a pathway over-representation analysis. By overlaying quantitative expression data or time series, a user can visualize the extent of change in affected pathways and its progression. A binomial test is used to calculate the probability shown for each result, and the p-values are corrected for the multiple testing (Benjamini–Hochberg procedure) that arises from evaluating the submitted list of identifiers against every pathway.

To learn more about our Pathway Analysis, please have a look at our relevant publications:

Fabregat A, Sidiropoulos K, Garapati P, Gillespie M, Hausmann K, Haw R, … D’Eustachio P (2016). The reactome pathway knowledgebase. Nucleic Acids Research, 44(D1), D481–D487. https://doi.org/10.1093/nar/gkv1351.

Fabregat A, Sidiropoulos K, Viteri G, Forner O, Marin-Garcia P, Arnau V, … Hermjakob H (2017). Reactome pathway analysis: a high-performance in-memory approach. BMC Bioinformatics, 18.

2. Properties

- This is an **overrepresentation** analysis: A statistical (hypergeometric distribution) test that determines whether certain Reactome pathways are over-represented (enriched) in the submitted data. It answers the question 'Does my list contain more proteins for pathway X than would be expected by chance?' This test produces a probability score, which is corrected for false discovery rate using the Benjamani-Hochberg method.
- 167 out of 184 identifiers in the sample were found in Reactome, where 1125 pathways were hit by at least one of them.
- All non-human identifiers have been converted to their human equivalent.
- This report is filtered to show only results for species 'Homo sapiens' and resource 'all resources'.
- The unique ID for this analysis (token) is MjAyMjA2MTYxOTQ5MTRfMg%3D%3D. This ID is valid for at least 7 days in Reactome's server. Use it to access Reactome services with your data.

3. Genome-wide overview

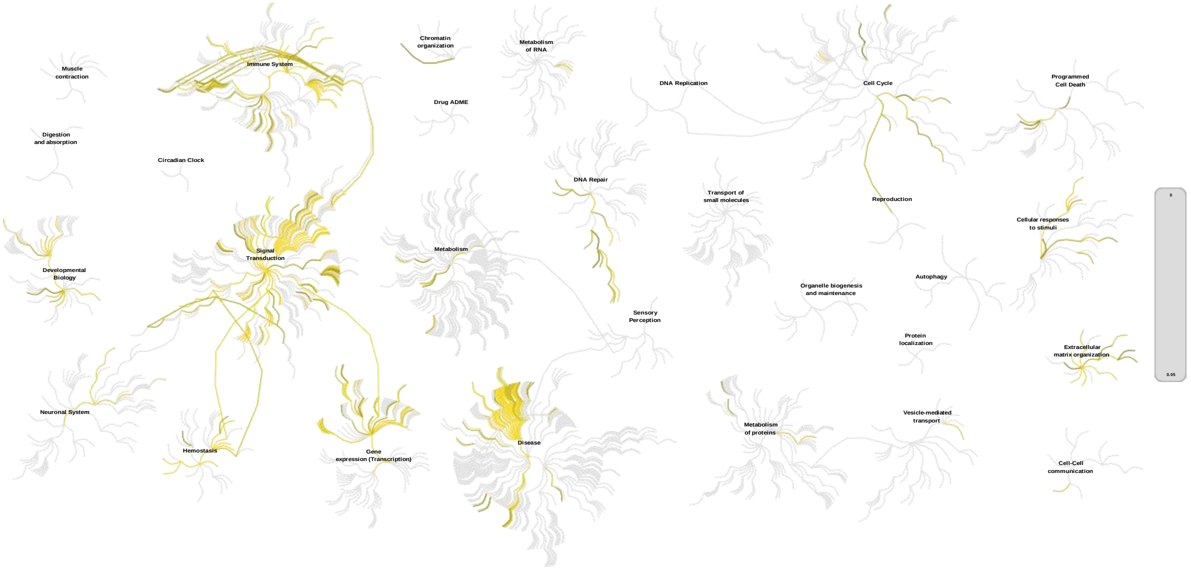

This figure shows a genome-wide overview of the results of your pathway analysis. Reactome pathways are arranged in a hierarchy. The center of each of the circular "bursts" is the root of one top-level pathway, for example "DNA Repair". Each step away from the center represents the next level lower in the pathway hierarchy. The color code denotes over-representation of that pathway in your input dataset. Light grey signifies pathways which are not significantly over-represented.

4. Most significant pathways

The following table shows the 25 most relevant pathways sorted by p-value.

Pathway name	Entities				Reactions	
	found	ratio	p-value	FDR*	found	ratio
Diseases of signal transduction by growth factor receptors and second messengers	54 / 498	0.033	1.11e-16	2.29e-14	359 / 478	0.034
Signaling by FGFR in disease	18 / 82	0.005	1.11e-16	2.29e-14	70 / 99	0.007
Signaling by Receptor Tyrosine Kinases	59 / 620	0.041	1.11e-16	2.29e-14	418 / 746	0.054
Signal Transduction	108 / 3,019	0.199	1.11e-16	2.29e-14	1,020 / 2,508	0.18
PI3K/AKT Signaling in Cancer	24 / 124	0.008	1.11e-16	2.29e-14	8 / 21	0.002
Intracellular signaling by second messengers	32 / 368	0.024	1.11e-16	2.29e-14	36 / 114	0.008
Negative regulation of the PI3K/AKT network	21 / 137	0.009	3.33e-16	5.86e-14	4 / 10	7.18e-04
PIP3 activates AKT signaling	29 / 321	0.021	5.55e-16	8.55e-14	31 / 86	0.006
Signaling by SCF-KIT	15 / 51	0.003	6.66e-16	9.13e-14	36 / 39	0.003
Signaling by VEGF	20 / 140	0.009	6.22e-15	7.65e-13	42 / 86	0.006
Disease	81 / 2,656	0.175	1.60e-14	1.79e-12	462 / 1,720	0.123
PI5P, PP2A and IER3 Regulate PI3K/AKT Signaling	19 / 129	0.009	1.79e-14	1.84e-12	2 / 7	5.02e-04
Constitutive Signaling by Aberrant PI3K in Cancer	17 / 96	0.006	2.43e-14	2.31e-12	2 / 2	1.44e-04
VEGFA-VEGFR2 Pathway	18 / 129	0.009	2.19e-13	1.93e-11	39 / 79	0.006
MAPK1/MAPK3 signaling	26 / 329	0.022	3.99e-13	3.27e-11	43 / 82	0.006
Signaling by PDGF	14 / 70	0.005	1.01e-12	7.74e-11	28 / 31	0.002
Insulin receptor signalling cascade	14 / 72	0.005	1.46e-12	1.05e-10	15 / 25	0.002
MAPK family signaling cascades	27 / 380	0.025	1.56e-12	1.06e-10	47 / 122	0.009
RAF/MAP kinase cascade	25 / 322	0.021	1.71e-12	1.11e-10	41 / 75	0.005
Signaling by FGFR1 in disease	12 / 49	0.003	4.33e-12	2.59e-10	35 / 35	0.003
Downstream signal transduction	11 / 37	0.002	4.46e-12	2.59e-10	16 / 16	0.001
Signaling by Insulin receptor	15 / 97	0.006	5.62e-12	3.15e-10	23 / 34	0.002
IRS-mediated signalling	13 / 65	0.004	6.61e-12	3.37e-10	4 / 9	6.46e-04
Signaling by KIT in disease	10 / 28	0.002	7.26e-12	3.41e-10	26 / 26	0.002
Signaling by phosphorylated juxtamembrane, extracellular and kinase domain KIT mutants	10 / 28	0.002	7.26e-12	3.41e-10	11 / 11	7.90e-04

* False Discovery Rate

5. Pathways details

For every pathway of the most significant pathways, we present its diagram, as well as a short summary, its bibliography and the list of inputs found in it.

1. Diseases of signal transduction by growth factor receptors and second messengers (R-HSA-5663202)

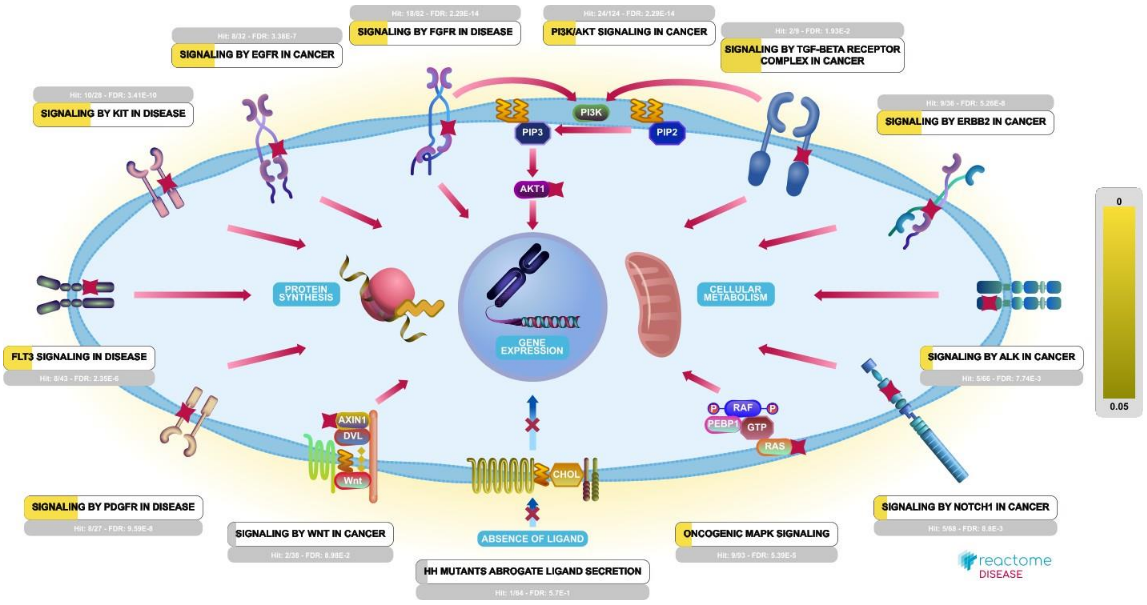

Signaling processes are central to human physiology (e.g., Pires-da Silva & Sommer 2003), and their disruption by either germ-line and somatic mutation can lead to serious disease. Here, the molecular consequences of mutations affecting visual signal transduction and signaling by diverse growth factors are annotated.

References

Pires-daSilva A & Sommer RJ (2003). The evolution of signalling pathways in animal development. Nat. Rev. Genet., 4, 39-49.

Edit history

Date	Action	Author
2015-01-16	Created	D'Eustachio P
2021-03-29	Modified	Rothfels K

43 submitted entities found in this pathway, mapping to 54 Reactome entities

Input	UniProt Id	Input	UniProt Id	Input	UniProt Id
O15164	O15164	O43524	O43524	P00533	P00533
P01111	P01111	P01116	P01116, P01116-1, P01116-2	P02751	P02751
P04626	P04626	P05106	P05106	P07900	P07900
P07948	P07948	P08581	P08581	P09619	P09619

Input	UniProt Id	Input	UniProt Id	Input	UniProt Id
P10721	P10721	P11274	P11274	P11309	P11309
P11362	P11362, P11362-1, P11362-19	P16234	P16234	P21802	P21802, P21802-1, P21802-17, P21802-18, P21802-3, P21802-5
P21860	P21860-1	P22681	P22681	P27986	P27986
P35222	P35222	P35568	P35568	P37173	P37173
P40763	P40763	P42224	P42224, P42224-1	P42336	P42336
P42345	P42345	P46531	P46531	P46940	P46940
P49815	P49815	P55211	P55211	P60484	P60484
P78504	P78504	P98177	P98177	Q00987	Q00987
Q06124	Q06124	Q09472	Q09472	Q13485	Q13485
Q14738	Q14738	Q15465	Q15465	Q7Z5R6	Q7Z5R6
Q969H0	Q969H0-1, Q969H0-4				

2. Signaling by FGFR in disease (R-HSA-1226099)

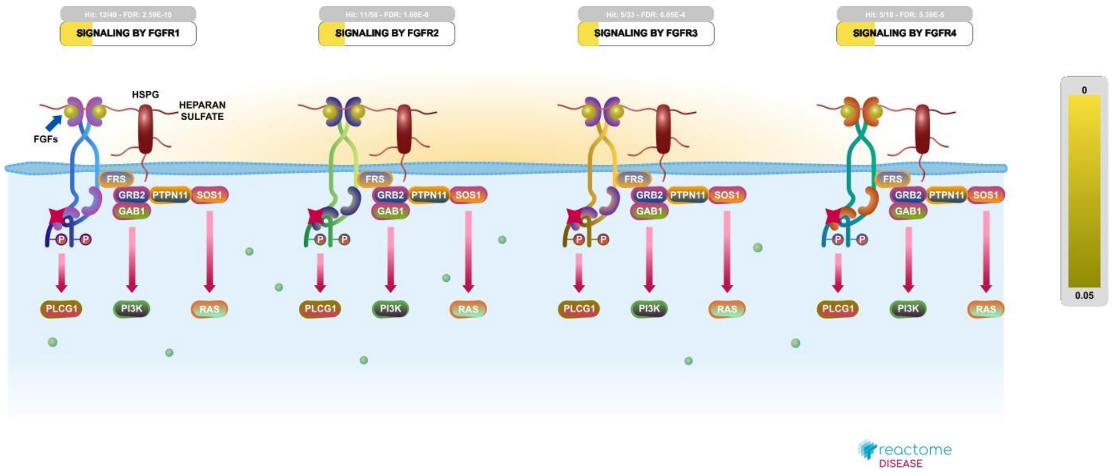

Diseases: cancer, bone development disease.

A number of skeletal and developmental diseases have been shown to arise as a result of mutations in the FGFR1, 2 and 3 genes. These include dwarfism syndromes (achondroplasia, hypochondroplasia and the neonatal lethal disorders thanatophoric dysplasia I and II), as well as craniosynostosis disorders such as Pfeiffer, Apert, Crouzon, Jackson-Weiss and Muenke syndromes (reviewed in Webster and Donoghue 1997; Burke, 1998, Cunningham, 2007; Harada, 2009). These mutations fall into four general regions of the receptor: a) the immunoglobulin (Ig)-like domain II-III linker region, b) the alternatively spliced second half of the Ig III domain, c) the transmembrane domain and d) the tyrosine kinase domain (reviewed in Webster and Donoghue, 1997). With the exception of mutations in class b), which affect only the relevant splice variant, these mutations may be present in either the 'b' or 'c' isoforms. These activating mutations affect FGFR function by altering or expanding the ligand-binding range of the receptors (see for instance Ibrahimi, 2004a), by promoting ligand-independent dimerization (for instance, Galvin,1996; Neilson and Friesel, 1996; d'Avis,1998) or by increasing the activity of the kinase domain (for instance, Webster, 1996; Naski, 1996; Tavormina, 1999; Bellus, 2000). Thus, a number of the point mutations found in FGFR receptors alter their activity without altering their intrinsic kinase activity. Many of the mutations that promote constitutive dimerization do so by creating or removing cysteine residues; the presence of an unpaired cysteine in the receptor is believed to promote dimerization through the formation of intramolecular disulphide bonds (Galvin, 1996; Robertson, 1998). Paralogous mutations at equivalent positions have been identified in more than one FGF receptor, sometimes giving rise to different diseases. For instance, mutation of the highly conserved FGFR2 Ser252-Pro253 dipeptide in the region between the second and third Ig domain is responsible for virtually all cases of Apert Syndrome (Wilkie, 1995), while paralogous mutations in FGFR1 (S252R) and FGFR3 (P250R) are associated with Pfeiffer and Crouzon syndromes, respectively (Bellus, 1996). FGFR4 is unique in that mutations of this gene are not known to be associated with any developmental disorders.

Recently, many of the same activating mutations in the FGFR genes that have been characterized in skeletal and developmental disorders have begun to be identified in a range of cancers (reviewed in Turner and Gross, 2010; Greulich and Pollock, 2011; Wesche, 2011). The best established link between a somatic mutation of an FGFR and the development of cancer is in the case of FGFR3, where 50% of bladder cancers have mutations in the FGFR3 coding sequence. Of these mutations, which largely match the activating mutations seen in thanatophoric dysplasias, over half occur at a single residue (S249C) (Cappellen, 1999; van Rhijn, 2002). Activating mutations have also been identified in the coding sequences of FGFR1, 2 and 4 (for review, see Wesche, 2011)

In addition to activating point mutations, the FGFR1, 2 and 3 genes are subject to misregulation in cancer through gene amplification and translocation events, which are thought to lead to overexpression and ligand-independent dimerization (Weiss, 2010; Turner, 2010; Kunii, 2008; Takeda, 2007; Chesi, 1997; Avet-Loiseau, 1998; Ronchetti, 2001). It is important to note, however, that in each of these cases, the amplification or translocation involve large genomic regions encompassing additional genes, and the definitive roles of the FGFR genes in promoting oncogenesis has not been totally established. In the case of FGFR1, translocation events also give rise to FGFR1 fusion proteins that contain the intracellular kinase domain of the receptor fused to a dimerization domain from the partner gene. These fusions, which are expressed in a pre-leukemic myeloproliferative syndrome, dimerize constitutively based on the dimerization domain provided by the fusion partner and are constitutively active (reviewed in Jackson, 2010).

References

Kuehl WM, Nardini E, Chesi M, Brents LA, Schröck E, Bergsagel PL & Ried T (1997). Frequent translocation t(4;14)(p16.3;q32.3) in multiple myeloma is associated with increased expression and activating mutations of fibroblast growth factor receptor 3. Nat Genet, 16, 260-4.

Ornitz DM, Wang Q, Xu J & Naski MC (1996). Graded activation of fibroblast growth factor receptor 3 by mutations causing achondroplasia and thanatophoric dysplasia. Nat Genet, 13, 233-7.

Galvin BD, Donoghue DJ, Hart KC, Robertson SC, Webster MK & Meyer AN (1998). Activating mutations in the extracellular domain of the fibroblast growth factor receptor 2 function by disruption of the disulfide bond in the third immunoglobulin-like domain. Proc Natl Acad Sci U S A, 95 , 4567-72.

Grose RP & Turner N (2010). Fibroblast growth factor signalling: from development to cancer. Nat Rev Cancer, 10, 116-29.

Jackson CC, Medeiros LJ & Miranda RN (2010). 8p11 myeloproliferative syndrome: a review. Hum Pathol, 41, 461-76.

Edit history

Date	Action	Author
2011-03-09	Created	Rothfels K
2012-02-10	Authored	Rothfels K
2012-05-15	Edited	Rothfels K
2012-05-15	Reviewed	Ezzat S
2016-01-25	Reviewed	Grose RP
2021-05-04	Modified	Matthews L

10 submitted entities found in this pathway, mapping to 18 Reactome entities

Input	UniProt Id
O15164	O15164
P11274	P11274
P27986	P27986
P42336	P42336

Input	UniProt Id
P01111	P01111
P11362	P11362, P11362-1, P11362-19
P40763	P40763

Input	UniProt Id
P01116	P01116-1, P01116-2
P21802	P21802, P21802-1, P21802-17, P21802-18, P21802-3, P21802-5
P42224	P42224

3. Signaling by Receptor Tyrosine Kinases (R-HSA-9006934)

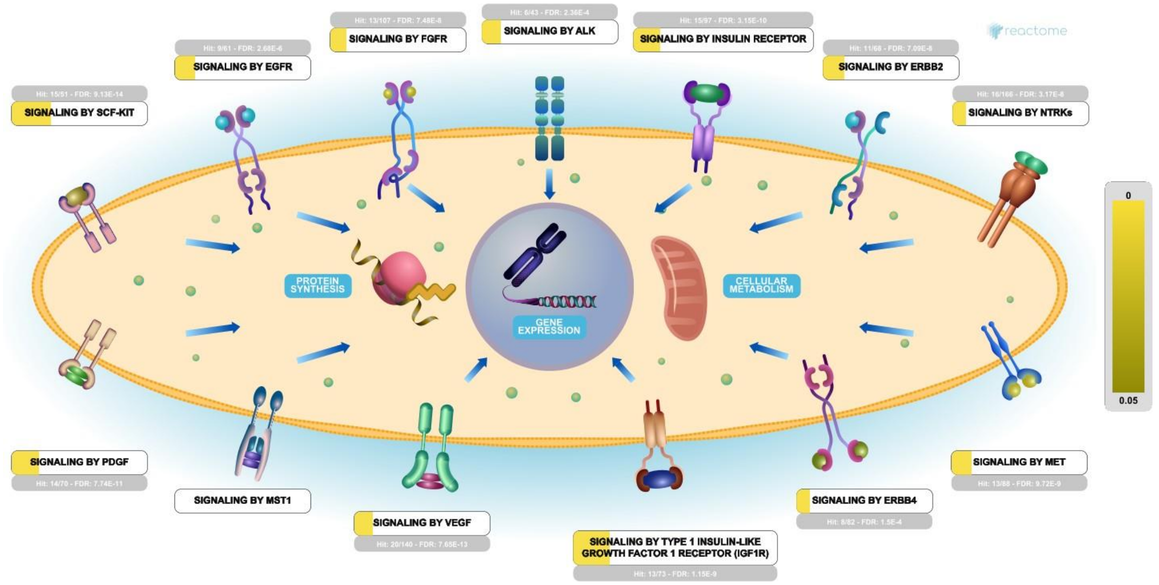

Receptor tyrosine kinases (RTKs) are a major class of cell surface proteins involved in Signal Transduction. Human cells contain ~60 RTKs, grouped into 20 subfamilies based on their domain architecture. All RTK subfamilies are characterized by an extracellular ligand-binding domain, a single transmembrane region and an intracellular region consisting of the tyrosine kinase domain and additional regulatory and protein interaction domains. In general, RTKs associate into dimers upon ligand binding and are activated by autophosphorylation on conserved intracellular tyrosine residues. Autophosphorylation increases the catalytic efficiency of the receptor and provides binding sites for the assembly of downstream signaling complexes (reveiwed in Lemmon and Schlessinger, 2010). Common signaling pathways activated downstream of RTK activation include RAF/MAP kinase cascades (reviewed in McKay and Morrison, 2007 and Wellbrock et al 2004), AKT signaling (reviewed in Manning and Cantley, 2007) and PLC-gamma mediated signaling (reviewed in Patterson et al). Activation of these pathways ultimately results in changes in gene expression and cellular metabolism.

References

Snyder SH, Nikolaidis N, van Rossum DB, Gill DL & Patterson RL (2005). Phospholipase C-gamma: diverse roles in receptor-mediated calcium signaling. Trends Biochem Sci, 30, 688-97.

Wellbrock C, Karasarides M & Marais R (2004). The RAF proteins take centre stage. Nat Rev Mol Cell Biol, 5, 875-85.

Manning BD & Cantley LC (2007). AKT/PKB signaling: navigating downstream. Cell, 129, 1261-74.

McKay MM & Morrison DK (2007). Integrating signals from RTKs to ERK/MAPK. Oncogene, 26, 3113-21.

Schlessinger J & Lemmon MA (2010). Cell signaling by receptor tyrosine kinases. Cell, 141, 1117-34.

Edit history

Date	Action	Author
2017-05-24	Edited	Rothfels K
2017-05-24	Authored	Rothfels K
2017-05-24	Created	Rothfels K
2017-06-22	Reviewed	D'Eustachio P
2022-05-21	Modified	Weiser JD

53 submitted entities found in this pathway, mapping to 59 Reactome entities

Input	UniProt Id	Input	UniProt Id	Input	UniProt Id
O14757	O14757	O15264	O15264	O15524	O15524
O43184	O43184	P00533	P00533	P01111	P01111
P01116	P01116-1, P01116-2	P02452	P02452	P02461	P02461
P02751	P02751	P04198	P04198	P04626	P04626
P05106	P05106	P05771	P05771	P06213	P06213
P07900	P07900	P07948	P07948	P08123	P08123
P08581	P08581	P09486	P09486	P09619	P09619
P09958	P09958	P10721	P10721	P11362	P11362-1, P11362-19
P12110	P12110	P16234	P16234	P17252	P17252
P17948	P17948	P21802	P21802-1, P21802-18, P21802-3, P21802-5	P21860	P21860-1
P22681	P22681	P27986	P27986	P29474	P29474
P35222	P35222	P35568	P35568	P35916	P35916
P36543	P36543	P40763	P40763	P42224	P42224, P42224-1
P42336	P42336	P42345	P42345	P51812	P51812
P52735	P52735	P56945	P56945	Q05513	Q05513
Q05655	Q05655	Q06124	Q06124	Q07812	Q07812
Q09472	Q09472	Q13164	Q13164	Q14185	Q14185
Q14738	Q14738	Q99081	Q99081		

4. Signal Transduction (R-HSA-162582)

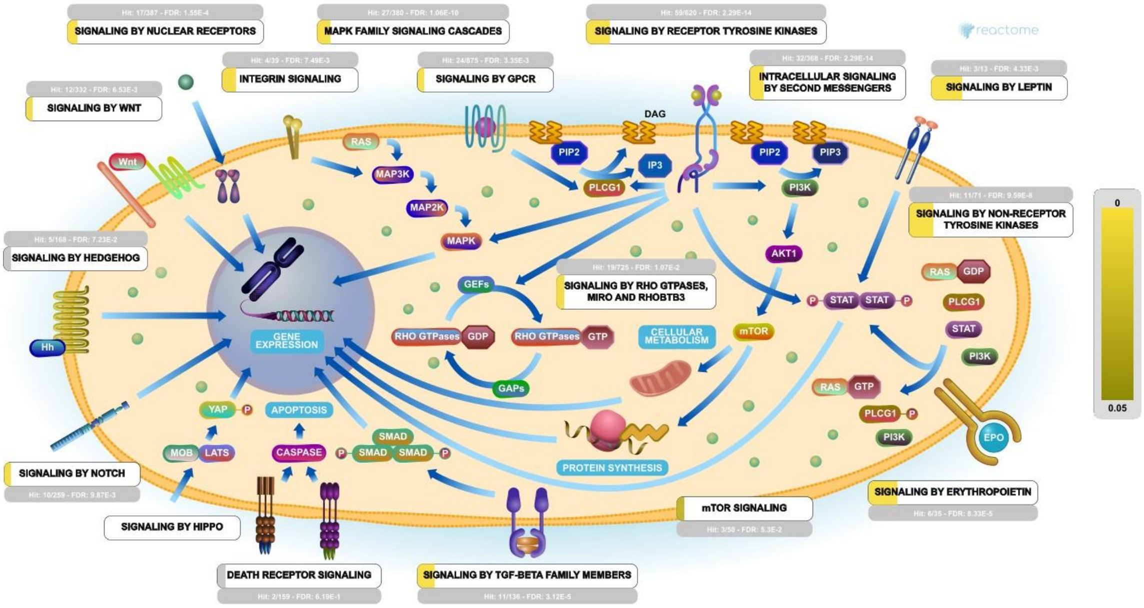

Signal transduction is a process in which extracellular signals elicit changes in cell state and activity. Transmembrane receptors sense changes in the cellular environment by binding ligands, such as hormones and growth factors, or reacting to other types of stimuli, such as light. Stimulation of transmembrane receptors leads to their conformational change which propagates the signal to the intracellular environment by activating downstream signaling cascades. Depending on the cellular context, this may impact cellular proliferation, differentiation, and survival. On the organism level, signal transduction regulates overall growth and behavior.

Receptor tyrosine kinases (RTKs) transmit extracellular signals by phosphorylating their protein partners on conserved tyrosine residues. Some of the best studied RTKs are EGFR (reviewed in Avraham and Yarden, 2011), FGFR (reviewed in Eswarakumar et al, 2005), insulin receptor (reviewed in Saltiel and Kahn, 2001), NGF (reviewed in Reichardt, 2006), PDGF (reviewed in Andrae et al, 2008) and VEGF (reviewed in Xie et al, 2004). RTKs frequently activate downstream signaling through RAF/MAP kinases (reviewed in McKay and Morrison, 2007 and Wellbrock et al 2004), AKT (reviewed in Manning and Cantley, 2007) and PLC- gamma (reviewed in Patterson et al, 2005), which ultimately results in changes in gene expression and cellular metabolism.

Receptor serine/threonine kinases of the TGF-beta family, such as TGF-beta receptors (reviewed in Kang et al. 2009) and BMP receptors (reviewed in Miyazono et al. 2009), transmit extracellular signals by phosphorylating regulatory SMAD proteins on conserved serine and threonine residues. This leads to formation of complexes of regulatory SMADs and SMAD4, which translocate to the nucleus where they act as transcription factors.

WNT receptors transmit their signal through beta-catenin. In the absence of ligand, beta-catenin is constitutively degraded in a ubiquitin-dependent manner. WNT receptor stimulation releases beta-catenin from the destruction complex, allowing it to translocate to the nucleus where it acts as a transcriptional regulator (reviewed in MacDonald et al, 2009 and Angers and Moon, 2009). WNT receptors were originally classified as G-protein coupled receptors (GPCRs). Although they are structurally related, GPCRs primarily transmit their signals through G-proteins, which are trimers of alpha, beta and gamma subunits. When a GPCR is activated, it acts as a guanine nucleotide exchange factor, catalyzing GDP to GTP exchange on the G-alpha subunit of the G protein and its dissociation from the gamma-beta heterodimer. The G-alpha subunit regulates the activity of adenylate cyclase, while the gamma-beta heterodimer can activate AKT and PLC signaling (reviewed in Rosenbaum et al. 2009, Oldham and Hamm 2008, Ritter and Hall 2009).

NOTCH receptors are activated by transmembrane ligands expressed on neighboring cells, which results in cleavage of NOTCH receptor and release of its intracellular domain. NOTCH intracellular domain translocates to the nucleus where it acts as a transcription factor (reviewed in Kopan and Ilagan, 2009).

Integrins are activated by extracellular matrix components, such as fibronectin and collagen, leading to conformational change and clustering of integrins on the cell surface. This results in activation of integrin-linked kinase and other cytosolic kinases and, in co-operation with RTK signaling, regulates survival, proliferation and cell shape and adhesion (reviewed in Hehlgans et al, 2007) .

Besides inducing changes in gene expression and cellular metabolism, extracellular signals that trigger the activation of Rho GTP-ases can trigger changes in the organization of cytoskeleton, thereby regulating cell polarity and cell-cell junctions (reviewed in Citi et al, 2011).

References

Hall RA & Ritter SL (2009). Fine-tuning of GPCR activity by receptor-interacting proteins. Nat Rev Mol Cell Biol, 10, 819-30.

Yarden Y & Avraham R (2011). Feedback regulation of EGFR signalling: decision making by early and delayed loops. Nat Rev Mol Cell Biol, 12, 104-17.

MacDonald BT, Tamai K & He X (2009). Wnt/beta-catenin signaling: components, mechanisms, and diseases. Dev Cell, 17, 9-26.

McKay MM & Morrison DK (2007). Integrating signals from RTKs to ERK/MAPK. Oncogene, 26, 3113-21.

Oldham WM & Hamm HE (2008). Heterotrimeric G protein activation by G-protein-coupled receptors. Nat Rev Mol Cell Biol, 9, 60-71.

Edit history

Date	Action	Author
2005-04-01	Created	Joshi-Tope G
2005-05-06	Authored	Joshi-Tope G, Charalambous M, Gopinathrao G, Rothfels K, Bevan AP et al.
2022-05-18	Reviewed	Barroso I, Rush MG, Joutel A, Stanley FM
2022-05-21	Modified	Weiser JD

100 submitted entities found in this pathway, mapping to 108 Reactome entities

Input	UniProt Id	Input	UniProt Id	Input	UniProt Id
O14686	O14686	O14746	O14746	O14757	O14757
O15264	O15264	O15524	O15524	O43184	O43184
O43524	O43524	O60346	O60346	P00533	P00533
P01111	P01111	P01116	P01116, P01116-1, P01116-2	P02452	P02452
P02461	P02461	P02751	P02751	P04083	P04083
P04198	P04198	P04626	P04626	P04637	P04637
P05106	P05106	P05771	P05771	P06213	P06213
P07900	P07900	P07948	P07948	P08123	P08123
P08151	P08151	P08581	P08581	P09486	P09486
P09544	P09544	P09619	P09619	P09958	P09958
P10071	P10071	P10415	P10415	P10721	P10721
P11274	P11274	P11362	P11362-1, P11362-19	P12110	P12110
P14859	P14859	P16234	P16234	P17252	P17252
P17948	P17948	P21802	P21802-1, P21802-18, P21802-3, P21802-5	P21860	P21860-1
P22681	P22681	P23771	P23771	P27986	P27986
P28336	P28336	P29474	P29474	P29590	P29590
P35222	P35222	P35568	P35568	P35916	P35916
P36543	P36543	P37173	P37173	P40763	P40763
P42224	P42224, P42224-1	P42336	P42336	P42345	P42345
P46531	P46531	P46940	P46940	P49454	P49454
P49619	P49619	P49674	P49674	P49815	P49815
P51812	P51812	P52292	P52292	P52735	P52735
P55211	P55211	P56945	P56945	P60484	P60484
P63092	P63092	P63096	P63096	P78504	P78504
P98177	P98177	Q00987	Q00987	Q01974	Q01974
Q03001	Q03001	Q05513	Q05513	Q05655	Q05655
Q06124	Q06124	Q07812	Q07812	Q09472	Q09472
Q13145	Q13145	Q13164	Q13164	Q13485	Q13485
Q13635	Q13635	Q14185	Q14185	Q14738	Q14738
Q15119	Q15119	Q15465	Q15465	Q16760	Q16760
Q7Z5R6	Q7Z5R6	Q86UP2	Q86UP2	Q92574	Q92574
Q969H0	Q969H0-1, Q969H0-4	Q96GD4	Q96GD4	Q99081	Q99081
Q9NQC7	Q9NQC7	Q9NRY4	Q9NRY4	Q9UPN9	Q9UPN9
Q9Y4A5	Q9Y4A5				

5. PI3K/AKT Signaling in Cancer (R-HSA-2219528)

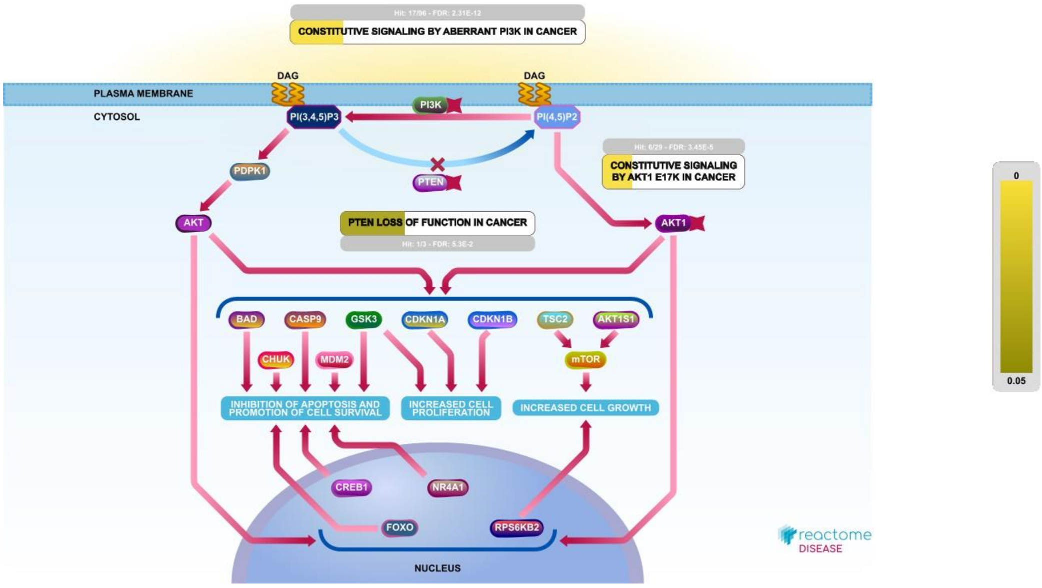

Diseases: cancer.

Class IA PI3K is a heterodimer of a p85 regulatory subunit (encoded by PIK3R1, PIK3R2 or PIK3R3) and a p110 catalytic subunit (encoded by PIK3CA, PIK3CB or PIK3CD). In the absence of activating signals, the regulatory subunit stabilizes the catalytic subunit while inhibiting its activity. The complex becomes activated when extracellular signals stimulate the phosphorylation of the cytoplasmic domains of transmembrane receptors or receptor-associated proteins. The p85 regulatory subunit binds phosphorylated motifs of activator proteins, which induces a conformational change that relieves p85-mediated inhibition of the p110 catalytic subunit and enables PI3K to phosphorylate PIP2 to form PIP3. The phosphoinositide kinase activity of PI3K is opposed by the phosphoinositide phosphatase activity of PTEN.

PIP3 acts as a messenger that recruits PDPK1 (PDK1) and AKT (AKT1, AKT2 or AKT3) to the plasma membrane. PDPK1 also possesses a low affinity for PIP2, so small amounts of PDPK1 are always present at the membrane. Binding of AKT to PIP3 induces a conformational change that enables TORC2 complex to phosphorylate AKT at a conserved serine residue (S473 in AKT1). Phosphorylation at the serine residue enables AKT to bind to PDPK1 and exposes a conserved threonine residue (T308) that is phosphorylated by PDPK1. AKT phosphorylated at both serine and threonine residues dissociates from the plasma membrane and acts as a serine/threonine kinase that phosphorylates a number of cytosolic and nuclear targets involved in regulation of cell metabolism, survival and gene expression. For a recent review, please refer to Manning and Cantley, 2007.

Signaling by PI3K/AKT is frequently constitutively activated in cancer. This activation can be via gain-of-function mutations in PI3KCA (encoding catalytic subunit p110alpha), PIK3R1 (encoding regulatory subunit p85alpha) and AKT1. The PI3K/AKT pathway can also be constitutively activated by loss-of-function mutations in tumor suppressor genes such as PTEN.

Gain-of-function mutations activate PI3K signaling by diverse mechanisms. Mutations affecting the helical domain of PIK3CA and mutations affecting nSH2 and iSH2 domains of PIK3R1 impair inhibitory interactions between these two subunits while preserving their association. Mutations in the catalytic domain of PIK3CA enable the kinase to achieve an active conformation. PI3K complexes with gain-of-function mutations therefore produce PIP3 and activate downstream AKT in the absence of growth factors (Huang et al. 2007, Zhao et al. 2005, Miled et al. 2007, Horn et al. 2008, Sun et al. 2010, Jaiswal et al. 2009, Zhao and Vogt 2010, Urick et al. 2011). While AKT1 gene copy number, expression level and phosphorylation are often increased in cancer, only one low frequency point mutation has been repeatedly reported in cancer and functionally studied. This mutation represents a substitution of a glutamic acid residue with lysine at position 17 of AKT1, and acts by enabling AKT1 to bind PIP2. PIP2-bound AKT1 is phosphorylated by TORC2 complex and by PDPK1 that is always present at the plasma membrane, due to low affinity for PIP2. Therefore, E17K substitution abrogates the need for PI3K in AKT1 activation (Carpten et al. 2007, Landgraf et al. 2008).

Loss-of-function mutations affecting the phosphatase domain of PTEN are frequently found in sporadic cancers (Kong et al. 1997, Lee et al. 1999, Han et al. 2000), as well as in PTEN hamartoma tumor syndromes (PHTS) (Marsh et al. 1998). PTEN can also be inactivated by gene deletion or epigenetic silencing, or indirectly by overexpression of microRNAs that target PTEN mRNA (Huse et al. 2009). Cells with deficient PTEN function have increased levels of PIP3, and therefore increased AKT activity. For a recent review, please refer to Hollander et al. 2011.

Because of their clear involvement in human cancers, PI3K and AKT are targets of considerable interest in the development of small molecule inhibitors. Although none of the currently available inhibitors display preference for mutant variants of PIK3CA or AKT, several inhibitors targeting the wild-type kinases are undergoing clinical trials. These include dual PI3K/mTOR inhibitors, class I PI3K inhibitors, pan-PI3K inhibitors, and pan-AKT inhibitors. While none have yet been approved for clinical use, these agents show promise for future therapeutics. In addition, isoform-specific PI3K and AKT inhibitors are currently being developed, and may provide more specific treatments along with reduced side-effects. For a recent review, please refer to Liu et al. 2009.

References

Roberts TM, Cheng H, Liu P & Zhao JJ (2009). Targeting the phosphoinositide 3-kinase pathway in cancer. Nat Rev Drug Discov, 8, 627-44.

Blumenthal GM, Dennis PA & Hollander MC (2011). PTEN loss in the continuum of common cancers, rare syndromes and mouse models. Nat. Rev. Cancer, 11, 289-301.

Kato H, Shibata H, Matsuno S, Shiiba K, Han SY, Kato S, ... Suzuki T (2000). Functional evaluation of PTEN missense mutations using in vitro phosphoinositide phosphatase assay. Cancer Res, 60, 3147-51.

Liu Z, Shin E, Roberts TM, Wang L, Loda MF & Zhao JJ (2005). The oncogenic properties of mutant p110alpha and p110beta phosphatidylinositol 3-kinases in human mammary epithelial cells. Proc. Natl. Acad. Sci. U.S.A., 102, 18443-8.

Zou TT, Abraham JM, Kong D, Horii A, Yamakawa H, Suzuki A, ... Wakatsuki S (1997). PTEN1 is frequently mutated in primary endometrial carcinomas. Nat. Genet., 17, 143-4.

Edit history

Date	Action	Author
2012-05-01	Created	Orlic-Milacic M
2012-07-18	Authored	Orlic-Milacic M
2012-08-03	Edited	Matthews L
2012-08-13	Reviewed	Yuzugullu H, Thorpe L, Zhao JJ
2020-11-11	Modified	Matthews L

20 submitted entities found in this pathway, mapping to 24 Reactome entities

Input	UniProt Id	Input	UniProt Id	Input	UniProt Id
O43524	O43524	P00533	P00533	P04626	P04626
P08581	P08581	P09619	P09619	P10721	P10721
P11362	P11362-1, P11362-19	P16234	P16234	P21802	P21802-1, P21802-18, P21802-3, P21802-5
P21860	P21860-1	P27986	P27986	P35568	P35568
P42336	P42336	P42345	P42345	P49815	P49815
P55211	P55211	P60484	P60484	P98177	P98177
Q00987	Q00987	Q06124	Q06124		

6. Intracellular signaling by second messengers (R-HSA-9006925)

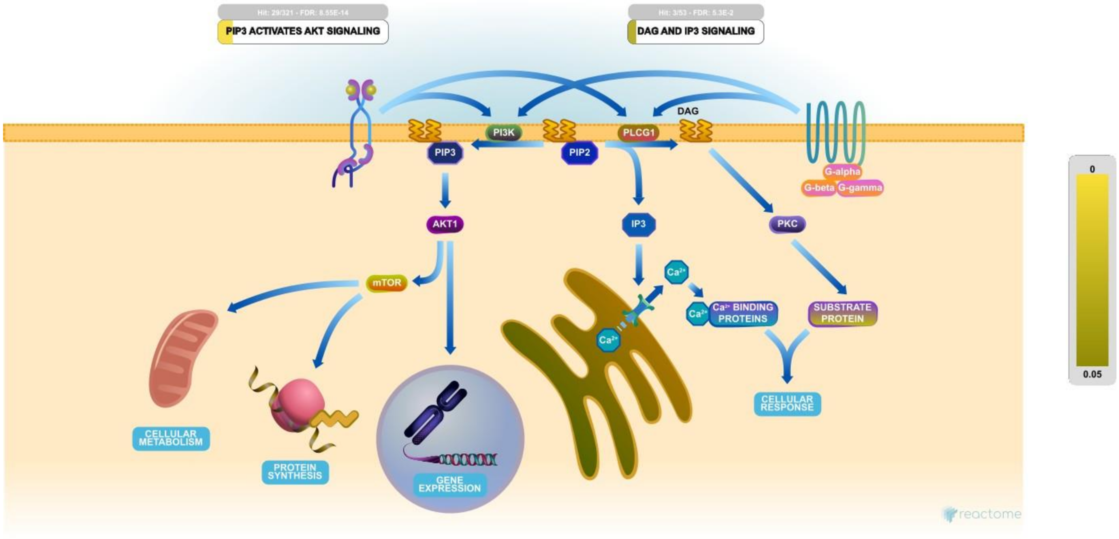

Second messengers are generated within the cell as a downstream step in signal transduction cascades initiated by the interaction of an external stimulus with a cell surface receptor. Common second messengers include DAG, cAMP, cGMP, IP3, Ca2+ and phosphatidylinositols (reviewed in Kang et al, 2015; Raker et al, 2016; Li and Marshall, 2015; Pinto et al, 2015; Ahmad et al, 2015).

References

Kim S, Kang DS, Lee C, Suh PG, Yang YR & Ryu SH (2016). Roles of phosphoinositide-specific phospholipase C1 in brain development. Adv Biol Regul, 60, 167-73.

Ulrich H, Gomes KN, Goulart VA, Kihara AH, Resende RR, Pinto MC & Tonelli FM (2015). Calcium signaling and cell proliferation. Cell. Signal., 27, 2139-49.

Becker C, Steinbrink K & Raker VK (2016). The cAMP Pathway as Therapeutic Target in Autoimmune and Inflammatory Diseases. Front Immunol, 7, 123.

Patel S & Levine TP (2016). Signalling at membrane contact sites: two membranes come together to handle second messengers. Curr. Opin. Cell Biol., 39, 77-83.

Murata T, Ahmad F, Degerman E, Shimizu K, Manganiello V & Maurice D (2015). Cyclic nucleotide phosphodiesterases: important signaling modulators and therapeutic targets. Oral Dis, 21, e25-50.

Edit history

Date	Action	Author
2017-05-24	Edited	Rothfels K
2017-05-24	Authored	Rothfels K
2017-05-24	Created	Rothfels K
2017-06-22	Reviewed	D'Eustachio P
2022-05-21	Modified	Weiser JD

28 submitted entities found in this pathway, mapping to 32 Reactome entities

Input	UniProt Id	Input	UniProt Id	Input	UniProt Id
O43524	O43524	O60346	O60346	P00533	P00533
P04626	P04626	P04637	P04637	P06213	P06213
P08581	P08581	P09619	P09619	P10721	P10721
P11362	P11362-1, P11362-19	P16234	P16234	P17252	P17252
P21802	P21802-1, P21802-18, P21802-3, P21802-5	P21860	P21860-1	P27986	P27986
P29590	P29590	P35568	P35568	P42336	P42336
P42345	P42345	P49815	P49815	P52292	P52292
P55211	P55211	P60484	P60484	P98177	P98177
Q00987	Q00987	Q05655	Q05655	Q06124	Q06124
Q14738	Q14738				

7. Negative regulation of the PI3K/AKT network (R-HSA-199418)

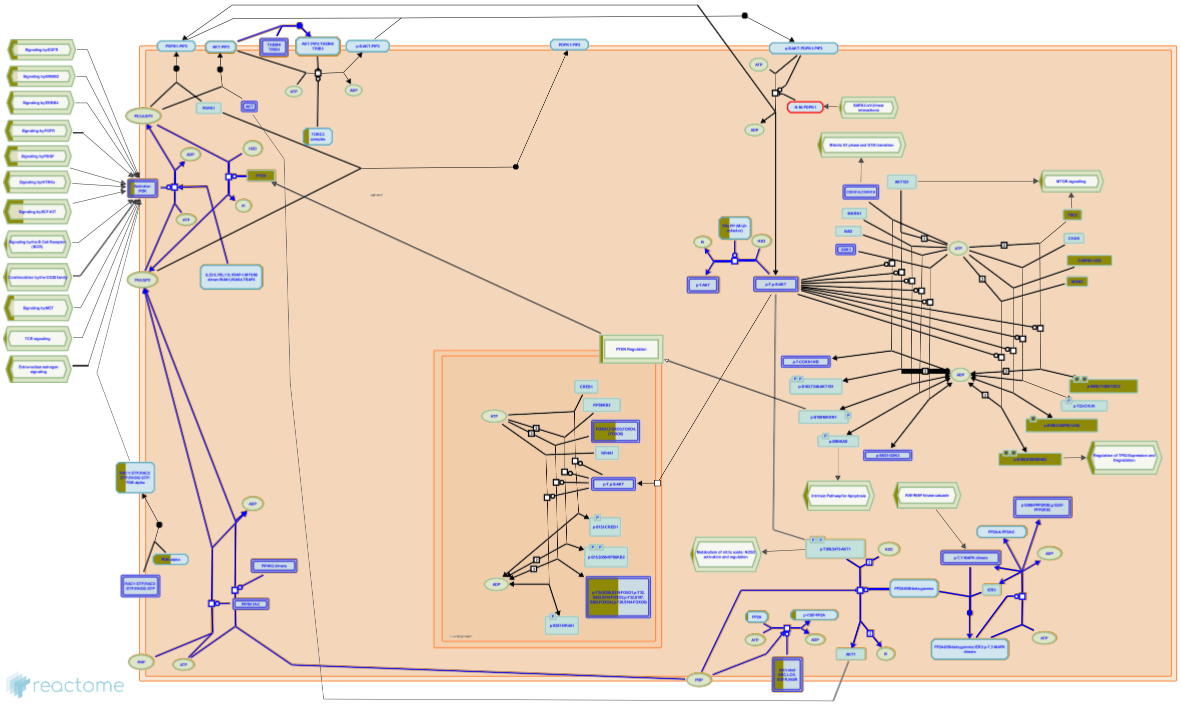

The PI3K/AKT network is negatively regulated by phosphatases that dephosphorylate PIP3, thus hampering AKT activation.

References

Edit history

Date	Action	Author
2006-10-10	Authored	Annibali D, Nasi S
2007-07-10	Created	Jassal B
2007-11-08	Reviewed	Greene LA
2012-06-21	Revised	Orlic-Milacic M
2012-08-13	Reviewed	Yuzugullu H, Thorpe L, Zhao JJ
2022-06-07	Modified	Weiser JD

17 submitted entities found in this pathway, mapping to 21 Reactome entities

Input	UniProt Id	Input	UniProt Id	Input	UniProt Id
O60346	O60346	P00533	P00533	P04626	P04626
P06213	P06213	P08581	P08581	P09619	P09619
P10721	P10721	P11362	P11362-1, P11362-19	P16234	P16234
P21802	P21802-1, P21802-18, P21802-3, P21802-5	P21860	P21860-1	P27986	P27986
P35568	P35568	P42336	P42336	P60484	P60484
Q06124	Q06124	Q14738	Q14738		

8. PIP3 activates AKT signaling (R-HSA-1257604)

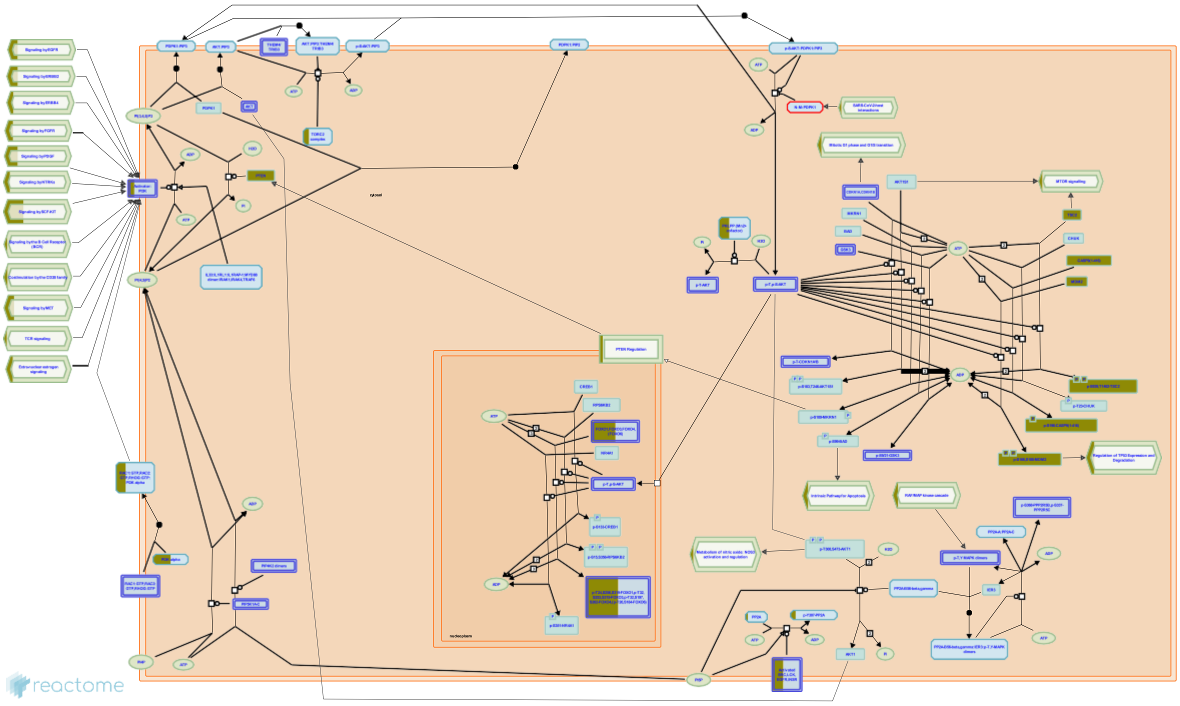

Signaling by AKT is one of the key outcomes of receptor tyrosine kinase (RTK) activation. AKT is activated by the cellular second messenger PIP3, a phospholipid that is generated by PI3K. In ustimulated cells, PI3K class IA enzymes reside in the cytosol as inactive heterodimers composed of p85 regulatory subunit and p110 catalytic subunit. In this complex, p85 stabilizes p110 while inhibiting its catalytic activity. Upon binding of extracellular ligands to RTKs, receptors dimerize and undergo autophosphorylation. The regulatory subunit of PI3K, p85, is recruited to phosphorylated cytosolic RTK domains either directly or indirectly, through adaptor proteins, leading to a conformational change in the PI3K IA heterodimer that relieves inhibition of the p110 catalytic subunit. Activated PI3K IA phosphorylates PIP2, converting it to PIP3; this reaction is negatively regulated by PTEN phosphatase. PIP3 recruits AKT to the plasma membrane, allowing TORC2 to phosphorylate a conserved serine residue of AKT. Phosphorylation of this serine induces a conformation change in AKT, exposing a conserved threonine residue that is then phosphorylated by PDPK1 (PDK1). Phosphorylation of both the threonine and the serine residue is required to fully activate AKT. The active AKT then dissociates from PIP3 and phosphorylates a number of cytosolic and nuclear proteins that play important roles in cell survival and metabolism. For a recent review of AKT signaling, please refer to Manning and Cantley, 2007.

References

Edit history

Date	Action	Author
2007-11-08	Reviewed	Greene LA
2011-05-02	Created	Orlic-Milacic M
2012-06-21	Revised	Orlic-Milacic M
2012-08-13	Reviewed	Yuzugullu H, Thorpe L, Zhao JJ
2022-05-21	Modified	Weiser JD

25 submitted entities found in this pathway, mapping to 29 Reactome entities

Input	UniProt Id	Input	UniProt Id	Input	UniProt Id
O43524	O43524	O60346	O60346	P00533	P00533
P04626	P04626	P04637	P04637	P06213	P06213
P08581	P08581	P09619	P09619	P10721	P10721
P11362	P11362-1, P11362-19	P16234	P16234	P21802	P21802-1, P21802-18, P21802-3, P21802-5
P21860	P21860-1	P27986	P27986	P29590	P29590
P35568	P35568	P42336	P42336	P42345	P42345
P49815	P49815	P55211	P55211	P60484	P60484
P98177	P98177	Q00987	Q00987	Q06124	Q06124
Q14738	Q14738				

9. Signaling by SCF-KIT (R-HSA-1433557)

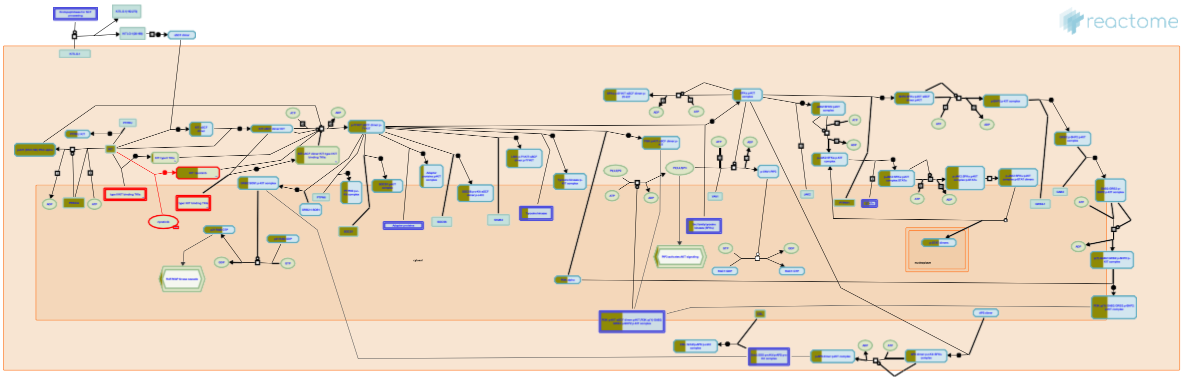

Stem cell factor (SCF) is a growth factor with membrane bound and soluble forms. It is expressed by fibroblasts and endothelial cells throughout the body, promoting proliferation, migration, survival and differentiation of hematopoetic progenitors, melanocytes and germ cells.(Linnekin 1999, Ronnstrand 2004, Lennartsson and Ronnstrand 2006). The receptor for SCF is KIT, a tyrosine kinase receptor (RTK) closely related to the receptors for platelet derived growth factor receptor, colony stimulating factor 1 (Linnekin 1999) and Flt3 (Rosnet et al. 1991). Four isoforms of c-Kit have been identified in humans. Alternative splicing results in isoforms of KIT differing in the presence or absence of four residues (GNNK) in the extracellular region. This occurs due to the use of an alternate 5' splice donor site. These GNNK+ and GNNK- variants are co-expressed in most tissues; the GNNK- form predominates and was more strongly tyrosine-phosphorylated and more rapidly internalized (Ronnstrand 2004). There are also splice variants that arise from alternative usage of splice acceptor site resulting in the presence or absence of a serine residue (Crosier et al., 1993). Finally, there is an alternative shorter transcript of KIT expressed in postmeiotic germ cells in the testis which encodes a truncated KIT consisting only of the second part of the kinase domain and thus lackig the extracellular and transmembrane domains as well as the first part of the kinase domain (Rossi et al. 1991). Binding of SCF homodimers to KIT results in KIT homodimerization followed by activation of its intrinsic tyrosine kinase activity. KIT stimulation activates a wide array of signalling pathways including MAPK, PI3K and JAK/STAT (Reber et al. 2006, Ronnstrand 2004). Defects of KIT in humans are associated with different genetic diseases and also in several types of cancers like mast cell leukaemia, germ cell tumours, certain subtypes of malignant melanoma and gastrointestinal tumours.

References

Lennartsson J & Rönnstrand L (2006). The stem cell factor receptor/c-Kit as a drug target in cancer. Curr Cancer Drug Targets, 6, 65-75.

Frossard N, Da Silva CA & Reber L (2006). Stem cell factor and its receptor c-Kit as targets for inflammatory diseases. Eur J Pharmacol, 533, 327-40.

Rönnstrand L (2004). Signal transduction via the stem cell factor receptor/c-Kit. Cell Mol Life Sci, 61 , 2535-48.

Clark SC, Ricciardi ST, Crosier PS, Vitas MR, Hall LR & Crosier KE (1993). Expression of isoforms of the human receptor tyrosine kinase c-kit in leukemic cell lines and acute myeloid leukemia. Blood, 82, 1151-8.

Hallberg B & Edling CE (2007). c-Kit--a hematopoietic cell essential receptor tyrosine kinase. Int J Biochem Cell Biol, 39, 1995-8.

Edit history

Date	Action	Author
2011-07-11	Edited	Garapati P V
2011-07-11	Authored	Garapati P V
2011-07-11	Created	Garapati P V
2011-08-22	Reviewed	Rönnstrand L
2022-05-20	Modified	Weiser JD

13 submitted entities found in this pathway, mapping to 15 Reactome entities

Input	UniProt Id	Input	UniProt Id	Input	UniProt Id
O14757	O14757	O15524	O15524	P01111	P01111
P01116	P01116-1, P01116-2	P07948	P07948	P10721	P10721
P17252	P17252	P22681	P22681	P27986	P27986
P40763	P40763	P42224	P42224, P42224-1	P42336	P42336
Q06124	Q06124				

10. Signaling by VEGF (R-HSA-194138)

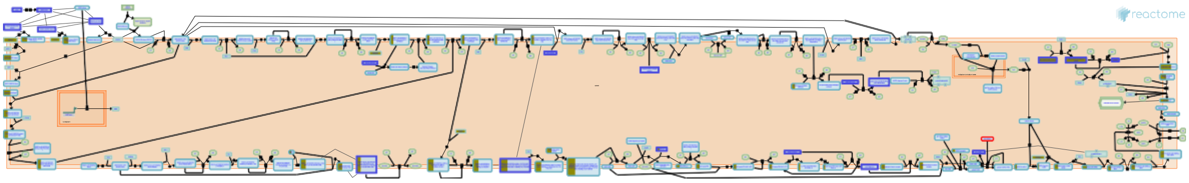

In normal development vascular endothelial growth factors (VEGFs) are crucial regulators of vascular development during embryogenesis (vasculogenesis) and blood-vessel formation in the adult (angiogenesis). In tumor progression, activation of VEGF pathways promotes tumor vascularization, facilitating tumor growth and metastasis. Abnormal VEGF function is also associated with inflammatory diseases including atherosclerosis, and hyperthyroidism. The members of the VEGF and VEGF-receptor protein families have distinct but overlapping ligand-receptor specificities, cell-type expression, and function. VEGF-receptor activation in turn regulates a network of signaling processes in the body that promote endothelial cell growth, migration and survival (Hicklin and Ellis, 2005; Shibuya and Claesson-Welsh, 2006).

Molecular features of the VGF signaling cascades are outlined in the figure below (from Olsson et al. 2006; Nature Publishing Group). Tyrosine residues in the intracellular domains of VEGF receptors 1, 2,and 3 are indicated by dark blue boxes; residues susceptible to phosphorylation are numbered. A circled R indicates that phosphorylation is regulated by cell state (VEGFR2), by ligand binding (VEGFR1), or by heterodimerization (VEGFR3). Specific phosphorylation sites (boxed numbers) bind signaling molecules (dark blue ovals), whose interaction with other cytosolic signaling molecules (light blue ovals) leads to specific cellular (pale blue boxes) and tissue-level (pink boxes) responses in vivo. Signaling cascades whose molecular details are unclear are indicated by dashed arrows. DAG, diacylglycerol; EC, endothelial cell; eNOS, endothelial nitric oxide synthase; FAK, focal adhesion kinase; HPC, hematopoietic progenitor cell; HSP27, heat-shock protein-27; MAPK, mitogen-activated protein kinase; MEK, MAPK and ERK kinase; PI3K, phosphatidylinositol 3' kinase; PKC, protein kinase C; PLCgamma, phospholipase C-gamma; Shb, SH2 and beta-cells; TSAd, T-cell-specific adaptor.

In the current release, the first events in these cascades - the interactions between VEGF proteins and their receptors - are annotated.

References

Shibuya M & Claesson-Welsh L (2006). Signal transduction by VEGF receptors in regulation of angiogenesis and lymphangiogenesis. Exp Cell Res, 312, 549-60.

Ellis LM & Hicklin DJ (2005). Role of the vascular endothelial growth factor pathway in tumor growth and angiogenesis. J Clin Oncol, 23, 1011-27.

Matsumoto T & Mugishima H (2006). Signal transduction via vascular endothelial growth factor (VEGF) receptors and their roles in atherogenesis. J Atheroscler Thromb, 13, 130-5.

Matsumoto T, Cross MJ, Dixelius J & Claesson-Welsh L (2003). VEGF-receptor signal transduction. Trends Biochem Sci, 28, 488-94.

Edit history

Date	Action	Author
2007-03-08	Created	Gopinathrao G

Date	Action	Author
2008-02-28	Reviewed	Claesson-Welsh L
2013-08-30	Edited	Garapati P V
2013-08-30	Authored	Garapati P V
2022-05-21	Modified	Weiser JD

19 submitted entities found in this pathway, mapping to 20 Reactome entities

Input	UniProt Id	Input	UniProt Id	Input	UniProt Id
O15264	O15264	P01111	P01111	P01116	P01116-1, P01116-2
P05106	P05106	P05771	P05771	P07900	P07900
P17252	P17252	P17948	P17948	P27986	P27986
P29474	P29474	P35222	P35222	P35916	P35916
P42336	P42336	P42345	P42345	P52735	P52735
P56945	P56945	Q05513	Q05513	Q05655	Q05655
Q14185	Q14185				

11. Disease (R-HSA-1643685)

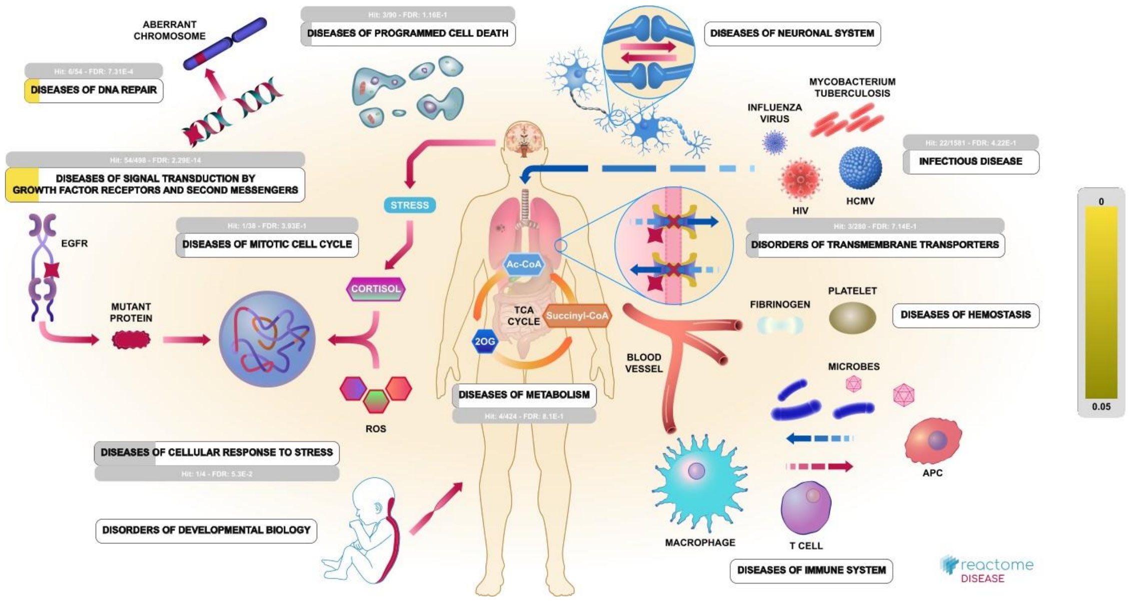

Biological processes are captured in Reactome by identifying the molecules (DNA, RNA, protein, small molecules) involved in them and describing the details of their interactions. From this molecular viewpoint, human disease pathways have three mechanistic causes: the inclusion of microbially-expressed proteins, altered functions of human proteins, or changed expression levels of otherwise functionally normal human proteins.

The first group encompasses the infectious diseases such as influenza, tuberculosis and HIV infection. The second group involves human proteins modified either by a mutation or by an abnormal post-translational event that produces an aberrant protein with a novel function. Examples include somatic mutations of EGFR and FGFR (epidermal and fibroblast growth factor receptor) genes, which encode constitutively active receptors that signal even in the absence of their ligands, or the somatic mutation of IDH1 (isocitrate dehydrogenase 1) that leads to an enzyme active on 2-oxoglutarate rather than isocitrate, or the abnormal protein aggregations of amyloidosis which lead to diseases such as Alzheimer's.

Infectious diseases are represented in Reactome as microbial-human protein interactions and the consequent events. The existence of variant proteins and their association with disease-specific biological processes is represented by inclusion of the modified protein in a new or variant reaction, an extension to the 'normal' pathway. Diseases which result from proteins performing their normal functions but at abnormal rates can also be captured, though less directly. Many mutant alleles encode proteins that retain their normal functions but have abnormal stabilities or catalytic efficiencies, leading to normal reactions that proceed to abnormal extents. The phenotypes of such diseases can be revealed when pathway annotations are combined with expression or rate data from other sources.

Depending on the biological pathway/process immediately affected by disease-causing gene variants, non-infectious diseases in Reactome are organized into diseases of signal transduction by growth factore receptors and second messengers, diseases of mitotic cell cycle, diseases of cellular response to stress, diseases of programmed cell death, diseases of DNA repair, disorders of transmembrane transporters, diseases of metabolism, diseases of immune system, diseases of neuronal system, disorders of developmental biology, disorders of extracellular matrix organization, and diseases of hemostatis.

References

Edit history

Date	Action	Author
2011-10-11	Created	Matthews L
2020-08-24	Edited	Orlic-Milacic M
2021-05-10	Modified	Orlic-Milacic M

69 submitted entities found in this pathway, mapping to 81 Reactome entities

Input	UniProt Id	Input	UniProt Id	Input	UniProt Id
O15164	O15164	O15528	O15528	O43524	O43524
O60934	O60934	P00533	P00533	P01111	P01111
P01116	P01116, P01116-1, P01116-2	P02751	P02751	P02788	P02788
P04626	P04626	P04637	P04637	P04843	P04843
P05106	P05106	P06400	P06400	P07900	P07900
P07948	P07948	P08581	P08581	P09619	P09619
P09958	P09958	P10721	P10721	P11168	P11168
P11274	P11274	P11309	P11309	P11362	P11362, P11362-1, P11362-19
P16234	P16234	P20810	P20810	P21675	P21675
P21802	P21802, P21802-1, P21802-17, P21802-18, P21802-3, P21802-5	P21860	P21860-1	P22681	P22681
P27986	P27986	P29590	P29590	P35222	P35222
P35568	P35568	P37173	P37173	P40692	P40692
P40763	P40763	P42224	P42224, P42224-1, P42224-2	P42336	P42336
P42345	P42345	P42771	P42771	P46531	P46531
P46940	P46940	P49815	P49815	P51587	P51587
P52292	P52292	P52735	P52735	P54278	P54278
P55211	P55211	P60484	P60484	P63092	P63092
P63096	P63096	P78504	P78504	P82987	P82987
P98177	P98177	Q00987	Q00987	Q06124	Q06124
Q09472	Q09472	Q13315	Q13315	Q13485	Q13485
Q13535	Q13535	Q14185	Q14185	Q14738	Q14738
Q15465	Q15465	Q7Z5R6	Q7Z5R6	Q969H0	Q969H0-1, Q969H0-4
Q99758	Q99758	Q9UHD2	Q9UHD2	Q9UHW9	Q9UHW9

12. PI5P, PP2A and IER3 Regulate PI3K/AKT Signaling (R-HSA-6811558)

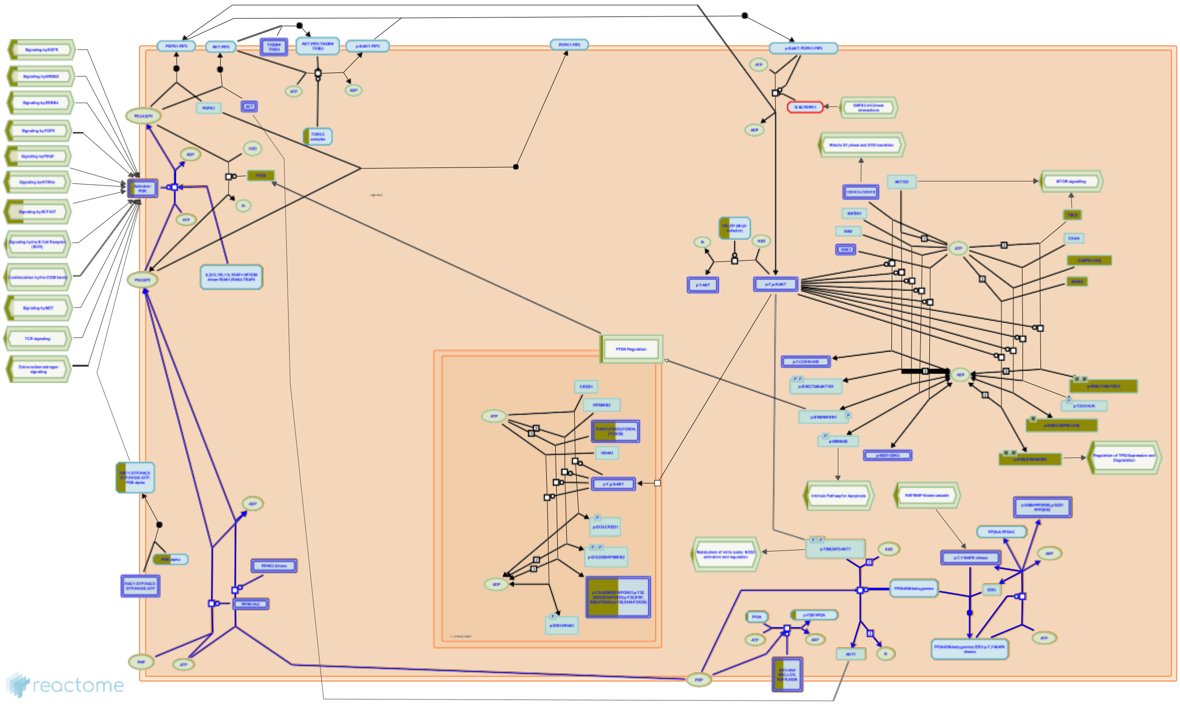

Phosphatidylinositol-5-phosphate (PI5P) may modulate PI3K/AKT signaling in several ways. PI5P is used as a substrate for production of phosphatidylinositol-4,5-bisphosphate, PI(4,5)P2 (Rameh et al. 1997, Clarke et al. 2008, Clarke et al. 2010, Clarke and Irvine 2013, Clarke et al. 2015), which serves as a substrate for activated PI3K, resulting in the production of PIP3 (Mandelker et al. 2009, Burke et al. 2011). The majority of PI(4,5)P2 in the cell, however, is produced from the phosphatidylinositol-4-phosphate (PI4P) substrate (Zhang et al. 1997, Di Paolo et al. 2002, Oude Weernink et al. 2004, Halstead et al. 2006, Oude Weernink et al. 2007). PIP3 is necessary for the activating phosphorylation of AKT. AKT1 can be deactivated by the protein phosphatase 2A (PP2A) complex that contains a regulatory subunit B56-beta (PPP2R5B) or B56-gamma (PPP2R5C). PI5P inhibits AKT1 dephosphorylation by PP2A through an unknown mechanism (Ramel et al. 2009). Increased PI5P levels correlate with inhibitory phosphorylation(s) of the PP2A complex. MAPK1 (ERK2) and MAPK3 (ERK1) are involved in inhibitory phosphorylation of PP2A, in a process that involves IER3 (IEX-1) (Letourneux et al. 2006, Rocher et al. 2007). It is uncertain, however, whether PI5P is in any way involved in ERK-mediated phosphorylation of PP2A or if it regulates another PP2A kinase.

References

Irvine RF & Clarke JH (2013). Evolutionarily conserved structural changes in phosphatidylinositol 5-phosphate 4-kinase (PI5P4K) isoforms are responsible for differences in enzyme activity and localization. Biochem. J., 454, 49-57.

Leslie N, Ramel D, Chicanne G, Gaits-Iacovoni F, Lagarrigue F, Tronchère H, ... Dupuis-Coronas S (2009). PtdIns5P protects Akt from dephosphorylation through PP2A inhibition. Biochem. Biophys. Res. Commun., 387, 127-31.

Thum O, Majerus PW, Parker GJ, Boronenkov IV, Zhang X, Prestwich GD, ... Norris FA (1997). Phosphatidylinositol-4-phosphate 5-kinase isozymes catalyze the synthesis of 3-phosphate-containing phosphatidylinositol signaling molecules. J Biol Chem, 272, 17756-61.

Rocher G, Porteu F & Letourneux C (2006). B56-containing PP2A dephosphorylate ERK and their activity is controlled by the early gene IEX-1 and ERK. EMBO J, 25, 727-38.

Vadas O, Burke JE, Finegan T, Williams RL, Perisic O & Berndt A (2011). Dynamics of the phosphoinositide 3-kinase p110? interaction with p85? and membranes reveals aspects of regulation distinct from p110?. Structure, 19, 1127-37.

Edit history

Date	Action	Author
2015-11-19	Created	Orlic-Milacic M
2015-12-22	Edited	Orlic-Milacic M
2015-12-22	Authored	Orlic-Milacic M
2016-02-08	Reviewed	Porteu F
2022-06-07	Modified	Weiser JD

15 submitted entities found in this pathway, mapping to 19 Reactome entities

Input	UniProt Id	Input	UniProt Id	Input	UniProt Id
P00533	P00533	P04626	P04626	P06213	P06213
P08581	P08581	P09619	P09619	P10721	P10721
P11362	P11362-1, P11362-19	P16234	P16234	P21802	P21802-1, P21802-18, P21802-3, P21802-5
P21860	P21860-1	P27986	P27986	P35568	P35568
P42336	P42336	Q06124	Q06124	Q14738	Q14738

13. Constitutive Signaling by Aberrant PI3K in Cancer (R-HSA-2219530)

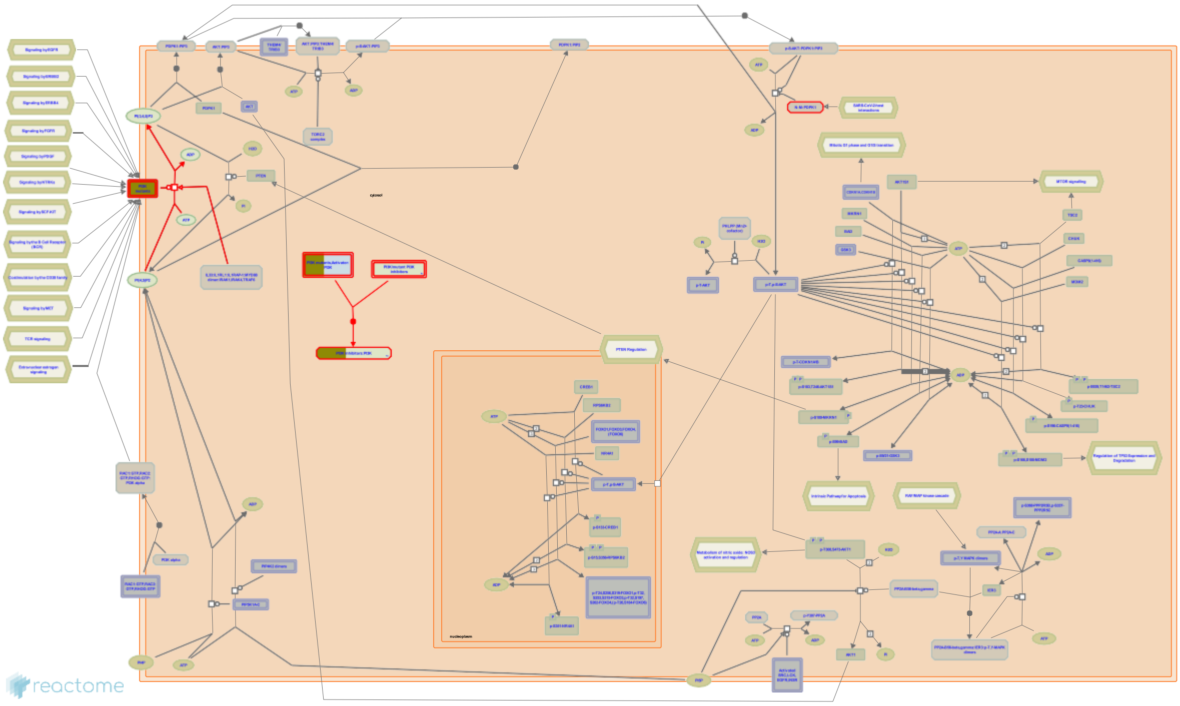

Diseases: cancer.

Signaling by PI3K/AKT is frequently constitutively activated in cancer via gain-of-function mutations in one of the two PI3K subunits - PI3KCA (encoding the catalytic subunit p110alpha) or PIK3R1 (encoding the regulatory subunit p85alpha). Gain-of-function mutations activate PI3K signaling by diverse mechanisms. Mutations affecting the helical domain of PIK3CA and mutations affecting nSH2 and iSH2 domains of PIK3R1 impair inhibitory interactions between these two subunits while preserving their association. Mutations in the catalytic domain of PIK3CA enable the kinase to achieve an active conformation. PI3K complexes with gain-of-function mutations therefore produce PIP3 and activate downstream AKT in the absence of growth factors (Huang et al. 2007, Zhao et al. 2005, Miled et al. 2007, Horn et al. 2008, Sun et al. 2010, Jaiswal et al. 2009, Zhao and Vogt 2010, Urick et al. 2011).

References

Urick ME, Rudd ML, Godwin AK, Sgroi D, Bell DW & Merino M (2011). PIK3R1 (p85?) is somatically mutated at high frequency in primary endometrial cancer. Cancer Res., 71, 4061-7.

Samuels Y, Mandelker D, Schmidt-Kittler O, Kinzler KW, Vogelstein B, Amzel LM, ... Huang CH (2007). The structure of a human p110alpha/p85alpha complex elucidates the effects of oncogenic PI3Kalpha mutations. Science, 318, 1744-8.

Yue P, Kaminker JS, Kan Z, Modrusan Z, Dbouk HA, Kenski DM, ... Li H (2009). Somatic mutations in p85alpha promote tumorigenesis through class IA PI3K activation. Cancer Cell, 16, 463-74.

Inbar Y, Miled N, Hon WC, Williams RL, Yan Y, Backer JM, ... Perisic O (2007). Mechanism of two classes of cancer mutations in the phosphoinositide 3-kinase catalytic subunit. Science, 317, 239-42.

Zhao L & Vogt PK (2010). Hot-spot mutations in p110alpha of phosphatidylinositol 3-kinase (pI3K): differential interactions with the regulatory subunit p85 and with RAS. Cell Cycle, 9, 596-600.

Edit history

Date	Action	Author
2012-05-01	Created	Orlic-Milacic M
2012-07-18	Authored	Orlic-Milacic M
2012-08-13	Reviewed	Yuzugullu H, Thorpe L, Zhao JJ
2022-06-07	Modified	Weiser JD

13 submitted entities found in this pathway, mapping to 17 Reactome entities

Input	UniProt Id	Input	UniProt Id	Input	UniProt Id
P00533	P00533	P04626	P04626	P08581	P08581
P09619	P09619	P10721	P10721	P11362	P11362-1, P11362-19
P16234	P16234	P21802	P21802-1, P21802-18, P21802-3, P21802-5	P21860	P21860-1
P27986	P27986	P35568	P35568	P42336	P42336
Q06124	Q06124				

14. VEGFA-VEGFR2 Pathway (R-HSA-4420097)

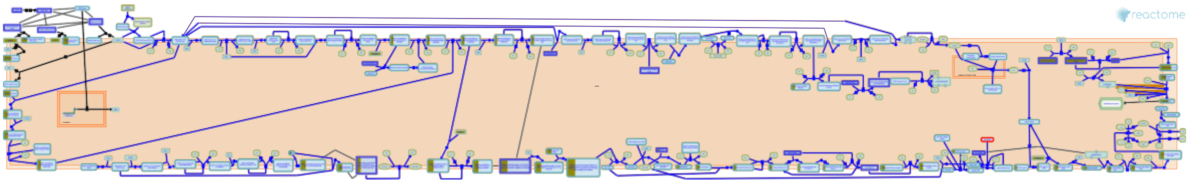

Cellular compartments: plasma membrane.

Angiogenesis is the formation of new blood vessels from preexisting vasculature. One of the most important proangiogenic factors is vascular endothelial growth factor (VEGF). VEGF exerts its biologic effect through interaction with transmembrane tyrosine kinase receptors VEGFR, selectively expressed on vascular endothelial cells. VEGFA signaling through VEGFR2 is the major pathway that activates angiogenesis by inducing the proliferation, survival, sprouting and migration of endothelial cells (ECs), and also by increasing endothelial permeability (Lohela et al. 2009, Shibuya & Claesson-Welsh 2006, Claesson-Welsh & Welsh, 2013). The critical role of VEGFR2 in vascular development is highlighted by the fact that VEGFR2-/- mice die at E8.5-9.5 due to defective development of blood islands, endothelial cells and haematopoietic cells (Shalaby et al. 1995).

References

Makarem JA, Shamseddine AI & Otrock ZK (2007). Vascular endothelial growth factor family of ligands and receptors: review. Blood Cells Mol. Dis., 38, 258-68.

Lohela M, Alitalo K, Tammela T & Bry M (2009). VEGFs and receptors involved in angiogenesis versus lymphangiogenesis. Curr. Opin. Cell Biol., 21, 154-65.

Dimberg A, Kreuger J, Olsson AK & Claesson-Welsh L (2006). VEGF receptor signalling - in control of vascular function. Nat Rev Mol Cell Biol, 7, 359-71.

Matsumoto T, Cross MJ, Dixelius J & Claesson-Welsh L (2003). VEGF-receptor signal transduction. Trends Biochem Sci, 28, 488-94.

Edit history

Date	Action	Author
2013-08-30	Edited	Garapati P V
2013-08-30	Authored	Garapati P V
2013-08-30	Created	Garapati P V
2014-05-12	Reviewed	Welsh M, Berger P, Ballmer-Hofer K
2022-05-21	Modified	Weiser JD

17 submitted entities found in this pathway, mapping to 18 Reactome entities

Input	UniProt Id	Input	UniProt Id	Input	UniProt Id
O15264	O15264	P01111	P01111	P01116	P01116-1, P01116-2
P05106	P05106	P05771	P05771	P07900	P07900
P17252	P17252	P27986	P27986	P29474	P29474
P35222	P35222	P42336	P42336	P42345	P42345
P52735	P52735	P56945	P56945	Q05513	Q05513
Q05655	Q05655	Q14185	Q14185		

15. MAPK1/MAPK3 signaling (R-HSA-5684996)

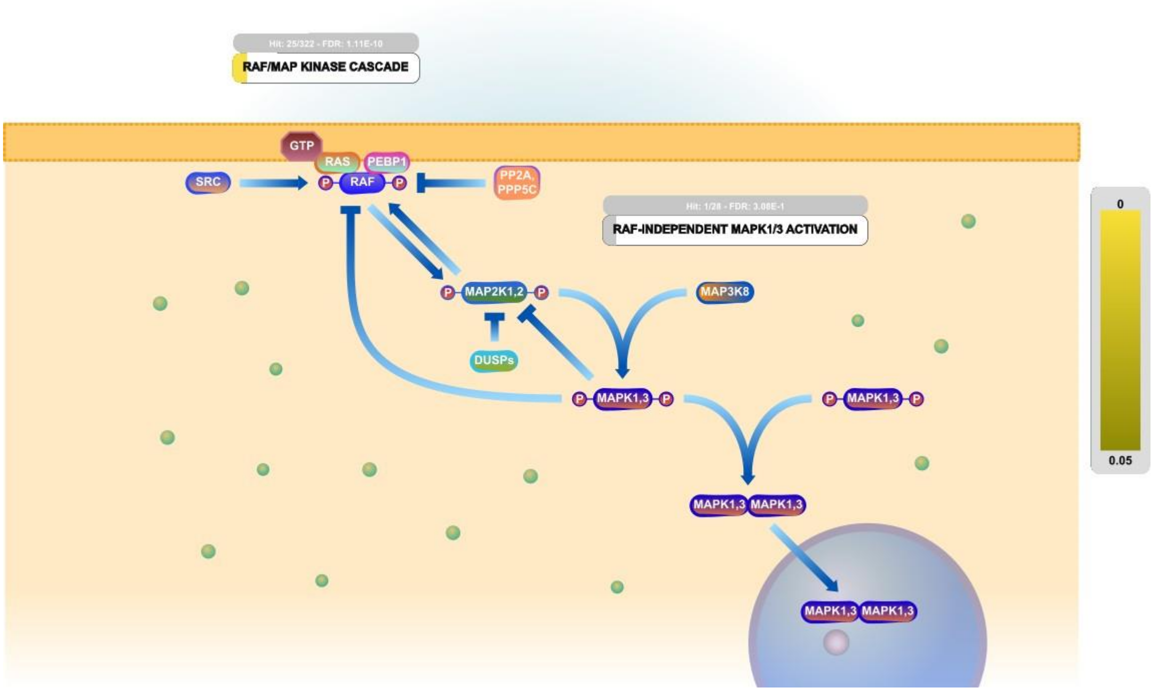

The extracellular signal regulated kinases (ERKs) 1 and 2, also known as MAPK3 and MAPK1, are phosphorylated by the MAP2Ks 1 and 2 in response to a wide range of extracellular stimuli to promote differentiation, proliferation, cell motility, cell survivial, metabolism and transcription, among others (reviewed in Roskoski, 2012b; McKay and Morrison, 2007; Raman et al, 2007). In the classical pathway, MAPK1/3 activation is triggered by the GEF-mediated activation of RAS at the plasma membrane, leading to the activation of the RAF MAP3Ks (reviewed in McKay and Morrison, 2007; Matallanas et al, 2011; Wellbrock et al, 2004). However, many physiological and pathological stimuli have been found to activate MAPK1/3 independently of RAF and RAS, acting instead through MAP3Ks such as MOS, TPL2 and AMPK (Dawson et al, 2008; Wang et al, 2009; Kuriakose et al, 2014; Awane et al, 1999). Activated MAPK1/3 phosphorylate numerous targets in both the nucleus and cytoplasm (reviewed in Yoon and Seger, 2006; Roskoski 2012b).

References

Wellbrock C, Karasarides M & Marais R (2004). The RAF proteins take centre stage. Nat Rev Mol Cell Biol, 5, 875-85.

Whiteman MW, Lian H, Denmark T, Huang D, Wang J, Singh A & Wang G (2009). A non-canonical MEK/ERK signaling pathway regulates autophagy via regulating Beclin 1. J. Biol. Chem., 284, 21412-24.

Morris MA, Tramoutanis G, Laverick L, Dawson CW & Young LS (2008). Epstein-Barr virus-encoded LMP1 regulates epithelial cell motility and invasion via the ERK-MAPK pathway. J. Virol., 82, 3654-64.

Kuriakose T, Rada B & Watford WT (2014). Tumor progression locus 2-dependent oxidative burst drives phosphorylation of extracellular signal-regulated kinase during TLR3 and 9 signaling. J. Biol. Chem., 289, 36089-100.

Chen W, Raman M & Cobb MH (2007). Differential regulation and properties of MAPKs. Oncogene, 26, 3100-12.

Edit history

Date	Action	Author
2015-03-11	Authored	Rothfels K
2015-03-24	Created	Rothfels K
2015-04-29	Reviewed	Roskoski R Jr
2022-05-21	Modified	Weiser JD

20 submitted entities found in this pathway, mapping to 26 Reactome entities

Input	UniProt Id	Input	UniProt Id	Input	UniProt Id
P00533	P00533	P01111	P01111	P01116	P01116, P01116-1, P01116-2
P02751	P02751	P04626	P04626	P05106	P05106
P08581	P08581	P09619	P09619	P10721	P10721
P11362	P11362-1, P11362-19	P16234	P16234	P21802	P21802-1, P21802-18, P21802-3, P21802-5
P21860	P21860-1	P27986	P27986	P35568	P35568
P42336	P42336	P46940	P46940	Q06124	Q06124
Q14738	Q14738	Q7Z5R6	Q7Z5R6		

16. Signaling by PDGF (R-HSA-186797)

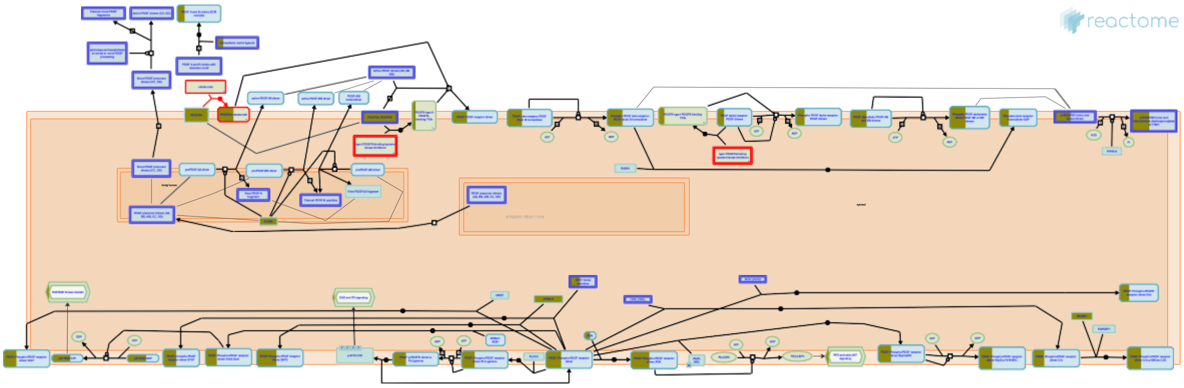

Platelet-derived Growth Factor (PDGF) is a potent stimulator of growth and motility of connective tissue cells such as fibroblasts and smooth muscle cells as well as other cells such as capillary endothelial cells and neurons.The PDGF family of growth factors is composed of four different polypeptide chains encoded by four different genes. The classical PDGF chains, PDGF-A and PDGF-B, and more recently discovered PDGF-C and PDGF-D. The four PDGF chains assemble into disulphide-bonded dimers via homo- or heterodimerization, and five different dimeric isoforms have been described so far; PDGF-AA, PDGF-AB, PDGF-BB, PDGF-CC and PDGF-DD. It is notable that no heterodimers involving PDGF-C and PDGF-D chains have been described. PDGF exerts its effects by binding to, and activating, two protein tyrosine kinase (PTK) receptors, alpha and beta. These receptors dimerize and undergo autophosphorylation. The phosphorylation sites then attract downstream effectors to transduct the signal into the cell.

References

Li H, Eriksson U & Fredriksson L (2004). The PDGF family: four gene products form five dimeric isoforms. Cytokine Growth Factor Rev, 15, 197-204.

Ostman A, Heldin CH & Rönnstrand L (1998). Signal transduction via platelet-derived growth factor receptors. Biochim Biophys Acta, 1378, F79-113.

Westermark B & Heldin CH (1999). Mechanism of action and in vivo role of platelet-derived growth factor. Physiol Rev, 79, 1283-316.

Edit history

Date	Action	Author
2006-08-15	Created	Jassal B
2008-11-24	Edited	Jassal B, Garapati P V
2008-11-24	Reviewed	Heldin CH
2008-11-24	Authored	Jassal B, Garapati P V
2022-06-07	Modified	Weiser JD

13 submitted entities found in this pathway, mapping to 14 Reactome entities

Input	UniProt Id	Input	UniProt Id	Input	UniProt Id
P01111	P01111	P01116	P01116-1, P01116-2	P02461	P02461
P09619	P09619	P09958	P09958	P12110	P12110

Input	UniProt Id	Input	UniProt Id	Input	UniProt Id
P16234	P16234	P27986	P27986	P40763	P40763
P42224	P42224	P42336	P42336	P56945	P56945
Q06124	Q06124				

17. Insulin receptor signalling cascade (R-HSA-74751)

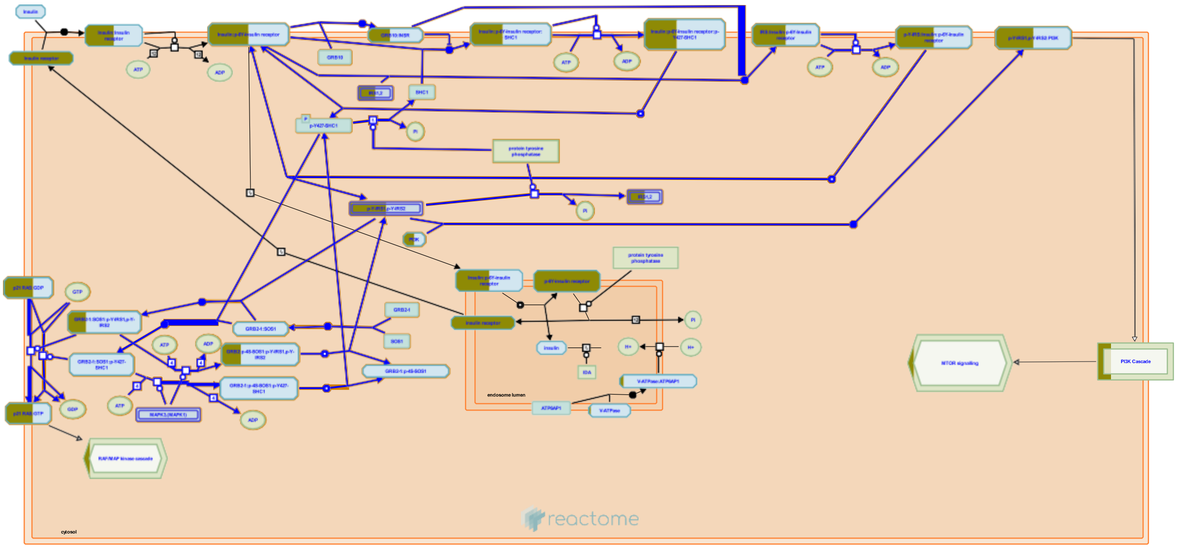

Cellular compartments: cytosol.

Autophosphorylation of the insulin receptor triggers a series of signalling events, mediated by SHC or IRS, and resulting in activation of the Ras/RAF and MAP kinase cascades. A second effect of the autophosphorylation of the insulin receptor is its internalisation into an endosome, which down-regulates its signalling activity.

References

Bevan P (2001). Insulin signalling. J Cell Sci, 114, 1429-30.

Shepherd PR, Siddle K & Withers DJ (1998). Phosphoinositide 3-kinase: the key switch mechanism in insulin signalling. Biochem J, 333, 471-90.

Edit history

Date	Action	Author
2003-07-31	Authored	Bevan AP
2003-07-31	Created	Bevan AP
2022-06-07	Modified	Weiser JD

9 submitted entities found in this pathway, mapping to 14 Reactome entities

Input	UniProt Id	Input	UniProt Id	Input	UniProt Id
P01111	P01111	P01116	P01116-1, P01116-2	P06213	P06213
P11362	P11362-1, P11362-19	P21802	P21802-1, P21802-18, P21802-3, P21802-5	P27986	P27986
P35568	P35568	P42336	P42336	Q06124	Q06124

18. MAPK family signaling cascades (R-HSA-5683057)

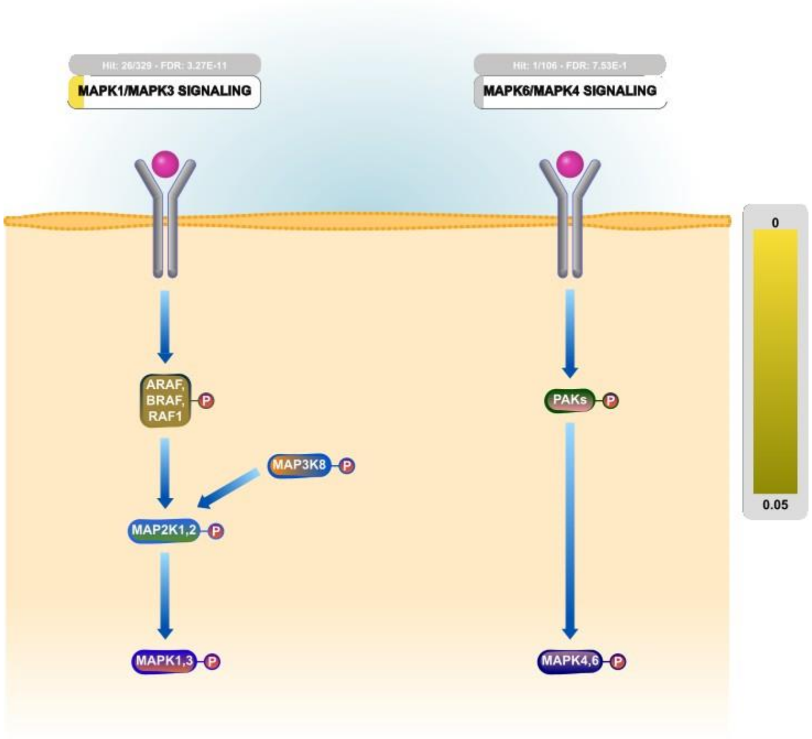

The mitogen activated protein kinases (MAPKs) are a family of conserved protein serine threonine kinases that respond to varied extracellular stimuli to activate intracellular processes including gene expression, metabolism, proliferation, differentiation and apoptosis, among others.

The classic MAPK cascades, including the ERK1/2 pathway, the p38 MAPK pathway, the JNK pathway and the ERK5 pathway are characterized by three tiers of sequentially acting, activating kinases (reviewed in Kryiakis and Avruch, 2012; Cargnello and Roux, 2011). The MAPK kinase kinase kinase (MAPKKK), at the top of the cascade, is phosphorylated on serine and threonine residues in response to external stimuli; this phosphorylation often occurs in the context of an interaction between the MAPKKK protein and a member of the RAS/RHO family of small GTP-binding proteins. Activated MAPKKK proteins in turn phosphorylate the dual-specificity MAPK kinase proteins (MAPKK), which ultimately phosphorylate the MAPK proteins in a conserved Thr-X-Tyr motif in the activation loop.

Less is known about the activation of the atypical families of MAPKs, which include the ERK3/4 signaling cascade, the ERK7 cascade and the NLK cascade. Although the details are not fully worked out, these MAPK proteins don't appear to be phosphorylated downstream of a 3-tiered kinase system as described above (reviewed in Coulombe and Meloche, 2007; Cargnello and Roux, 2011) .

Both conventional and atypical MAPKs are proline-directed serine threonine kinases and, once activated, phosphorylate substrates in the consensus P-X-S/T-P site. Both cytosolic and nuclear targets of MAPK proteins have been identified and upon stimulation, a proportion of the phosphorylated MAPKs relocalize from the cytoplasm to the nucleus. In some cases, nuclear translocation may be accompanied by dimerization, although the relationship between these two events is not fully elaborated (reviewed in Kryiakis and Avruch, 2012; Cargnello and Roux, 2011; Plotnikov et al, 2010).

References

Meloche S & Coulombe P (2007). Atypical mitogen-activated protein kinases: structure, regulation and functions. Biochim. Biophys. Acta, 1773, 1376-87.

Kyriakis JM & Avruch J (2012). Mammalian MAPK signal transduction pathways activated by stress and inflammation: a 10-year update. Physiol. Rev., 92, 689-737.

Zehorai E, Procaccia S, Seger R & Plotnikov A (2011). The MAPK cascades: signaling components, nuclear roles and mechanisms of nuclear translocation. Biochim. Biophys. Acta, 1813, 1619-33.

Roux PP & Cargnello M (2011). Activation and function of the MAPKs and their substrates, the MAPK-activated protein kinases. Microbiol. Mol. Biol. Rev., 75, 50-83.

Edit history

Date	Action	Author
2015-03-10	Authored	Rothfels K
2015-03-11	Created	Rothfels K
2015-04-29	Reviewed	Roskoski R Jr
2022-05-21	Modified	Weiser JD

21 submitted entities found in this pathway, mapping to 27 Reactome entities

Input	UniProt Id	Input	UniProt Id	Input	UniProt Id
O43524	O43524	P00533	P00533	P01111	P01111
P01116	P01116, P01116-1, P01116-2	P02751	P02751	P04626	P04626
P05106	P05106	P08581	P08581	P09619	P09619
P10721	P10721	P11362	P11362-1, P11362-19	P16234	P16234
P21802	P21802-1, P21802-18, P21802-3, P21802-5	P21860	P21860-1	P27986	P27986
P35568	P35568	P42336	P42336	P46940	P46940
Q06124	Q06124	Q14738	Q14738	Q7Z5R6	Q7Z5R6

19. RAF/MAP kinase cascade (R-HSA-5673001)

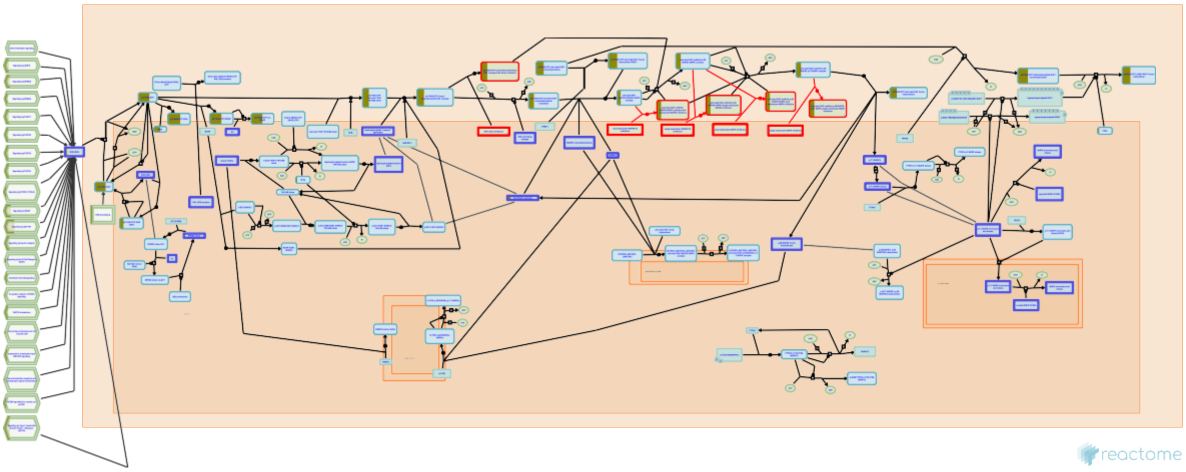

The RAS-RAF-MEK-ERK pathway regulates processes such as proliferation, differentiation, survival, senescence and cell motility in response to growth factors, hormones and cytokines, among others. Binding of these stimuli to receptors in the plasma membrane promotes the GEF-mediated activation of RAS at the plasma membrane and initiates the three-tiered kinase cascade of the conventional MAPK cascades. GTP-bound RAS recruits RAF (the MAPK kinase kinase), and promotes its dimerization and activation (reviewed in Cseh et al, 2014; Roskoski, 2010; McKay and Morrison, 2007; Wellbrock et al, 2004). Activated RAF phosphorylates the MAPK kinase proteins MEK1 and MEK2 (also known as MAP2K1 and MAP2K2), which in turn phophorylate the proline-directed kinases ERK1 and 2 (also known as MAPK3 and MAPK1) (reviewed in Roskoski, 2012a, b; Kryiakis and Avruch, 2012). Activated ERK proteins may undergo dimerization and have identified targets in both the nucleus and the cytosol; consistent with this, a proportion of activated ERK protein relocalizes to the nucleus in response to stimuli (reviewed in Roskoski 2012b; Turjanski et al, 2007; Plotnikov et al, 2010; Cargnello et al, 2011). Although initially seen as a linear cascade originating at the plasma membrane and culminating in the nucleus, the RAS/RAF MAPK cascade is now also known to be activated from various intracellular location. Temporal and spatial specificity of the cascade is achieved in part through the interaction of pathway components with numerous scaffolding proteins (reviewed in McKay and Morrison, 2007; Brown and Sacks, 2009).

The importance of the RAS/RAF MAPK cascade is highlighted by the fact that components of this pathway are mutated with high frequency in a large number of human cancers. Activating mutations in RAS are found in approximately one third of human cancers, while ~8% of tumors express an activated form of BRAF (Roberts and Der, 2007; Davies et al, 2002; Cantwell-Dorris et al, 2011).

References

Roskoski R Jr (2012). MEK1/2 dual-specificity protein kinases: structure and regulation. Biochem. Biophys. Res. Commun., 417, 5-10.

Brown MD & Sacks DB (2009). Protein scaffolds in MAP kinase signalling. Cell. Signal., 21, 462-9.

Cantwell-Dorris ER, Sheils OM & O'Leary JJ (2011). BRAFV600E: implications for carcinogenesis and molecular therapy. Mol. Cancer Ther., 10, 385-94.

Maitland N, Jayatilake H, Futreal PA, Yuen ST, Marshall CJ, Menzies A, ... Pritchard-Jones K (2002). Mutations of the BRAF gene in human cancer. Nature, 417, 949-54.

Gutkind JS, Turjanski AG & Vaqué JP (2007). MAP kinases and the control of nuclear events. Oncogene, 26, 3240-53.

Edit history

Date	Action	Author
2015-02-06	Created	Rothfels K
2015-02-12	Edited	Rothfels K
2015-02-12	Authored	Rothfels K
2015-04-29	Reviewed	Roskoski R Jr
2022-06-07	Modified	Weiser JD

19 submitted entities found in this pathway, mapping to 25 Reactome entities

Input	UniProt Id	Input	UniProt Id	Input	UniProt Id
P00533	P00533	P01111	P01111	P01116	P01116, P01116-1, P01116-2
P02751	P02751	P04626	P04626	P05106	P05106
P08581	P08581	P09619	P09619	P10721	P10721
P11362	P11362-1, P11362-19	P16234	P16234	P21802	P21802-1, P21802-18, P21802-3, P21802-5
P21860	P21860-1	P27986	P27986	P35568	P35568
P42336	P42336	P46940	P46940	Q14738	Q14738
Q7Z5R6	Q7Z5R6				

20. Signaling by FGFR1 in disease (R-HSA-5655302)

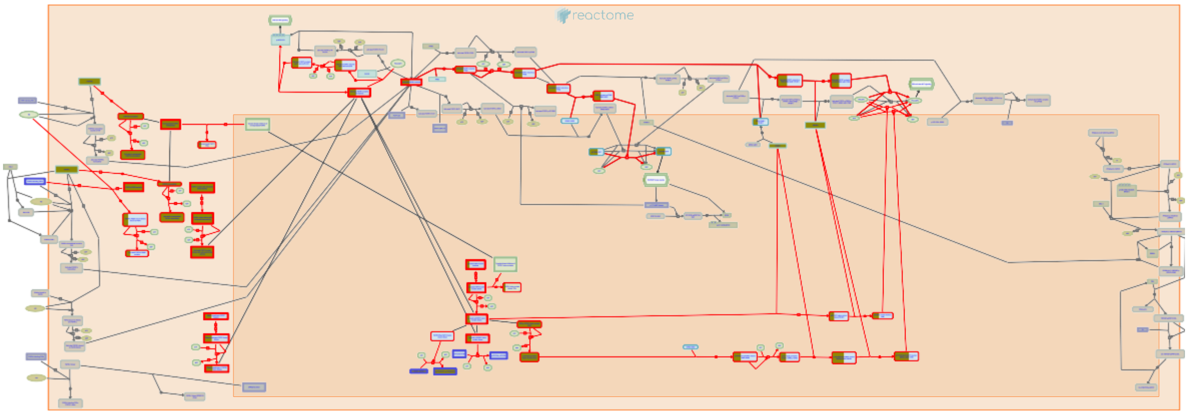

Diseases: cancer, bone development disease.

The FGFR1 gene has been shown to be subject to activating mutations, chromosomal rearrangements and gene amplification leading to a variety of proliferative and developmental disorders depending on whether these events occur in the germline or arise somatically (reviewed in Webster and Donoghue, 1997; Burke, 1998; Cunningham, 2007; Wesche, 2011; Greulich and Pollock, 2011).

Activating mutation P252R in FGFR1 is associated with the development of Pfeiffer syndrome, characterized by craniosynostosis (premature fusion of several sutures in the skull) and broadened thumbs and toes (Muenke, 1994; reviewed in Cunningham, 2007). This residue falls in a highly conserved Pro-Ser dipeptide between the second and third Ig domains of the extracellular region of the receptor. The mutation is thought to increase the number of hydrogen bonds formed with the ligand and to thereby increase ligand-binding affinity (Ibrahimi, 2004a). Unlike other FGF receptors, few activating point mutations in the FGFR1 coding sequence have been identified in cancer. Point mutations in the Ig II-III linker analagous to the P252R Pfeiffer syndrome mutation have been identified in lung cancer and melanoma (Ruhe, 2007; Davies, 2005), and two kinase-domain mutations in FGFR1 have been identified in glioblastoma (Rand, 2005, Network TCGA, 2008).

In contrast, FGFR1 is a target of chromosomal rearrangements in a number of cancers. FGFR1 has been shown to be recurrently translocated in the 8p11 myeloproliferative syndrome (EMS), a preleukemic condition also known as stem cell leukemia/lymphoma (SCLL) that rapidly progresses to leukemia. This translocation fuses the kinase domain of FGFR1 with the dimerization domain of one of 10 identified fusion partners, resulting in the constitutive dimerization and activation of the kinase (reviewed in Jackson, 2010).

Amplification of the FGFR1 gene has been implicated as a oncogenic factor in a range of cancers, including breast, ovarian, bladder, lung, oral squamous carcinomas, and rhabdomyosarcoma (reviewed in Turner and Grose, 2010; Wesche, 2011; Greulich and Pollock, 2011), although there are other candidate genes in the amplified region and the definitive role of FGFR1 has not been fully established.

More recently, FGFR1 fusion proteins have been identified in a number of cancers; these are thought to undergo constitutive ligand-independent dimerization and activation based on dimerization motifs found in the fusion partners (reviewed in Parker, 2014).

References

Cancer Genome Atlas Research Network - (2008). Comprehensive genomic characterization defines human glioblastoma genes and core pathways. Nature, 455, 1061-8.

Pollock PM & Greulich H (2011). Targeting mutant fibroblast growth factor receptors in cancer. Trends Mol Med, 17, 283-92.

Donoghue DJ & Webster MK (1997). FGFR activation in skeletal disorders: too much of a good thing. Trends Genet, 13, 178-82.

Easton DF, Dicks E, Stratton MR, Davies H, Cooper CS, Tofts C, ... Varian J (2005). Somatic mutations of the protein kinase gene family in human lung cancer. Cancer Res, 65, 7591-5.

Burke D, Malcolm S, Blundell TL & Wilkes D (1998). Fibroblast growth factor receptors: lessons from the genes. Trends Biochem Sci, 23, 59-62.

Edit history

Date	Action	Author
2012-05-15	Reviewed	Ezzat S
2014-11-20	Authored	Rothfels K
2014-12-05	Edited	Rothfels K
2014-12-05	Created	Rothfels K
2016-01-22	Modified	Rothfels K

9 submitted entities found in this pathway, mapping to 12 Reactome entities

Input	UniProt Id	Input	UniProt Id	Input	UniProt Id
O15164	O15164	P01111	P01111	P01116	P01116-1, P01116-2
P11274	P11274	P11362	P11362, P11362-1, P11362-19	P27986	P27986
P40763	P40763	P42224	P42224	P42336	P42336

21. Downstream signal transduction (R-HSA-186763)

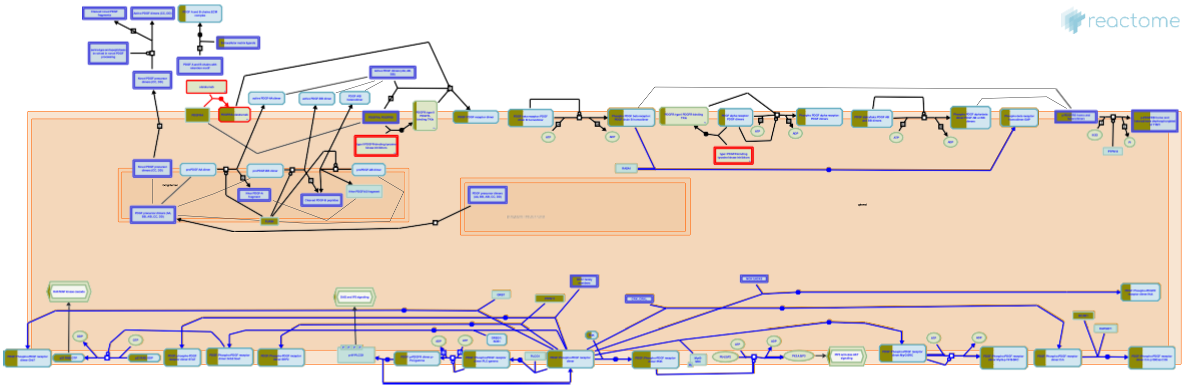

The role of autophosphorylation sites on PDGF receptors are to provide docking sites for downstream signal transduction molecules which contain SH2 domains. The SH2 domain is a conserved motif of around 100 amino acids that can bind a phosphorylated tyrosine residue. These downstream molecules are activated upon binding to, or phosphorylated by, the receptor kinases intrinsic to PDGF receptors.

Some of the dowstream molecules are themselves enzymes, such as phosphatidylinositol 3'-kinase (PI3K), phospholipase C (PLC-gamma), the Src family of tyrosine kinases, the tyrosine phosphatase SHP2, and a GTPase activating protein (GAP) for Ras. Others such as Grb2 are adaptor molecules which link the receptor with downstream catalytic molecules.

References

Westermark B & Heldin CH (1999). Mechanism of action and in vivo role of platelet-derived growth factor. Physiol Rev, 79, 1283-316.

Ostman A, Heldin CH & Rönnstrand L (1998). Signal transduction via platelet-derived growth factor receptors. Biochim Biophys Acta, 1378, F79-113.

Edit history

Date	Action	Author
2006-08-15	Created	Jassal B
2008-11-24	Edited	Jassal B, Garapati P V
2008-11-24	Reviewed	Heldin CH
2008-11-24	Authored	Jassal B, Garapati P V
2022-06-07	Modified	Weiser JD

10 submitted entities found in this pathway, mapping to 11 Reactome entities

Input	UniProt Id	Input	UniProt Id	Input	UniProt Id
P01111	P01111	P01116	P01116-1, P01116-2	P09619	P09619
P16234	P16234	P27986	P27986	P40763	P40763
P42224	P42224	P42336	P42336	P56945	P56945
Q06124	Q06124				

22. Signaling by Insulin receptor (R-HSA-74752)

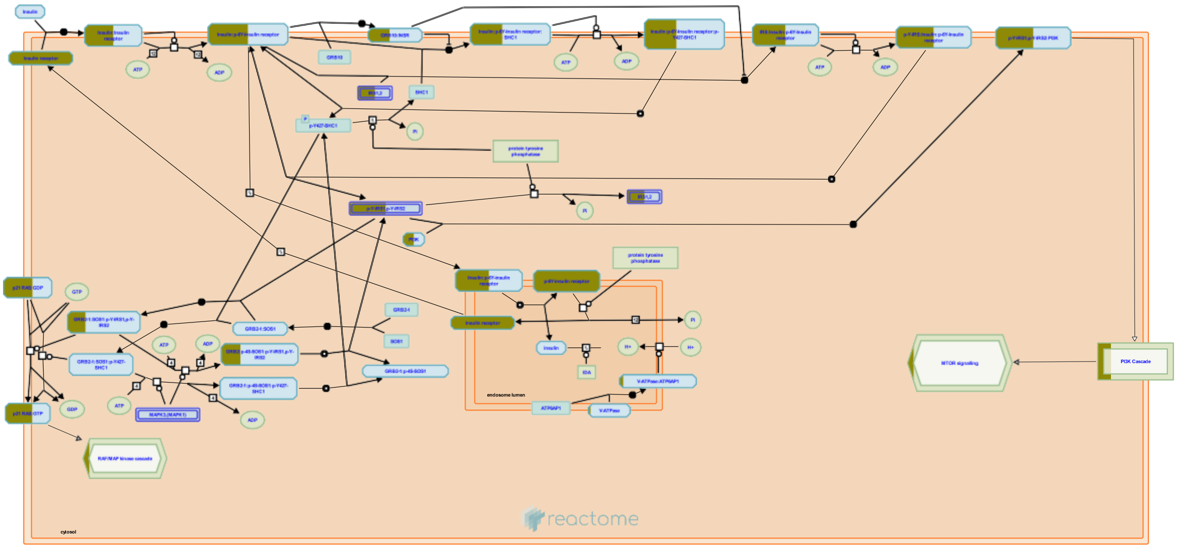

Insulin binding to its receptor results in receptor autophosphorylation on tyrosine residues and the tyrosine phosphorylation of insulin receptor substrates (e.g. IRS and Shc) by the insulin receptor tyrosine kinase. This allows association of IRSs with downstream effectors such as PI-3K via its Src homology 2 (SH2) domains leading to end point events such as Glut4 (Slc2a4) translocation. Shc when tyrosine phosphorylated associates with Grb2 and can thus activate the Ras/MAPK pathway independent of the IRSs.

Signal transduction by the insulin receptor is not limited to its activation at the cell surface. The activated ligand-receptor complex initially at the cell surface, is internalised into endosomes itself a process which is dependent on tyrosine autophosphorylation. Endocytosis of activated receptors has the dual effect of concentrating receptors within endosomes and allows the insulin receptor tyrosine kinase to phosphorylate substrates that are spatially distinct from those accessible at the plasma membrane. Acidification of the endosomal lumen, due to the presence of proton pumps, results in dissociation of insulin from its receptor. (The endosome constitutes the major site of insulin degradation). This loss of the ligand-receptor complex attenuates any further insulin-driven receptor re-phosphorylation events and leads to receptor dephosphorylation by extra-lumenal endosomally-associated protein tyrosine phosphatases (PTPs). The identity of these PTPs is not clearly established yet.

References

White MF & Kahn CR (1994). The insulin signaling system. J Biol Chem, 269, 1-4.

Edit history

Date	Action	Author
2003-07-31	Authored	Bevan AP
2003-07-31	Created	Bevan AP
2022-05-18	Edited	Schmidt EE
2022-05-18	Reviewed	Barroso I, Stanley FM
2022-06-07	Modified	Weiser JD

10 submitted entities found in this pathway, mapping to 15 Reactome entities

Input	UniProt Id	Input	UniProt Id	Input	UniProt Id
P01111	P01111	P01116	P01116-1, P01116-2	P06213	P06213
P11362	P11362-1, P11362-19	P21802	P21802-1, P21802-18, P21802-3, P21802-5	P27986	P27986
P35568	P35568	P36543	P36543	P42336	P42336
Q06124	Q06124				

23. IRS-mediated signalling (R-HSA-112399)

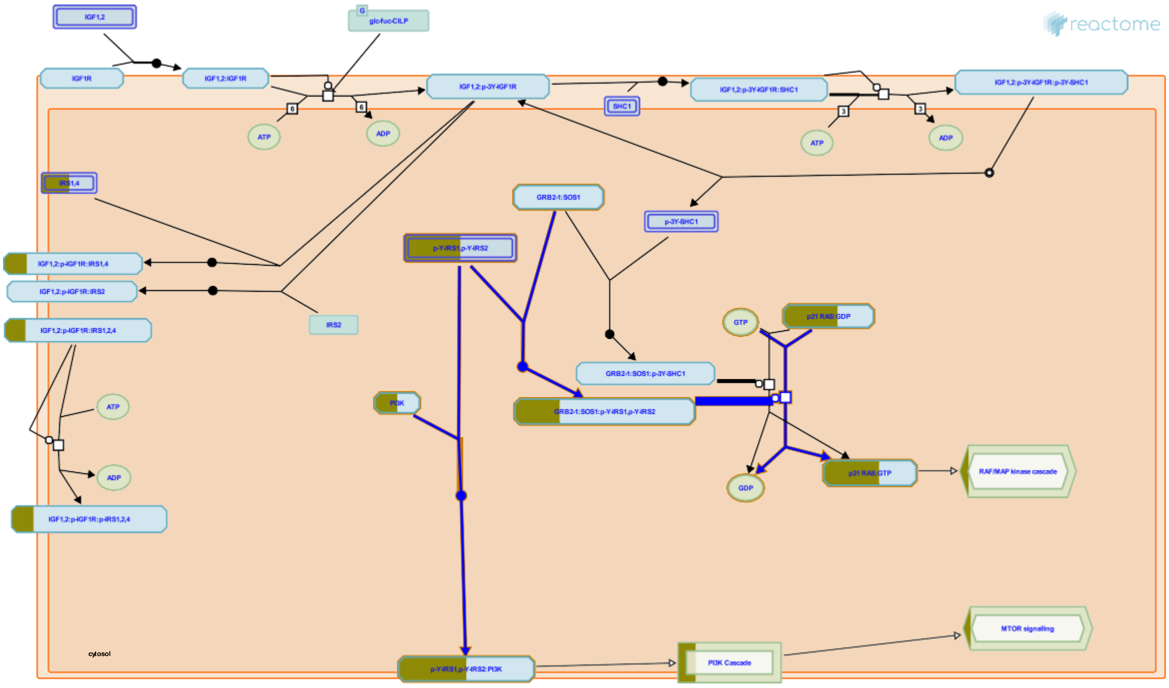

Cellular compartments: plasma membrane, cytosol.

Release of phospho-IRS from the insulin receptor triggers a cascade of signalling events via PI3K, SOS, RAF and the MAP kinases.

References

Edit history

Date	Action	Author
2004-04-29	Authored	Charalambous M
2004-04-29	Created	Charalambous M
2022-06-07	Modified	Weiser JD

8 submitted entities found in this pathway, mapping to 13 Reactome entities

Input	UniProt Id	Input	UniProt Id	Input	UniProt Id
P01111	P01111	P01116	P01116-1, P01116-2	P11362	P11362-1, P11362-19
P21802	P21802-1, P21802-18, P21802-3, P21802-5	P27986	P27986	P35568	P35568
P42336	P42336	Q06124	Q06124		

24. Signaling by KIT in disease (R-HSA-9669938)

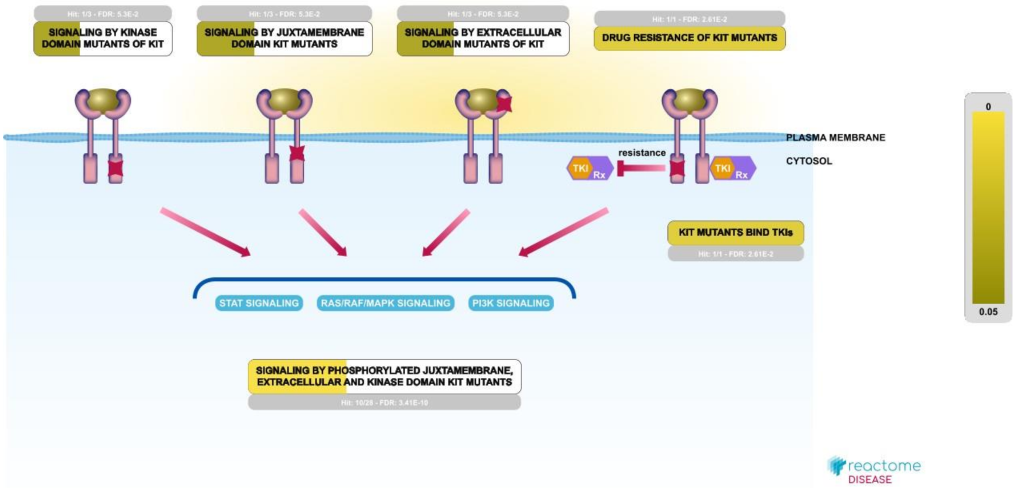

Diseases: cancer.

KIT signaling is important in several processes including stem cell maintenance, erythropoiesis, mast cell development, lymphopoiesis, melanogenesis and maintenance of interstitial cell of Cajal (Hirota et al, 1998; Chi et al, 2010). Gain-of-function mutations in KIT have been identified at low frequency in a number of diseases, including AML, melanoma and mast and germ cell tumors, and at higher frequency in gastrointesinal stromal tumors (reviewed in Lennartsson and Roonstrand, 2012; Abbaspour Babaei et al, 2016; Roskoski, 2018).

References

Ahmadipour F, Abbaspour Babaei M, Huri HZ, Kamalidehghan B & Saleem M (2016). Receptor tyrosine kinase (c-Kit) inhibitors: a potential therapeutic target in cancer cells. Drug Des Devel Ther, 10, 2443-59.

Rönnstrand L & Lennartsson J (2012). Stem cell factor receptor/c-Kit: from basic science to clinical implications. Physiol. Rev., 92, 1619-49.

Dewell S, Antonescu CR, Chen Y, Maki RG, Zhang L, Sawyers CL, ... Wongvipat J (2010). ETV1 is a lineage survival factor that cooperates with KIT in gastrointestinal stromal tumours. Nature, 467 , 849-53.

Roskoski R (2018). The role of small molecule Kit protein-tyrosine kinase inhibitors in the treatment of neoplastic disorders. Pharmacol. Res., 133, 35-52.

Kitamura Y, Muhammad Tunio G, Kanakura Y, Matsuzawa Y, Hanada M, Moriyama Y, ... Shinomura Y (1998). Gain-of-function mutations of c-kit in human gastrointestinal stromal tumors. Science, 279, 577-80.

Edit history

Date	Action	Author
2019-12-03	Created	Rothfels K
2020-03-13	Reviewed	García-Valverde A, Pilco-Janeta D, Serrano C

Date	Action	Author
2020-04-01	Authored	Rothfels K
2020-05-04	Edited	Rothfels K
2021-05-04	Modified	Matthews L

8 submitted entities found in this pathway, mapping to 10 Reactome entities

Input	UniProt Id	Input	UniProt Id	Input	UniProt Id
P01111	P01111	P01116	P01116-1, P01116-2	P07948	P07948
P10721	P10721	P27986	P27986	P40763	P40763
P42224	P42224, P42224-1	P42336	P42336		

25. Signaling by phosphorylated juxtamembrane, extracellular and kinase domain KIT mutants (R-HSA-9670439)

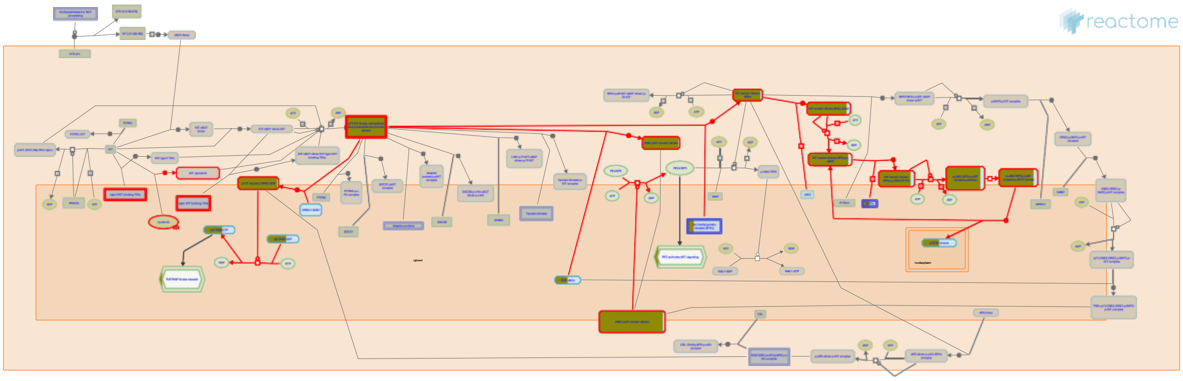

Diseases: cancer.

Activation of the PI3K/mTOR, RAS/MAPK and STAT signaling pathways has been observed downstream of activated extracellular, juxtamembrane and kinase domain mutants of KIT, although downstream signaling has not been studied in great detail in all cases. Activation of these pathways contributes to cellular proliferation, avoidance of apoptosis, and actin cytoskeletal organization (Dunesing et al, 2004; Bauer et al, 2007; Chi et al, 2010; Bosbach et al, 2017; reviewed in Lennartsson and Roonstrand, 2012; Corless et al, 2011).

References

Rönnstrand L & Lennartsson J (2012). Stem cell factor receptor/c-Kit: from basic science to clinical implications. Physiol. Rev., 92, 1619-49.

Dewell S, Antonescu CR, Chen Y, Maki RG, Zhang L, Sawyers CL, ... Wongvipat J (2010). ETV1 is a lineage survival factor that cooperates with KIT in gastrointestinal stromal tumours. Nature, 467 , 849-53.

Barnett CM, Corless CL & Heinrich MC (2011). Gastrointestinal stromal tumours: origin and molecular oncology. Nat. Rev. Cancer, 11, 865-78.

McConarty B, Medeiros F, Fletcher JA, Demetri GD, Singer S, Fletcher CD, ... Duensing A (2004). Mechanisms of oncogenic KIT signal transduction in primary gastrointestinal stromal tumors (GISTs). Oncogene, 23, 3999-4006.

Antonescu CR, Veach D, Ehlers I, Yozgat Y, Warpinski K, Besmer P, ... DeMatteo RP (2017). Direct engagement of the PI3K pathway by mutant KIT dominates oncogenic signaling in gastrointestinal stromal tumor. Proc. Natl. Acad. Sci. U.S.A., 114, E8448-E8457.

Edit history

Date	Action	Author
2019-12-09	Created	Rothfels K
2020-03-13	Reviewed	García-Valverde A, Pilco-Janeta D, Serrano C
2020-04-01	Authored	Rothfels K
2020-05-04	Edited	Rothfels K
2022-06-07	Modified	Weiser JD

8 submitted entities found in this pathway, mapping to 10 Reactome entities

Input	UniProt Id	Input	UniProt Id	Input	UniProt Id
P01111	P01111	P01116	P01116-1, P01116-2	P07948	P07948
P10721	P10721	P27986	P27986	P40763	P40763
P42224	P42224, P42224-1	P42336	P42336		

6. Identifiers found

Below is a list of the input identifiers that have been found or mapped to an equivalent element in Reactome, classified by resource.

167 of the submitted entities were found, mapping to 182 Reactome entities

Input	UniProt Id	Input	UniProt Id	Input	UniProt Id
O00300	O00300	O00533	O00533	O00750	O00750
O14686	O14686	O14746	O14746	O14757	O14757
O15066	O15066	O15151	O15151	O15164	O15164
O15264	O15264	O15439	O15439	O15524	O15524
O15528	O15528	O43184	O43184	O43474	O43474
O43524	O43524	O43602	O43602	O60346	O60346
O60934	O60934	O75382	O75382	O95299	O95299
P00338	P00338	P00533	P00533	P01011	P01011
P01023	P01023	P01111	P01111	P01116	P01116, P01116-1, P01116-2
P02452	P02452	P02461	P02461	P02751	P02751
P02788	P02788	P04083	P04083	P04198	P04198
P04626	P04626	P04637	P04637	P04843	P04843
P04921	P04921	P05106	P05106	P05107	P05107
P05771	P05771	P06213	P06213	P06400	P06400
P07900	P07900	P07948	P07948	P08123	P08123
P08151	P08151	P08247	P08247	P08581	P08581
P08684	P08684	P09486	P09486	P09544	P09544
P09619	P09619	P09668	P09668	P09958	P09958
P10071	P10071	P10415	P10415	P10721	P10721
P11168	P11168	P11274	P11274	P11309	P11309
P11362	P11362, P11362-1, P11362-19	P11387	P11387	P12110	P12110
P14859	P14859	P15529	P15529	P16035	P16035
P16234	P16234	P17252	P17252	P17948	P17948
P20810	P20810	P21675	P21675	P21802	P21802, P21802-1, P21802-17, P21802-18, P21802-3, P21802-5
P21860	P21860-1	P22681	P22681	P23771	P23771
P24821	P24821	P27540	P27540	P27986	P27986
P28329	P28329	P28336	P28336	P29474	P29474
P29590	P29590	P30291	P30291	P35222	P35222
P35568	P35568	P35916	P35916	P36543	P36543
P37173	P37173	P37275	P37275	P40692	P40692
P40763	P40763	P42224	P42224, P42224-1	P42262	P42262
P42336	P42336	P42345	P42345	P42771	P42771
P45984	P45984	P46531	P46531	P46940	P46940
P49454	P49454	P49619	P49619	P49674	P49674
P49815	P49815	P51587	P51587	P51812	P51812
P52292	P52292	P52735	P52735	P54278	P54278
P55211	P55211	P56945	P56945	P60484	P60484
P63092	P63092	P63096	P63096	P78504	P78504
P78527	P78527	P82987	P82987	P98177	P98177

Input	UniProt Id
Q00987	Q00987
Q02548	Q02548
Q05655	Q05655
Q09472	Q09472
Q13164	Q13164
Q13444	Q13444
Q13635	Q13635
Q15119	Q15119
Q6NUQ1	Q6NUQ1
Q8WYB5	Q8WYB5
Q96GD4	Q96GD4
Q96L91	Q96L91
Q99490	Q99490
Q9C040	Q9C040
Q9NRY4	Q9NRY4
Q9UJS0	Q9UJS0
Q9Y4A5	Q9Y4A5

Input	UniProt Id
Q01543	Q01543
Q03001	Q03001
Q06124	Q06124
Q13126	Q13126
Q13315	Q13315
Q13485	Q13485
Q14185	Q14185
Q15465	Q15465
Q7Z5R6	Q7Z5R6
Q92574	Q92574
Q96HY7	Q96HY7
Q99081	Q99081
Q99758	Q99758
Q9HAU5	Q9HAU5
Q9UHD2	Q9UHD2
Q9UNL4	Q9UNL4
Q9Y561	Q9Y561

Input	UniProt Id
Q01974	Q01974
Q05513	Q05513
Q07812	Q07812
Q13145	Q13145
Q13418	Q13418
Q13535	Q13535
Q14738	Q14738
Q16760	Q16760
Q86UP2	Q86UP2
Q969H0	Q969H0-1, Q969H0-4
Q96L34	Q96L34
Q99435	Q99435
Q9BZL6	Q9BZL6
Q9NQC7	Q9NQC7
Q9UHW9	Q9UHW9
Q9UPN9	Q9UPN9

7. Identifiers not found

These 17 identifiers were not found neither mapped to any entity in Reactome.

O43692	O95831	P08922	P35716	Q06455	Q13214	Q16799	Q32MQ5
Q6ZWH5	Q8IWV1	Q8IZT6	Q8TF68	Q92786	Q9BXK5	Q9H0K1	Q9H1R3
Q9NRP7							
