# Supplementary figures and images for: Brown Adipose Tissue Sheds Extracellular Vesicles That Carry Potential Biomarkers of Metabolic and Thermogenesis Activity Which Are Affected by High Fat Diet Intervention
Source: Int J Mol Sci. 2022 Sep 16;23(18):10826. doi: 10.3390/ijms231810826 (PMC9504916; doi:10.3390/ijms231810826)

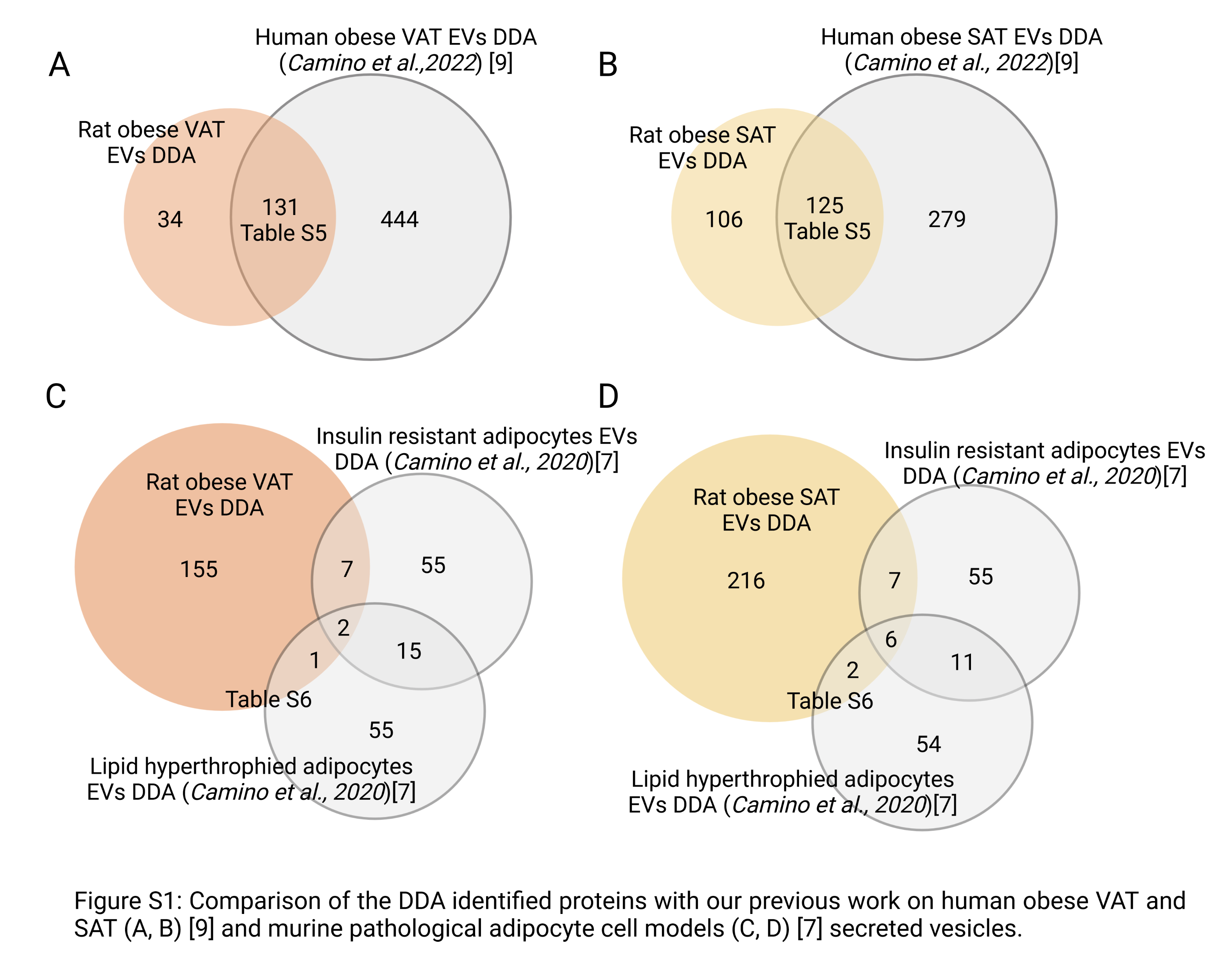

Supplement: Supplementary file 1 [file ijms-23-10826-s001.zip › FIGURE S1.png]

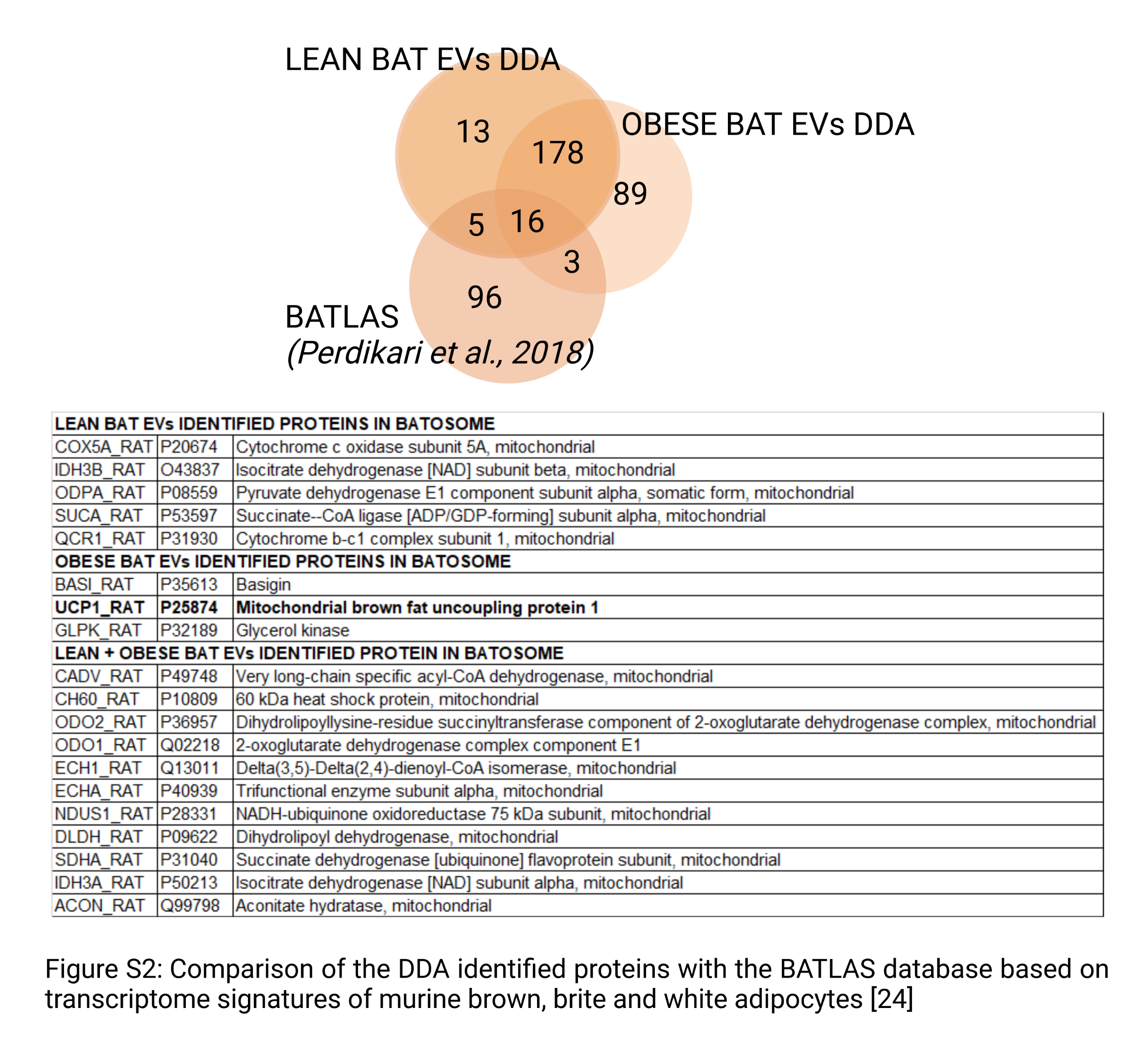

Supplement: Supplementary file 1 [file ijms-23-10826-s001.zip › FIGURE S2.png]

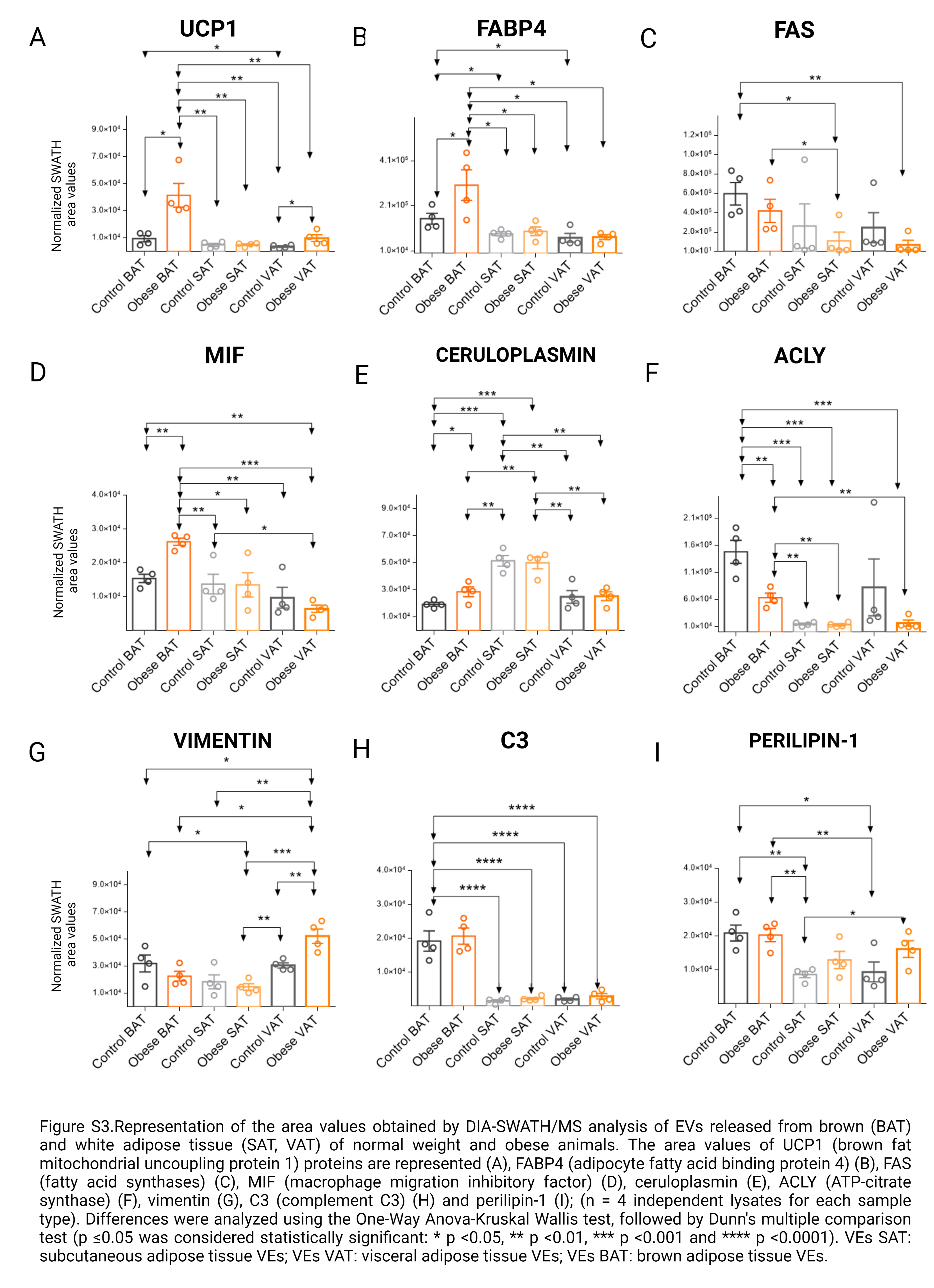

Supplement: Supplementary file 1 [file ijms-23-10826-s001.zip › FIGURE S3.png]
