# Supplementary material for: Brown Adipose Tissue Sheds Extracellular Vesicles That Carry Potential Biomarkers of Metabolic and Thermogenesis Activity Which Are Affected by High Fat Diet Intervention
Source: Int J Mol Sci. 2022 Sep 16;23(18):10826. doi: 10.3390/ijms231810826 (PMC9504916; doi:10.3390/ijms231810826)
Supplement: Supplementary file 1 [file ijms-23-10826-s001.zip › Publication Licenses.zip!/Publication License Sep-07-2022-1 F S2.pdf]

49 Spadina Ave. Suite 200
Toronto ON M5V 2J1 Canada
www.biorender.com

Confirmation of Publication and Licensing Rights

September 7th, 2022
Science Suite Inc.

Subscription:	*Lab*
Agreement number:	*CV24DKITKT*
Journal name:	*International Journal of Molecular Sciences*

To whom this may concern,

This document is to confirm that Maria Pardo has been granted a license to use the BioRender content, including icons, templates and other original artwork, appearing in the attached completed graphic pursuant to BioRender's Academic License Terms. This license permits BioRender content to be sublicensed for use in journal publications.

All rights and ownership of BioRender content are reserved by BioRender. All completed graphics must be accompanied by the following citation: "Created with BioRender.com".

BioRender content included in the completed graphic is not licensed for any commercial uses beyond publication in a journal. For any commercial use of this figure, users may, if allowed, recreate it in BioRender under an Industry BioRender Plan.

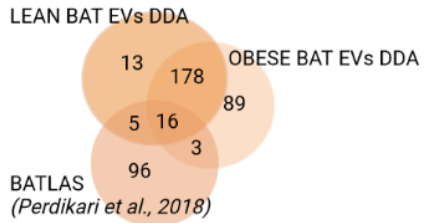

**LEAN BAT EVs IDENTIFIED PROTEINS IN BATOSOME**		
COX5A_RAT	P20674	Cytochrome c oxidase subunit 5A, mitochondrial
IDH3B_RAT	O43837	Isocitrate dehydrogenase [NAD] subunit beta, mitochondrial
ODPA_RAT	P08559	Pyruvate dehydrogenase E1 component subunit alpha, somatic form, mitochondrial
SUCA_RAT	P53597	Succinate--CoA ligase [ADP/GDP-forming] subunit alpha, mitochondrial
QCR1_RAT	P31930	Cytochrome b-c1 complex subunit 1, mitochondrial
**OBESE BAT EVs IDENTIFIED PROTEINS IN BATOSOME**		
BASI_RAT	P35613	Basigin
**UCP1_RAT**	**P25874**	**Mitochondrial brown fat uncoupling protein 1**
GLPK_RAT	P32189	Glycerol kinase
**LEAN + OBESE BAT EVs IDENTIFIED PROTEIN IN BATOSOME**		
CADV_RAT	P49748	Very long-chain specific acyl-CoA dehydrogenase, mitochondrial
CH60_RAT	P10809	60 kDa heat shock protein, mitochondrial
ODO2_RAT	P36957	Dihydrolipoyllysine-residue succinyltransferase component of 2-oxoglutarate dehydrogenase complex, mitochondrial
ODO1_RAT	Q02218	2-oxoglutarate dehydrogenase complex component E1
ECH1_RAT	Q13011	Delta(3,5)-Delta(2,4)-dienoyl-CoA isomerase, mitochondrial
ECHA_RAT	P40939	Trifunctional enzyme subunit alpha, mitochondrial
NDUS1_RAT	P28331	NADH-ubiquinone oxidoreductase 75 kDa subunit, mitochondrial
DLDH_RAT	P09622	Dihydrolipoyl dehydrogenase, mitochondrial
SDHA_RAT	P31040	Succinate dehydrogenase [ubiquinone] flavoprotein subunit, mitochondrial
IDH3A_RAT	P50213	Isocitrate dehydrogenase [NAD] subunit alpha, mitochondrial
ACON_RAT	Q99798	Aconitate hydratase, mitochondrial

Figure S2: Comparison of the DDA identified proteins with the BATLAS database based on transcriptome signatures of murine brown, brite and white adipocytes [24]

For any questions regarding this document, or other questions about publishing with BioRender refer to our BioRender Publication Guide, or contact BioRender Support at support@biorender.com.
